# Comparative Efficacy of Treatments for Resistant or Uncontrolled Hypertension: A Network Meta-Analysis of Randomized Trials

**DOI:** 10.1016/j.jacadv.2026.103306

**Published:** 2026-10-05

**Authors:** Angelo Laino, Raffaele Piccolo, Antonio Pio Vitale, Raffaele Izzo, Anna Franzone, Giovanni Esposito

**Affiliations:** Department of Advanced Biomedical Sciences, University of Naples Federico II, Naples, Italy

**Keywords:** hypertension, resistant hypertension, uncontrolled hypertension

## Abstract

**Background:**

Many patients with hypertension fail to achieve recommended blood pressure targets despite treatment with 2 or more antihypertensive drugs.

**Objectives:**

We aimed to compare the efficacy of add-on treatments for the management of resistant or uncontrolled hypertension.

**Methods:**

We searched Embase and MEDLINE through November 2025. We included randomized trials comparing add-on treatments for resistant or uncontrolled hypertension. The certainty of evidence was assessed using the Confidence In Network Meta-Analysis (CINeMA) framework. The primary outcome was the change in office systolic blood pressure from baseline. Summary mean differences were estimated using random-effects network meta-analysis within a Bayesian framework in patients with resistant hypertension and in the broader population with uncontrolled hypertension.

**Results:**

We included 35 trials (8,648 patients and 12 individual treatments) in the systematic review and 30 trials (8,187 patients and 10 individual treatments) in the network meta-analysis. In patients with resistant hypertension, spironolactone (mean difference: −12.43 mm Hg; 95% credible interval: −17.68 to −7.18; CINeMA moderate) and radiofrequency renal denervation (mean difference: −8.74 mm Hg; 95% credible interval: −12.96 to −4.52; CINeMA moderate) were more effective than baseline treatment in reducing office systolic blood pressure. Similar findings were observed in patients with uncontrolled hypertension. No intervention demonstrated clear superiority over another because most head-to-head comparisons included the possibility of no effect.

**Conclusions:**

In head-to-head comparisons, no significant differences were observed between add-on treatments in reducing blood pressure among patients with resistant or uncontrolled hypertension. Treatment selection should prioritize treatments with demonstrated superiority over baseline treatment, including spironolactone, baxdrostat, and radiofrequency renal denervation.

Hypertension affects 1 of 3 adults worldwide and represents the leading modifiable risk factor for cardiovascular disease. Despite the availability of multiple lines of therapy, <50% of patients achieve an adequate control of blood pressure, leading to a global control rate of disease of only 20%.^1^ Uncontrolled hypertension currently represents a global health issue, and it is projected to become even more relevant in view of the population growth and the progressively lower targets of blood pressure. Indeed, both European and American guidelines currently recommend target blood pressure levels of 120 to 129 mm Hg systolic and 70 to 79 mm Hg diastolic for adults with confirmed hypertension. However, while guidelines provide convergent recommendations on the use of a 2-drug combination approach as the initial therapy for most patients, the subsequent management of patients with resistant or uncontrolled hypertension is more controversial.^2^, ^3^, ^4^, ^5^, ^6^ Evidence from head-to-head comparisons is limited to a small number of relatively underpowered randomized trials,^2^ resulting in persistent knowledge gaps that are reflected in contemporary guidance recommendations for the management of uncontrolled hypertension.^3^^,^^4^

Given the wide range of available therapeutic options, conventional pairwise meta-analyses are inadequate to draw definitive conclusions about the most effective treatment. We aimed to better inform clinical practice by conducting a comprehensive network meta-analysis of randomized trials evaluating add-on therapies for patients with resistant or uncontrolled hypertension.

## Methods

### Search strategy and selection criteria

We did a systematic review and network meta-analysis of randomized trials by following the extension of the PRISMA (Preferred Reporting Items for Systematic Reviews and Meta-Analyses) statement for reporting network meta-analyses (Supplemental Appendix).^5^ Ethical approval was not required as this was a study-level meta-analysis. The data that support the findings of this study are available from the corresponding author upon reasonable request. We searched Embase and MEDLINE from inception to November 2025 (detailed search strings for each database are reported in the Supplemental Appendix), without language restrictions. In addition, we assessed the lists of included studies from related systematic reviews to check if any relevant article was missed.^6^, ^7^, ^8^, ^9^, ^10^

We included blinded and nonblinded randomized trials comparing pharmacologic or device-based interventions for the management of resistant (defined as a systolic blood pressure [SBP] ≥140 mm Hg despite treatment with 3 or more antihypertensive drugs, including a diuretic, at full doses) or uncontrolled (defined as an SBP ≥140 mm Hg despite treatment with 2 or more antihypertensive drugs) hypertension in adult patients. These definitions were based on the most recent European Society of Cardiology hypertension guidelines.^3^ The complete list of the interventions deemed eligible for study inclusion is provided in the Supplemental Appendix. We excluded the following drug classes that are already recommended as first-line treatment by guidelines: angiotensin-converting enzyme inhibitors, angiotensin receptor blockers, calcium channel blockers, beta-blockers, and diuretics. Because these agents are commonly part of the background therapy in patients with resistant or uncontrolled hypertension, trials evaluating these drugs as third- or fourth-line options are infrequent, and including them in the network meta-analysis would have introduced substantial overlap between interventions, thereby hampering the clinical relevance of the comparisons. Cluster-randomized and cross-over trials were also excluded because their special design would easily violate the transitivity principle in the network meta-analysis. Finally, to minimize heterogeneity within the comparator group and to avoid substantial overlap between intervention and control arms, studies in which more than 30% of participants were receiving mineralocorticoid receptor antagonists (MRAs) as part of their background therapy were also excluded.

In duplicate, 2 reviewers (A.L. and A.P.V.) independently screened the references identified by the search and selected eligible trials. Any discrepancies were double-checked and resolved by discussion with another member of the research team (RP).

### Outcomes

The prespecified primary outcome was the change in office SBP from baseline. Additional outcomes of interest were changes from baseline in office diastolic blood pressure (DBP), 24-hour SBP, 24-hour DBP, daytime SBP, daytime DBP, nighttime SBP, and nighttime DBP. Each outcome was analyzed at the time of assessment of the trial’s primary endpoint. Given that each outcome was objective and continuous, we used mean differences as the measure to summarize study results.

### Data analysis

Primarily, we performed random-effects network meta-analyses within a Bayesian framework. Analyses were conducted separately in patients with resistant hypertension and in the broader population of patients with uncontrolled hypertension. Effect size measures were reported along with their 95% credible intervals (CrI). We evaluated the transitivity assumption by comparing the distribution of study characteristics across studies grouped by comparison. Heterogeneity was estimated using a common τ as recommended in the Cochrane Handbook for Systematic Reviews of Interventions.^11^ Global and local approaches were used to evaluate the inconsistency between direct and indirect sources of evidence. We evaluated local inconsistency by separating indirect from direct evidence test, for each comparison (a *P* < 0.10 for a difference between direct and indirect evidence was chosen as the threshold indicative of an inconsistent comparison),^12^ whereas a design-by treatment test was used to evaluate global inconsistency.^13^ When evidence of inconsistency was found, we reported only frequentist pairwise meta-analyses using a random-effects inverse-variance model. Potential sources of heterogeneity and inconsistency in the primary outcome were investigated by using sensitivity and network meta-regression analyses. We performed sensitivity analyses by excluding studies at high risk of bias, by selecting trials of patients using placebo or a sham procedure as the comparator, and by excluding studies in which MRAs were used in the baseline treatment. In Bayesian network meta-regressions, we evaluated the effect of mean age, proportion of female patients, baseline SBP, year of study publication, study duration, and body mass index as potential treatment-effect modifiers. We investigated the presence of small-study effects for each treatment pair (which could be associated with publication bias) using a contour-enhanced funnel plot if at least 10 studies contributed to the analysis.^14^ These analyses were performed only in the population of patients with uncontrolled hypertension, as this represented the largest network and encompassed the subgroup of patients with resistant hypertension. The risk of bias of individual studies was assessed on the primary outcome (for feasibility reasons) using the Cochrane’s Risk of Bias 2 tool.^15^ Then, the overall rating for each study was included in the judgment of confidence in the estimates by using the Confidence In Network Meta-Analysis (CINeMA) approach.^16^ We presented the findings of the network meta-analysis using forest plots and league tables. Statistical analyses were performed with R version 4.4.2 using netmeta and self-programmed routines in rjags (https://github.com/esm-ispm-unibe-ch-REPRODUCIBLE) for the Bayesian analyses. Further details on data analysis are provided in the Supplemental Appendix.

The review protocol is registered with PROSPERO, number CRD420251118562 (Supplemental Appendix).

## Results

We identified 3,074 references through our search (Figure 1). After title and abstract screening, we assessed for eligibility 248 full-text articles, of which 173 were excluded for various reasons (incorrect study design, incorrect population, incorrect background therapy, no randomization, or information too sparse for study inclusion). Finally, 75 references on 35 trials published between 2007 and 2025, comprising 8,648 patients and 12 individual pharmacologic and device-based interventions, were included in the systematic review.

**Figure 1:**
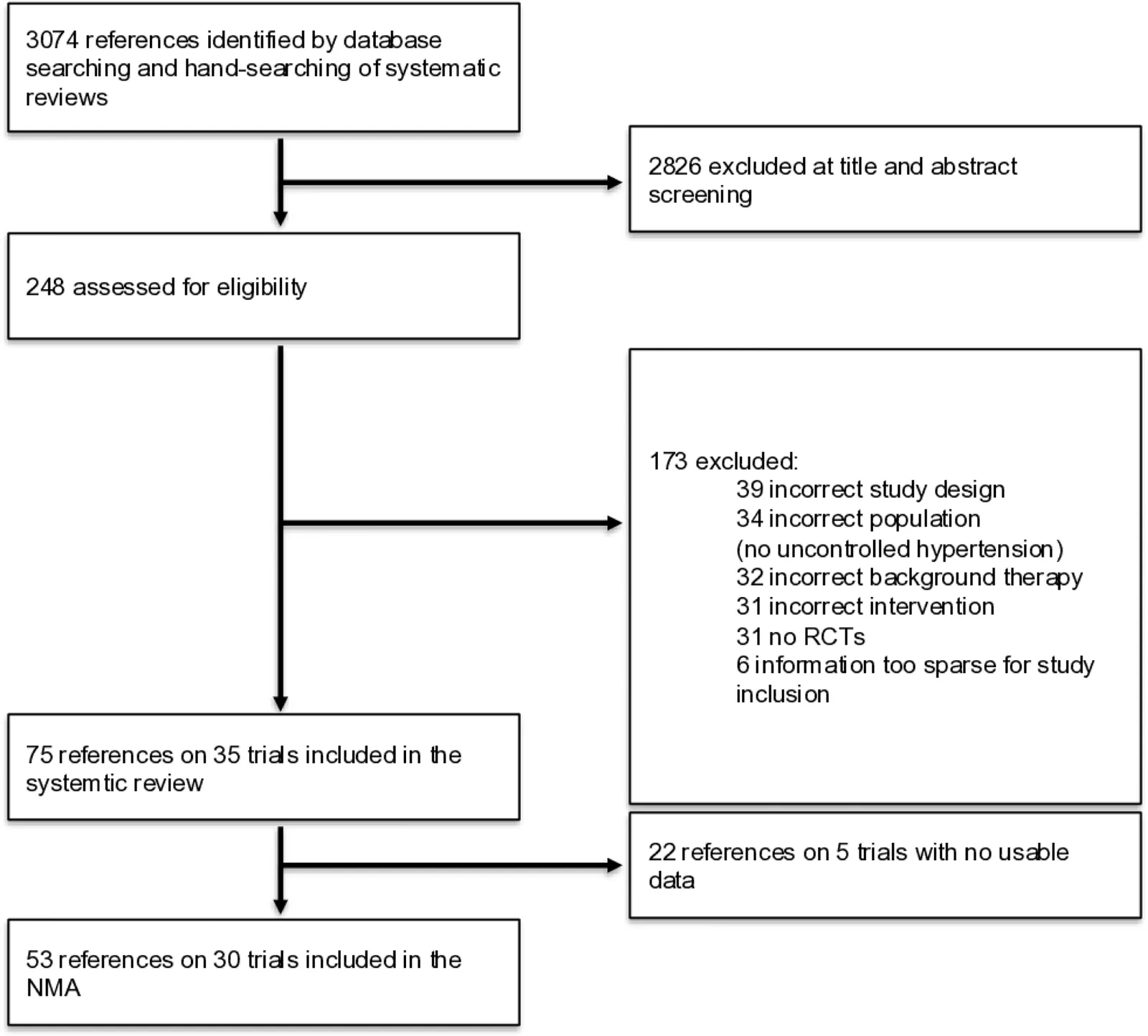
Study Selection NMA = network meta-analysis; RCT = randomized controlled trial.

Baseline characteristics are detailed in the Supplemental Appendix. The median age of participants was 60 years (Q1-Q3: 55-62), the proportion of female patients was 39% (Q1-Q3: 30-46), and the median study duration was 12 weeks (9-26). Ethnicity data were collected but infrequently reported. Median baseline SBP and DBP were 155 mm Hg (149-163) and 90 mm Hg (86-97), respectively. The median body mass index was 31 kg/m^2^ (30-32), and the median estimated glomerular filtration rate was 82 mL/min/1.73 m^2^ (77-86). Of the 35 studies included in the systematic review, 24 (69%) enrolled patients with resistant hypertension, while the remaining 11 (31%) included patients with uncontrolled hypertension despite treatment with at least 2 antihypertensive drugs. No included trials exclusively enrolled patients receiving 4 or more medications. Information on background medication use was collected; however, reporting was inconsistent, and definitions and categorization varied substantially across studies (Supplemental Appendix).

In the network meta-analysis, we included 30 trials for a total of 8,187 patients and 10 individual pharmacologic and device-based interventions, which were divided into the following classes: α-agonists (clonidine, guanfacine), aldosterone synthase inhibitors (baxdrostat, lorundrostat), endothelin receptor antagonists (aprocitentan, darusentan), MRAs (spironolactone), and renal denervation (RDN; alcohol-RDN, radiofrequency-RDN, ultrasound-RDN). We found no clear evidence of violations in the transitivity assumption when comparing characteristics of studies across comparisons. However, for most outcomes, the number of studies per comparison was small, limiting the assessment of transitivity.

### Resistant hypertension

Eighteen trials including 3,794 participants and 9 individual treatments contributed to the network meta-analysis of office SBP (the full set of display items for this analysis is provided in Supplemental Appendix). Spironolactone (mean difference: −12.43 mm Hg; 95% CrI: −17.68 to −7.18; CINeMA moderate) and radiofrequency-RDN (mean difference: −8.74 mm Hg; 95% CrI: −12.96 to −4.52; CINeMA moderate) were more effective than baseline treatment in reducing office SBP, with 95% CrI excluding no effect (Figures 2A and 3A). Among treatments with a probability of no effect, aprocitentan, baxdrostat, and darusentan outperformed baseline treatment in pairwise meta-analyses. In head-to-head comparisons, spironolactone demonstrated greater efficacy than guanfacine and ultrasound-RDN for office SBP reduction. The remaining direct comparisons included a probability of no effect; therefore, there was no clear evidence for the superiority of a specific individual treatment. Similar findings were observed when interventions were grouped into treatment classes (Figure 4A).

**Figure 2:**
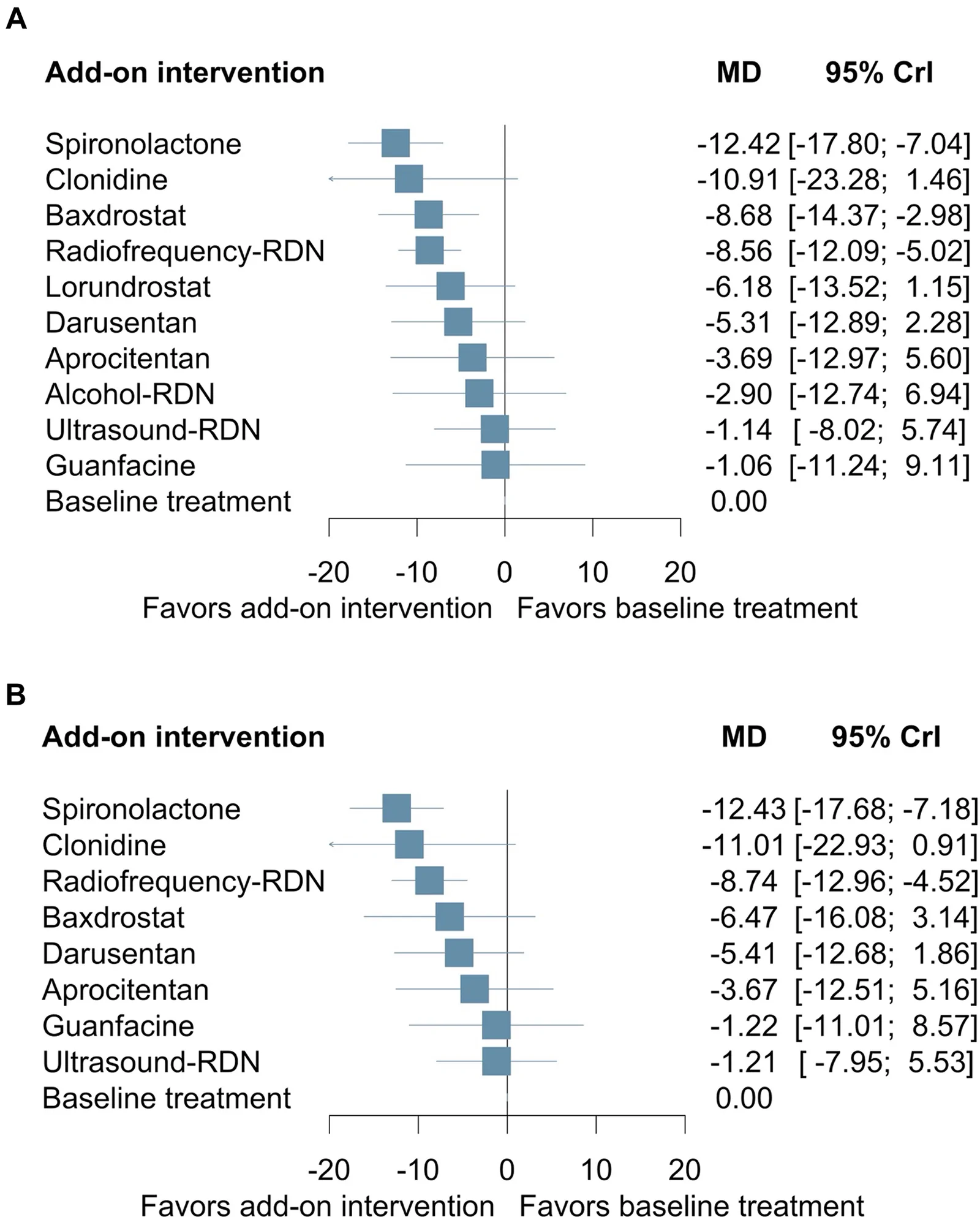
Forest Plot of Systolic Blood Pressure (Individual Treatments) A forest plot showing the effects of individual add-on pharmacologic and device-based interventions compared with baseline treatment on systolic blood pressure, the primary outcome, in patients with resistant (A) and uncontrolled (B) hypertension. Effect sizes are from the network meta-analysis. Treatments are ordered according to the mean difference. The reference is baseline treatment. CrI = credible intervals; MD = mean difference; RDN = renal denervation.

**Figure 4:**
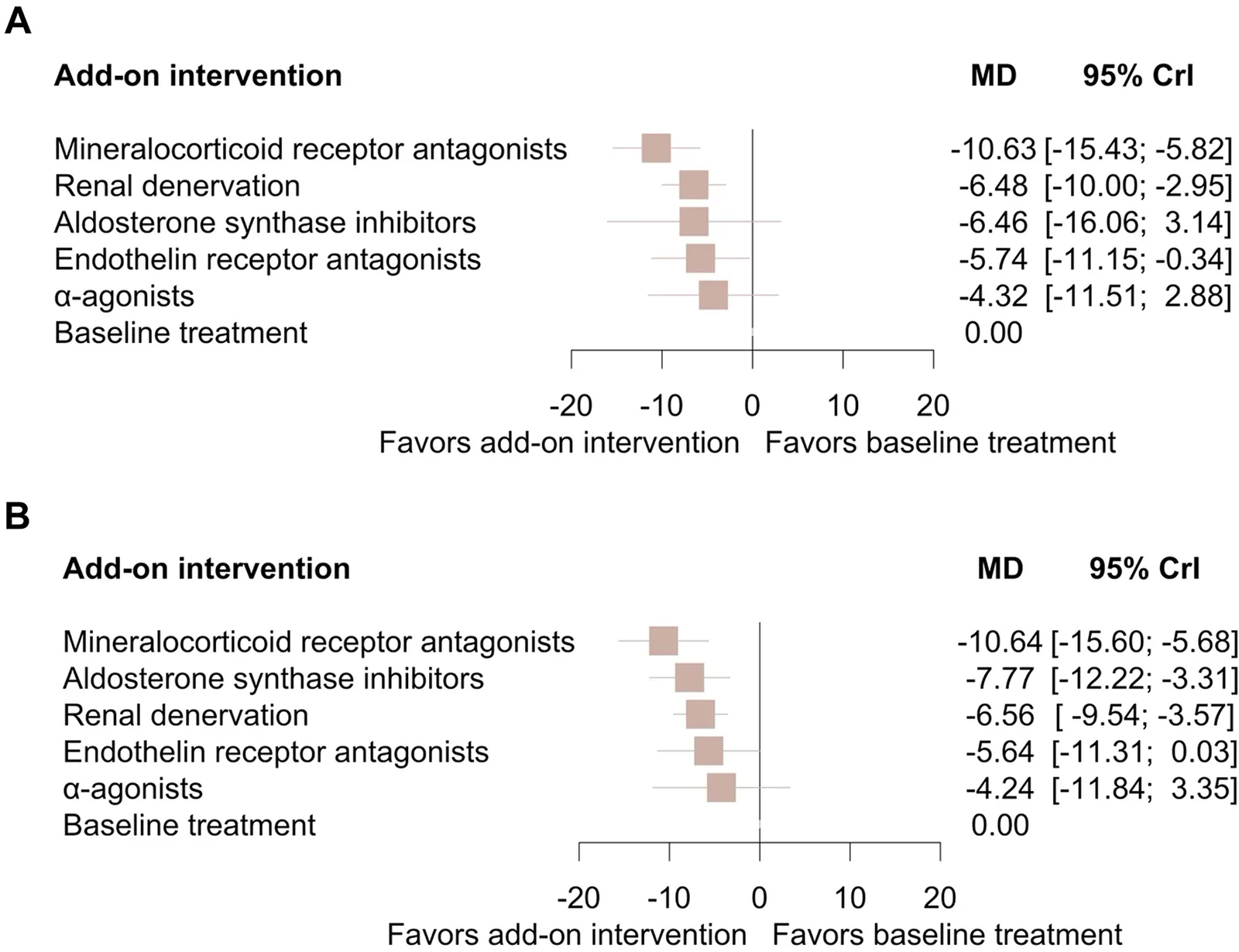
League Table of Systolic Blood Pressure (Individual Treatments) League table showing the effects of individual add-on pharmacologic and device-based interventions compared with baseline treatment on systolic blood pressure, the primary outcome, in patients with resistant (A) and uncontrolled (B) hypertension. Treatments are in alphabetical order. In the upper right triangle are results from the network meta-analysis. In the lower left triangle are results from pairwise meta-analyses (direct comparisons). Each cell provides mean difference and the corresponding 95% credible interval (or CI for pairwise meta-analyses). Bold print indicates 95% credible intervals excluding no effect. For the results of the network meta-analysis, the color of the cells represents the confidence in estimate results (obtained with Confidence In Network Meta-Analysis), with blue indicating moderate confidence, orange indicating low confidence, and red indicating very low confidence. NA = not available; RDN = renal denervation.

**Central Illustration:**
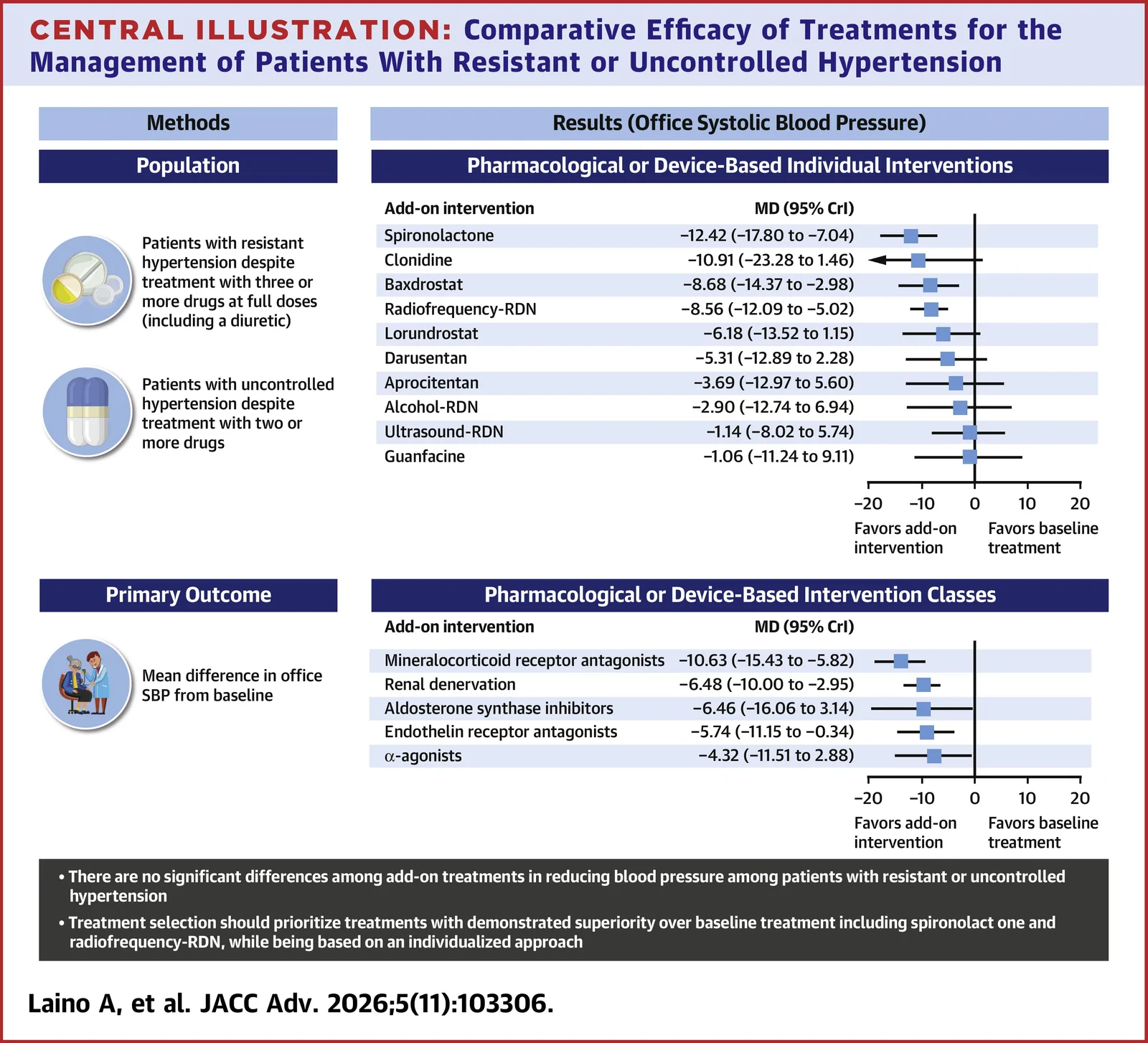
Comparative Efficacy of Treatments for the Management of Patients With Resistant or Uncontrolled Hypertension CrI = credible intervals; MD = mean difference; RDN = renal denervation; SBP = systolic blood pressure.

**Figure 3:**
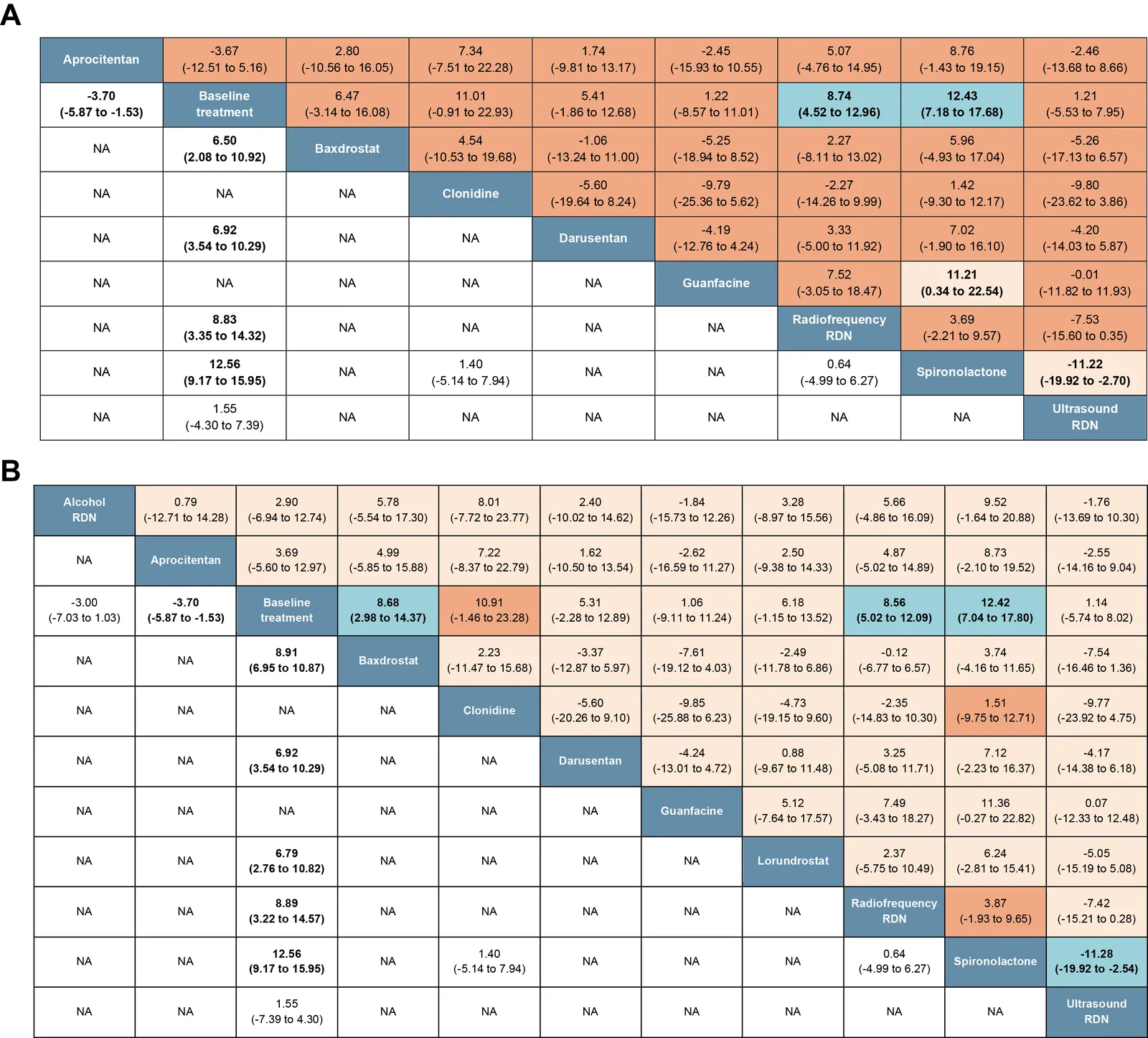
Forest Plot of Systolic Blood Pressure (Treatment Classes) A forest plot showing the effects of add-on pharmacologic and device-based interventions grouped by classes, compared with baseline treatment on systolic blood pressure, the primary outcome, in patients with resistant (A) and uncontrolled (B) hypertension. Effect sizes are from the network meta-analysis. Treatments are ordered according to the mean difference. The reference is the baseline treatment employed in each included trial. Abbreviations as in Figure 2.

For 24-hour SBP, we included 18 studies encompassing 2,815 participants and 8 individual treatments (the full set of display items for this analysis is provided in Supplemental Appendix). Baxdrostat (mean difference: −14.01 mm Hg; 95% CrI: −20.58 to −7.43), spironolactone (mean difference: −9.66 mm Hg; 95% CrI: −13.46 to −5.86), darusentan (mean difference: −6.99 mm Hg; 95% CrI: −13.32 to −0.67), and radiofrequency-RDN (mean difference: −4.41 mm Hg; 95% CrI: −7.08 to −1.74) were superior in reducing 24-hour SBP in comparison to baseline treatment, with 95% CrI excluding no effect. In head-to-head comparisons, baxdrostat achieved greater reductions in 24-hour SBP than guanfacine, radiofrequency-RDN, and ultrasound-RDN. Furthermore, spironolactone was more effective than guanfacine, radiofrequency-RDN, and ultrasound-RDN in reducing 24-hour SBP. The remaining comparisons included the null effect within their CrIs, providing no clear evidence of superiority for any other specific individual intervention.

The results for office DBP, 24-hour DBP, daytime SBP, daytime DBP, nighttime SBP, and nighttime DBP were consistent with those of the primary analysis, confirming the favorable effects of spironolactone and radiofrequency-RDN as individual add-on interventions to baseline treatment. No clear evidence of superiority was observed for any specific pharmacologic and device-based intervention in head-to-head comparisons. Nevertheless, the available evidence was limited for several outcomes, and the resulting estimates should be interpreted cautiously (Supplemental Appendix).

### Uncontrolled hypertension

Twenty-six trials including 7,207 participants and 11 individual treatments contributed to the network meta-analysis of office SBP (the full set of display items for this analysis is provided in Supplemental Appendix). Spironolactone (mean difference: −12.42 mm Hg; 95% CrI: −17.80 to −7.04; CINeMA moderate), baxdrostat (mean difference: −8.68 mm Hg; 95% CrI: −14.37 to −2.98; CINeMA moderate), and radiofrequency-RDN (mean difference: −8.56 mm Hg; 95% CrI: −12.09 to −5.02; CINeMA moderate) were more effective than baseline treatment in reducing office SBP, with 95% CrI excluding no effect (Figures 2B and 3B). Among treatments with a probability of no effect, aprocitentan, darusentan, and lorundrostat outperformed baseline treatment in pairwise meta-analyses. In head-to-head comparisons, spironolactone demonstrated greater efficacy than ultrasound-RDN for office SBP reduction. The remaining direct comparisons included a probability of no effect; therefore, there was no clear evidence for the superiority of a specific individual treatment. These analyses were reproduced consistently when individual pharmacologic and device-based interventions were grouped according to classes (Figure 4B).

In the network meta-analysis of office DBP, we included 23 trials comprising 5,624 patients and 11 individual treatments (the full set of display items for this analysis is provided in Supplemental Appendix). While spironolactone (mean difference: −6.31 mm Hg; 95% CrI: −10.54 to −2.08) and radiofrequency-RDN (mean difference: −4.58 mm Hg; 95% CrI: −7.30 to −1.85) maintained superiority over baseline treatment in terms of office DBP, baxdrostat (mean difference: −4.27 mm Hg; 95% CrI: −9.72 to 1.17) showed a 95% CrI including the probability of no effect. In head-to-head comparisons, there was no evidence of superiority for any specific treatment because most comparisons included the probability of no effect (Central Illustration).

For 24-hour SBP, we included 24 studies with 4,366 participants and 10 individual treatments (the full set of display items for this analysis is provided in Supplemental Appendix). Baxdrostat (mean difference: −14.60 mm Hg; 95% CrI: −19.92 to −9.28), spironolactone (mean difference: −9.62 mm Hg; 95% CrI: −13.44 to −5.81), lorundrostat (mean difference: −7.23 mm Hg; 95% CrI: −14.42 to −0.03), darusentan (mean difference: −7.01 mm Hg; 95% CrI: −13.56 to −0.46), and radiofrequency-RDN (mean difference: −4.22 mm Hg; 95% CrI: −6.54 to −1.89) were superior in reducing 24-hour SBP in comparison to baseline treatment, with 95% CrI excluding no effect. In terms of head-to-head comparisons, baxdrostat achieved greater reductions in 24-hour SBP than alcohol-RDN, guanfacine, radiofrequency-RDN, and ultrasound-RDN. Furthermore, spironolactone showed a greater reduction in 24-hour SBP than guanfacine, radiofrequency-RDN, and ultrasound-RDN. The remaining comparisons included the null effect within their CrIs, indicating no clear evidence of differences between individual interventions.

The results for 24-hour DBP, daytime SBP, daytime DBP, nighttime SBP, and nighttime DBP were similar to those of the main analyses, showing the superiority of spironolactone, baxdrostat, and radiofrequency-RDN in reducing blood pressure when compared with baseline treatment without any evidence of superiority for a specific treatment in head-to-head comparisons. However, data were scarce, and many treatments were not evaluated for each outcome (Supplemental Appendix).

### Heterogeneity

There was high heterogeneity across outcomes (common τ ranged from 1.91 to 4.59) (Supplemental Appendix). We found no substantial evidence of inconsistency in direct and indirect estimates for each outcome evaluated (Supplemental Appendix). There was no evidence of publication bias by visual inspection of the contour-enhanced funnel plot (Supplemental Appendix). The overall risk of bias was judged as low in 8 of 30 studies (27%), with some concerns in 13 (43%), and high in 9 studies (30%) (Supplemental Appendix).

The network meta-regression analyses for office SBP (Supplemental Appendix) showed that the results of the main analysis were largely consistent after adjusting for potential effect modifiers including mean age, proportion of female patients, baseline SBP, year of study publication, and body mass index. In addition, the results of our sensitivity analyses (Supplemental Appendix) conducted by including only placebo/sham studies and excluding studies with high risk of bias or studies using MRAs within baseline treatment were comparable to the results of the primary analysis.

## Discussion

To the best of our knowledge, this systematic review and network meta-analysis represents the most comprehensive data synthesis on add-on treatments for patients with resistant or uncontrolled hypertension. Prior network meta-analyses, focusing on resistant hypertension, failed to account for heterogeneity in background therapies across trials and did not include more recently available treatments, such as baxdrostat and lorundrostat, thereby limiting their clinical applicability.^17^, ^18^, ^19^

The results of our network meta-analysis yielded the following principal findings. First, compared with baseline treatment alone, the addition of spironolactone, baxdrostat, or radiofrequency-RDN consistently reduced the primary outcome of office SBP, as well as multiple other blood pressure measures, across several network meta-regression and subgroup analyses in patients with resistant or uncontrolled hypertension. Second, findings for radiofrequency-RDN and baxdrostat were not entirely generalizable to other interventions within the relative therapeutic class, namely alcohol-RDN or ultrasound-RDN and lorundrostat, respectively. Finally, no clear evidence of superiority emerged because most head-to-head comparisons included probability of no effect.

The most recent European Society of Cardiology and American College of Cardiology (ACC)/American Heart Association (AHA) guidelines recommend a stepwise approach to the management of resistant or uncontrolled hypertension among patients already on 2 antihypertensive drugs.^3^^,^^4^ Specifically, guideline-directed algorithms for uncontrolled hypertension entail the use of a 3-drugs combination therapy, including a thiazide-like diuretic. In case of resistant hypertension, spironolactone is consistently recommended as the preferred fourth-line agent.^3^^,^^4^ In case of intolerance or inefficacy to MRAs, several pharmacologic alternatives are recommended, including eplerenone, α-agonists, α-blockers, amiloride, β-blockers, endothelin receptor antagonists (recommended only by ACC/AHA guidelines), or hydralazine.^3^^,^^4^ Finally, as a last-line option, catheter-based RDN may be considered if hypertension remains uncontrolled despite optimized pharmacologic therapy.^3^^,^^4^

Primary aldosterone dysregulation contributes to the pathogenesis of uncontrolled hypertension in approximately 60% of cases, acting through several mechanisms including sodium retention, volume overload, and vascular remodelling.^20^^,^^21^ Notably, this condition is associated with an increased risk of cardiovascular events, exceeding the risk attributable to hypertension itself.^22^ These concepts have been corroborated by a pivotal randomized trial demonstrating that spironolactone, compared with placebo, reduced office SBP by 10.20 mm Hg in 335 patients with resistant hypertension.^23^ Subsequently, multiple pairwise and network meta-analyses have established the central role of spironolactone for the management of patients with resistant hypertension.^19^^,^^24^^,^^25^ Our analysis aligns with prior reports^19^^,^^24^ by showing that the spironolactone-related reduction of office SBP in cases of resistant or uncontrolled hypertension attests to around 12 mm Hg, surpassing the 5 to 10 mm Hg threshold typically associated with a reduced risk of cardiovascular disease and death.^26^ However, the present study extended these findings, demonstrating that spironolactone significantly reduced ambulatory blood pressure metrics (24-hour SBP by 9.70 mm Hg, daytime SBP by 9.75 mm Hg, and nighttime SBP by 10.99 mm Hg), which are known to have a stronger association with the risk of death than office SBP.^27^ Of note, we were unable to find any randomized trial evaluating eplerenone in patients with resistant or uncontrolled hypertension. In view of the pharmacokinetic and pharmacodynamic differences between these 2 agents,^28^^,^^29^ extrapolating efficacy data of spironolactone to other MRAs, including eplerenone, as currently supported by clinical guidelines, should be approached with caution.

Catheter-based RDN, which modulates efferent and afferent sympathetic renal nerve activity, has emerged over the past years as an invasive strategy to control blood pressure in patients with resistant or uncontrolled hypertension.^30^ In a pairwise meta-analysis enrolling 2,478 patients with hypertension, radiofrequency-RDN reduced both office SBP and DBP by 7.86 mm Hg and 4.55 mm Hg.^8^ Our findings importantly extend this evidence by showing that radiofrequency-RDN reduced both office and ambulatory blood pressure measures in patients with resistant or uncontrolled hypertension, with a magnitude comparable to that observed in this pivotal meta-analysis.^8^ Notably, the results of our study differ from those of a prior network meta-analysis comparing different treatments for patients with resistant hypertension, which showed that radiofrequency-RDN did not reduce both office and 24-hour SBP.^19^ The inclusion of patients with uncontrolled hypertension, the higher number of trials, and the larger total sample for both outcomes in our analyses may be the basis of such different results. Our findings diverged from those of earlier studies regarding the efficacy of alcohol-RDN and ultrasound-RDN, showing that both techniques had 95% CrIs including no effect when compared with baseline treatment across all outcomes.^8^^,^^31^ For alcohol-RDN, this discrepancy likely reflects the exclusion of the TARGET BP OFF (Treatment in Patients with Uncontrolled Hypertension on No Antihypertensive Medication) trial, which enrolled patients not receiving background antihypertensive therapy and reported positive results for this approach vs baseline treatment.^32^ Similarly, prior studies of ultrasound-RDN included patients without background therapies, notably, the RADIANCE-HTN SOLO (A Study of the ReCor Medical Paradise System in Clinical Hypertension) and RADIANCE II (A Study of the Recor Medical Paradise System in Stage II Hypertension) trials, which reported favorable results.^33^^,^^34^ Although these studies may be useful as proof of concept, the evidence accumulated in the context of dedicated trials on uncontrolled hypertension does not support the interchangeability of different RDN techniques as currently implied in guidelines and consensus documents.^3^^,^^4^^,^^35^ Indeed, radiofrequency-RDN emerged as the sole RDN modality currently supported by a moderate level of evidence for the treatment of patients with resistant or uncontrolled hypertension. In this regard, it should be noted that RDN remained a class 2b recommendation in recent guidelines, whereas fourth-line drugs received a class 2a recommendation despite the lack of dedicated randomized trials.

Aldosterone synthase inhibitors, including baxdrostat and lorundrostat, recently emerged as a potential therapeutic option for the management of adult patients with resistant or uncontrolled hypertension.^36^ This class of drug acts by direct inhibition of the aldosterone synthase, which catalyses the final 3 steps in aldosterone biosynthesis, potentially limiting the side effects associated with the use of MRAs.^37^, ^38^, ^39^ In a pairwise meta-analysis including 1,440 hypertensive patients, this class of drug reduced office SBP by 6.3 mm Hg and office DBP by 2.2 mm Hg.^9^ Our analysis confirms and extends these findings by showing that baxdrostat reduced office SBP by 8.68 mm Hg and 24-hour SBP by 14.60 mm Hg in patients with resistant or uncontrolled hypertension. Notably, the findings for office SBP were not fully reproduced in the subgroup restricted to patients with resistant hypertension. However, this analysis was based on a substantially smaller evidence base, resulting in less precise effect estimates and a network considerably smaller than that available for the broader uncontrolled hypertension population. Accordingly, the observed differences between analyses should be interpreted in light of differences in sample size and statistical precision rather than as evidence of true differences in treatment efficacy across populations. While lorundrostat significantly reduced 24-hour SBP by 7.23 mm Hg, we did not find a significant reduction in office SBP given that the upper 95% CrI included no effect. However, point estimates were comparable to those of baxdrostat showing a reduction of office SBP by 6.18 mm Hg. These results were mainly driven by the presence of the Target-HTN (Trial on the Safety and Efficacy of MLS-101 (Lorundrostat) in Patients With Uncontrolled Hypertension), which explored various doses of lorundrostat.^40^ As prespecified in our protocol, to improve data availability, when multiple doses of a drug were reported, we calculated a single weighted mean and standard deviation. Therefore, it is possible that the positive results associated with the 50-mg dose found in this trial were diluted by pooling the negative results observed for the lower doses (25 mg and 12.5 mg). In line with this interpretation, other trials using 50-mg or 100-mg doses of lorundrostat produced results consistent with those of baxdrostat-based trials. Overall, our findings suggest that baxdrostat may represent a treatment option for patients with resistant or uncontrolled hypertension and that lorundrostat may represent a promising strategy that warrants further evidence before clinical implementation.

### Study limitations

The results of the present work should be interpreted considering some limitations. First, we did not have access to individual patient data, which would have allowed a more detailed assessment of potential differential effects of the available treatments across patient subgroups; therefore, our findings should be interpreted as average treatment effects. Nevertheless, multiple network meta-regressions and sensitivity analyses were conducted to explore potential treatment-effect modifiers and to identify clinical settings that may derive greater benefit from specific treatments.

Second, for many outcomes of interest, several interventions were supported by only a limited number of trials and were connected to the network without closed loops. Such thin and open networks provide limited statistical power to assess the assumptions of a network meta-analysis. This aspect may have contributed to the lack of benefit observed for many interventions compared with baseline treatment; therefore, caution is warranted when interpreting our results. To address this limitation, we prespecified a sensitivity analysis in which individual interventions were grouped into broader treatment classes. These analyses generally showed better-connected networks on visual inspection and yielded results that were largely consistent with those of the individual treatments-based analysis. In this respect, the added value of our analysis compared with prior pairwise meta-analyses is preserved. Furthermore, our findings highlight the urgent need for additional head-to-head comparative studies to determine the optimal treatment selection for patients with resistant hypertension.

Third, background therapies varied widely and were poorly described across trials, limiting formal assessment of this important moderator. This issue is common to meta-analyses evaluating add-on treatments and has been described previously.^41^ To minimize this source of heterogeneity, we used concise inclusion criteria as prespecified in our protocol. Accordingly, the present network meta-analysis should be interpreted as informative for selecting the most effective add-on treatment for patients with uncontrolled hypertension on a generic background of therapies that may include 2 or more of the following drugs: angiotensin-converting enzyme inhibitors, angiotensin receptor blockers, calcium channel blockers, β-blockers, or diuretics. However, it is important to note that these agents are not uncommonly used as third-line therapies in clinical practice. Therefore, this limitation should be considered when interpreting the ability of our analysis to inform the selection of a third-line treatment in patients with uncontrolled hypertension.

Fourth, we observed high heterogeneity for many outcomes of interest. Although we explored some treatment-effect modifiers, no clear explanation emerged. However, the magnitude of heterogeneity was comparable to that reported in prior network meta-analyses of treatments for uncontrolled or resistant hypertension.^17^, ^18^, ^19^

Fifth, we included both patients with uncontrolled hypertension receiving ≥2 antihypertensive medications and patients with resistant hypertension. The former group represents a more heterogeneous clinical phenotype that is less strictly defined in current guidelines. Consequently, extrapolating our findings to this specific subgroup may be more challenging, and our results should be interpreted considering this source of heterogeneity. In addition, each trial included in the resistant hypertension analysis defined this condition as the presence of SBP ≥140 mm Hg despite treatment with 3 or more antihypertensive drugs, including a diuretic, at full doses. Therefore, it was not feasible to evaluate the potential influence of alternative definitions of resistant hypertension on our findings, such as that recently adopted by the AHA/ACC hypertension guidelines.^4^

Sixth, we did not control for drug dose in our analyses. In most included studies, clinicians were allowed to titrate doses for individual participants. Furthermore, to improve data availability, when multiple doses of a drug were evaluated within a trial, we pooled them by calculating a single weighted mean and standard deviation. These factors may have introduced important differences in blood pressure outcomes, potentially limiting the clinical interpretability of our results.

Seventh, we did not perform formal cost-effectiveness or safety analyses. This represents an important limitation, as treatment decisions by patients and clinicians are based not only on efficacy but also on the incidence and severity of adverse effects and considerations of cost-effectiveness.

Eighth, we did not evaluate interventions specifically aimed at improving medication adherence as a separate treatment category. In routine clinical practice, many cases of resistant or uncontrolled hypertension are attributable, at least in part, to poor medication adherence, which may be influenced by several factors, including treatment complexity, concerns regarding adverse effects, negative perceptions of medications, socioeconomic factors, and financial hardship.^42^^,^^43^ Although many of the included trials incorporated a run-in period with a standardized antihypertensive therapy and assessed adherence before randomization, enrolling only participants who demonstrated adequate adherence to treatment, these conditions may not reflect the levels of adherence typically observed in routine clinical practice. Consequently, the treatment effects observed in our analysis may not fully capture the effectiveness of these interventions in real-world settings, where medication nonadherence remains a major contributor to uncontrolled and resistant hypertension.

Ninth, information on ethnicity and genetic background was sparsely reported across included studies. As genetic variant factors may influence blood pressure regulation and treatment response, the extent to which our findings are generalizable across different ethnical groups remains uncertain.^44^ Future trials should report more detailed information on participants' ethnicity to facilitate the evaluation of potential genetic determinants of treatment efficacy and support the development of more personalized treatments.

Finally, data on physical activity were not available for participants included in most trials. Consequently, we could not assess whether differences in physical activity levels contributed to the observed treatment effects or represented a source of residual confounding.

## Conclusions

Our analysis showed that spironolactone, baxdrostat, and radiofrequency-RDN are the treatments supported by a substantial body of evidence that consistently reduced all measures of blood pressure in the short term compared with baseline treatment in patients with resistant or uncontrolled hypertension. Conversely, current evidence for eplerenone, α-agonists, α-blockers, amiloride, and endothelin receptor antagonists is more limited, and their estimated effects may change as additional studies will become available. These results should be interpreted within the limitations of this analysis, specifically the inclusion of a broad patient population, the absence of individual patient-level data to tailor treatment selection, uncertainties regarding optimal dosing, and a lack of robust long-term clinical outcomes and safety data. Nevertheless, because several add-on interventions showed efficacy compared with baseline treatment, our findings provide estimates of average treatment effects and identify a subset of therapeutic options from which clinicians can tailor an individualized antihypertensive therapy based on clinical circumstances, benefit-risk profile, patient preferences, treatment costs, and local resource availability and reimbursement policies, particularly among patients with limited financial resources. Our findings should be considered as a basis for shared decision-making between patients, clinicians, and policymakers. For example, radiofrequency-RDN may be preferred in patients with poor adherence to pharmacologic therapy, whereas lower-cost pharmacologic strategies may be more appropriate when catheter-based interventions or more expensive therapies are unavailable or not reimbursed within a given health care system. However, dedicated studies are required to identify patient-specific characteristics that would enable personalized estimates of the comparative effectiveness of antihypertensive interventions.

Perspectives **COMPETENCY IN PATIENT CARE AND PROCEDURAL SKILLS:** We showed that there are no significant differences among add-on treatments in reducing blood pressure in patients with resistant or uncontrolled hypertension. Treatment selection should prioritize treatments with demonstrated superiority over baseline treatment including spironolactone and radiofrequency-RDN, while being based on an individualized approach.

**TRANSLATIONAL OUTLOOK:** Large, adequately powered randomized trials are needed to establish the most effective add-on pharmacologic or device-based intervention among patients with resistant or uncontrolled hypertension.

## Funding support and author disclosures

Dr Esposito has received personal fees from Abbott Vascular, Amgen, Edwards Lifesciences, and Sanofi, outside the submitted work; and research grants were provided to the institution from Alvimedica and Medtronic. All other authors have reported that they have no relationships relevant to the contents of this paper to disclose.

## References

[1] NCD Risk Factor Collaboration (NCD-RisC) (2021). Worldwide trends in hypertension prevalence and progress in treatment and control from 1990 to 2019: a pooled analysis of 1201 population-representative studies with 104 million participants. Lancet.

[2] Zanchetti A., Thomopoulos C., Parati G. (2015). Randomized controlled trials of blood pressure lowering in hypertension. Circ Res.

[3] McEvoy J.W., McCarthy C.P., Bruno R.M. (2024). 2024 ESC guidelines for the management of elevated blood pressure and hypertension. Eur Heart J.

[4] Jones D.W., Jones D.W., Ferdinand K.C. (2025). 2025 AHA/ACC/AANP/AAPA/ABC/ACCP/ACPM/AGS/AMA/ASPC/NMA/PCNA/SGIM guideline for the prevention, detection, evaluation and management of high blood pressure in adults: a report of the American College of Cardiology/American Heart Association Joint Committee on clinical practice guidelines. J Am Coll Cardiol.

[5] Hutton B., Salanti G., Caldwell D.M. (2015). The PRISMA extension statement for reporting of systematic reviews incorporating network meta-analyses of health care interventions: checklist and explanations. Ann Intern Med.

[6] Lemeshow A.R., Blum R.E., Berlin J.A., Stoto M.A., Colditz G.A. (2005). Searching one or two databases was insufficient for meta-analysis of observational studies. J Clin Epidemiol.

[7] Mahfooz K., Najeed S., Tun H.N. (2023). New dual endothelin receptor antagonist aprocitentan in hypertension: a systematic review and meta-analysis. Curr Probl Cardiol.

[8] Vukadinović D., Lauder L., Kandzari D.E. (2024). Effects of catheter-based renal denervation in hypertension: a systematic review and meta-analysis. Circulation.

[9] Marzano L., Merlo M., Martinelli N., Pizzolo F., Friso S. (2025). Efficacy and safety of aldosterone synthase inhibitors for hypertension: a meta-analysis of randomized controlled trials and systematic review. Hypertension.

[10] Kobayashi M., Pitt B., Ferreira J.P., Rossignol P., Girerd N., Zannad F. (2025). Aldosterone-targeted therapies: early implementation in resistant hypertension and chronic kidney disease. Eur Heart J.

[11] Chaimani A., Caldwell D.M., Li T., Higgins J.P., Salanti G., Higgins J.P., Thomas J., Chandler J., Cumpston M., Li T., Page M.J. (2024). Cochrane Handbook for Systematic Reviews of Interventions.

[12] Dias S., Welton N.J., Caldwell D.M., Ades A.E. (2010). Checking consistency in mixed treatment comparison meta-analysis. Stat Med.

[13] Higgins J.P.T., Jackson D., Barrett J.K., Lu G., Ades A.E., White I.R. (2012). Consistency and inconsistency in network meta-analysis: concepts and models for multi-arm studies. Res Synth Methods.

[14] Peters J.L., Sutton A.J., Jones D.R., Abrams K.R., Rushton L. (2008). Contour-enhanced meta-analysis funnel plots help distinguish publication bias from other causes of asymmetry. J Clin Epidemiol.

[15] Sterne J.A.C., Savović J., Page M.J. (2019). RoB 2: a revised tool for assessing risk of bias in randomised trials. BMJ.

[16] Nikolakopoulou A., Higgins J.P.T., Papakonstantinou T., Chaimani A., Del Giovane C., Egger M., Salanti G. (2020). CINeMA: an approach for assessing confidence in the results of a network meta-analysis. PLoS Med.

[17] Makai P., IntHout J., Deinum J., Jenniskens K., van der Wilt G.J. (2017). A network meta-analysis of clinical management strategies for treatment-resistant hypertension: making optimal use of the evidence. J Gen Intern Med.

[18] Ahmed M., Nudy M., Bussa R., Filippone E.J., Foy A.J. (2023). A systematic review and meta-analysis of all sham and placebo controlled trials for resistant hypertension. Eur J Intern Med.

[19] Tian Z., Vollmer Barbosa C., Lang H., Bauersachs J., Melk A., Schmidt B.M.W. (2024). Efficacy of pharmacological and interventional treatment for resistant hypertension: a network meta-analysis. Cardiovasc Res.

[20] Schiffrin E.L. (2006). Effects of aldosterone on the vasculature. Hypertension.

[21] Brown J.M., Siddiqui M., Calhoun D.A., Carey R.M., Hopkins P.N., Williams G.H., Vaidya A. (2020). The unrecognized prevalence of primary aldosteronism. Ann Intern Med.

[22] Monticone S., D'Ascenzo F., Moretti C., Williams T.A., Veglio F., Gaita F., Mulatero P. (2018). Cardiovascular events and target organ damage in primary aldosteronism compared with essential hypertension: a systematic review and meta-analysis. Lancet Diabetes Endocrinol.

[23] Williams B., MacDonald T.M., Morant S. (2015). Spironolactone versus placebo, bisoprolol, and doxazosin to determine the optimal treatment for drug-resistant hypertension (PATHWAY-2): a randomised, double-blind, crossover trial. Lancet.

[24] Zhao D., Liu H., Dong P., Zhao J. (2017). A meta-analysis of add-on use of spironolactone in patients with resistant hypertension. Int J Cardiol.

[25] Bazoukis G., Thomopoulos C., Tsioufis C. (2018). Effect of mineralocorticoid antagonists on blood pressure lowering. J Hypertens.

[26] Ettehad D., Emdin C.A., Kiran A. (2016). Blood pressure lowering for prevention of cardiovascular disease and death: a systematic review and meta-analysis. Lancet.

[27] Staplin N., de la Sierra A., Ruilope L.M. (2023). Relationship between clinic and ambulatory blood pressure and mortality: an observational cohort study in 59 124 patients. Lancet.

[28] Struthers A., Krum H., Williams G.H.A. (2008). Comparison of the aldosterone-blocking agents eplerenone and spironolactone. Clin Cardiol.

[29] Agarwal R., Kolkhof P., Bakris G., Bauersachs J., Haller H., Wada T., Zannad F. (2021). Steroidal and non-steroidal mineralocorticoid receptor antagonists in cardiorenal medicine. Eur Heart J.

[30] DiBona G.F. (2005). Physiology in perspective: the Wisdom of the body. Neural control of the kidney. Am J Physiol Regul Integr Comp Physiol.

[31] Azizi M., Sharp A.S., Fisher N.D. (2024). Patient-level pooled analysis of endovascular ultrasound renal denervation or a sham procedure 6 months after medication escalation: the RADIANCE clinical trial program. Circulation.

[32] Pathak A., Rudolph U.M., Saxena M. (2023). Alcohol-mediated renal denervation in patients with hypertension in the absence of antihypertensive medications. EuroIntervention.

[33] Azizi M., Schmieder R.E., Mahfoud F. (2018). Endovascular ultrasound renal denervation to treat hypertension (RADIANCE-HTN SOLO): a multicentre, international, single-blind, randomised, sham-controlled trial. Lancet.

[34] Azizi M., Saxena M., Wang Y. (2023). Endovascular ultrasound renal denervation to treat hypertension. JAMA.

[35] Barbato E., Azizi M., Schmieder R.E. (2023). Renal denervation in the management of hypertension in adults. A clinical consensus statement of the ESC council on hypertension and the European Association of Percutaneous Cardiovascular Interventions (EAPCI). Eur Heart J.

[36] Guzik T.J., Tomaszewski M. (2025). Aldosterone synthase inhibition for hypertension. N Engl J Med.

[37] Azizi M., Sanghvi K., Saxena M. (2021). Ultrasound renal denervation for hypertension resistant to a triple medication pill (RADIANCE-HTN TRIO): a randomised, multicentre, single-blind, sham-controlled trial. Lancet.

[38] Azizi M. (2023). Decreasing the effects of aldosterone. in resistant hypertension — a success story. N Engl J Med.

[39] Pyne L., Rossignol P., Giles C. (2025). Safety and efficacy of steroidal mineralocorticoid receptor antagonists in patients with kidney failure requiring dialysis: a systematic review and meta-analysis of randomised controlled trials. Lancet.

[40] Laffin L.J., Rodman D., Luther J.M. (2023). Aldosterone synthase inhibition with lorundrostat for uncontrolled hypertension. JAMA.

[41] Salahuddin N.H., Schütz A., Pitschel-Walz G. (2024). Psychological and psychosocial interventions for treatment-resistant schizophrenia: a systematic review and network meta-analysis. Lancet Psychiatry.

[42] Kronish I.M., Thorpe C.T., Voils C.I. (2021). Measuring the multiple domains of medication nonadherence: findings from a Delphi survey of adherence experts. Transl Behav Med.

[43] Burnier M., Azizi M., Magne J. (2025). Patient perceptions, motivations and barriers to treatment adherence in hypertension: results of a questionnaire-based survey in five European countries. Blood Press.

[44] Pandey K.N. (2024). Genetic and epigenetic mechanisms regulating blood pressure and kidney dysfunction. Hypertension.

