# Supplementary material for: Comparative Efficacy of Treatments for Resistant or Uncontrolled Hypertension: A Network Meta-Analysis of Randomized Trials
Source: JACC Adv. 2026 Oct 5;5(11):103306. doi: 10.1016/j.jacadv.2026.103306 (PMC13673046; doi:10.1016/j.jacadv.2026.103306)
Supplement: Supplementary file 1 [file mmc1.docx]

**SUPPLEMENTARY APPENDIX**

**Comparative efficacy of treatments for resistant or uncontrolled hypertension: a network meta-analysis of randomized trials**

**TABLE OF CONTENTS**

[PRISMA checklist	5](#_Toc233216416)

[1.	PROSPERO protocol	9](#_Toc233216417)

[2.	Search Strategy	17](#_Toc233216418)

[2.1	Search strings	17](#_Toc233216419)

[2.1.1	MEDLINE	17](#_Toc233216420)

[2.1.2	Embase	18](#_Toc233216421)

[3.	Eligible interventions	19](#_Toc233216422)

[4.	Details of the data analysis	21](#_Toc233216423)

[**4.1**	**Measure of treatment effect**	21](#_Toc233216424)

[5.	Characteristics of studies included in the analyses	22](#_Toc233216425)

[**5.1**	**Design of the included studies**	23](#_Toc233216426)

[**5.2**	**Baseline characteristics of the included studies**	31](#_Toc233216427)

[**5.3**	**Background antihypertensive drugs used in the included studies**	37](#_Toc233216428)

[**5.4**	**Reason for exclusion from the quantitative synthesis**	44](#_Toc233216429)

[6.	Transitivity assessment	45](#_Toc233216430)

[**6.1**	**Mean age**	46](#_Toc233216431)

[**6.2**	**Proportion of females**	48](#_Toc233216432)

[**6.3**	**Baseline systolic blood pressure**	50](#_Toc233216433)

[**6.4**	**Year of study publication**	52](#_Toc233216434)

[**6.5**	**Study duration**	54](#_Toc233216435)

[7.	Resistant hypertension	56](#_Toc233216436)

[**7.1**	**Office systolic blood pressure**	57](#_Toc233216437)

[7.1.1	Network plot	57](#_Toc233216438)

[7.1.2	Forest-plot of pairwise meta-analysis	59](#_Toc233216439)

[7.1.3	League table	61](#_Toc233216440)

[**7.2**	**Office diastolic blood pressure**	62](#_Toc233216441)

[7.2.1	Network plot	62](#_Toc233216442)

[7.2.2	Forest-plot of network meta-analysis	64](#_Toc233216443)

[7.2.3	Forest-plot of pairwise meta-analysis	66](#_Toc233216444)

[7.2.4	League table	68](#_Toc233216445)

[**7.3**	**24-hour systolic blood pressure**	70](#_Toc233216446)

[7.3.1	Network plot	70](#_Toc233216447)

[7.3.2	Forest-plot of network meta-analysis	72](#_Toc233216448)

[7.3.3	Forest-plot of pairwise meta-analysis	74](#_Toc233216449)

[7.3.4	League table	76](#_Toc233216450)

[**7.4**	**24-hour diastolic blood pressure**	78](#_Toc233216451)

[7.4.1	Network plot	78](#_Toc233216452)

[7.4.2	Forest-plot of network meta-analysis	80](#_Toc233216453)

[7.4.3	Forest-plot of pairwise meta-analysis	82](#_Toc233216454)

[7.4.4	League table	84](#_Toc233216455)

[**7.5**	**Daytime systolic blood pressure**	86](#_Toc233216456)

[7.5.1	Network plot	86](#_Toc233216457)

[7.5.2	Forest-plot of network meta-analysis	88](#_Toc233216458)

[7.5.3	Forest-plot of pairwise meta-analysis	90](#_Toc233216459)

[7.5.4	League table	92](#_Toc233216460)

[**7.6**	**Daytime diastolic blood pressure**	94](#_Toc233216461)

[7.6.1	Network plot	94](#_Toc233216462)

[7.6.2	Forest-plot of network meta-analysis	96](#_Toc233216463)

[7.6.3	Forest-plot of pairwise meta-analysis	98](#_Toc233216464)

[7.6.4	League table	100](#_Toc233216465)

[**7.7**	**Nighttime systolic blood pressure**	102](#_Toc233216466)

[7.7.1	Network plot	102](#_Toc233216467)

[7.7.2	Forest-plot of network meta-analysis	104](#_Toc233216468)

[7.7.3	Forest-plot of pairwise meta-analysis	106](#_Toc233216469)

[7.7.4	League table	108](#_Toc233216470)

[**7.8**	**Nighttime diastolic blood pressure**	110](#_Toc233216471)

[7.8.1	Network plot	110](#_Toc233216472)

[7.8.2	Forest-plot of network meta-analysis	112](#_Toc233216473)

[7.8.3	Forest-plot of pairwise meta-analysis	114](#_Toc233216474)

[7.8.4	League table	116](#_Toc233216475)

[8.	Uncontrolled hypertension	118](#_Toc233216476)

[**8.1**	**Office systolic blood pressure**	119](#_Toc233216477)

[8.1.1	Network plot	119](#_Toc233216478)

[8.1.2	Forest-plot of pairwise meta-analysis	121](#_Toc233216479)

[8.1.3	League table	125](#_Toc233216480)

[8.1.4	Network meta-regression analyses	126](#_Toc233216481)

[8.1.5	Sensitivity analyses	137](#_Toc233216482)

[**8.2**	**Office diastolic blood pressure**	144](#_Toc233216483)

[8.2.1	Network plot	144](#_Toc233216484)

[8.2.2	Forest-plot of network meta-analysis	146](#_Toc233216485)

[8.2.3	Forest-plot of pairwise meta-analysis	148](#_Toc233216486)

[8.2.4	League table	151](#_Toc233216487)

[**8.3**	**24-hour systolic blood pressure**	153](#_Toc233216488)

[8.3.1	Network plot	153](#_Toc233216489)

[8.3.2	Forest-plot of network meta-analysis	155](#_Toc233216490)

[8.3.3	Forest-plot of pairwise meta-analysis	157](#_Toc233216491)

[8.3.4	League table	160](#_Toc233216492)

[**8.4**	**24-hour diastolic blood pressure**	162](#_Toc233216493)

[8.4.1	Network plot	162](#_Toc233216494)

[8.4.2	Forest-plot of network meta-analysis	164](#_Toc233216495)

[8.4.3	Forest-plot of pairwise meta-analysis	166](#_Toc233216496)

[8.4.4	League table	168](#_Toc233216497)

[**8.5**	**Daytime systolic blood pressure**	170](#_Toc233216498)

[8.5.1	Network plot	170](#_Toc233216499)

[8.5.2	Forest-plot of network meta-analysis	172](#_Toc233216500)

[8.5.3	Forest-plot of pairwise meta-analysis	174](#_Toc233216501)

[8.5.4	League table	176](#_Toc233216502)

[**8.6**	**Daytime diastolic blood pressure**	178](#_Toc233216503)

[8.6.1	Network plot	178](#_Toc233216504)

[8.6.2	Forest-plot of network meta-analysis	180](#_Toc233216505)

[8.6.3	Forest-plot of pairwise meta-analysis	182](#_Toc233216506)

[8.6.4	League table	184](#_Toc233216507)

[**8.7**	**Nighttime systolic blood pressure**	186](#_Toc233216508)

[8.7.1	Network plot	186](#_Toc233216509)

[8.7.2	Forest-plot of network meta-analysis	188](#_Toc233216510)

[8.7.3	Forest-plot of pairwise meta-analysis	190](#_Toc233216511)

[8.7.4	League table	192](#_Toc233216512)

[**8.8**	**Nighttime diastolic blood pressure**	194](#_Toc233216513)

[8.8.1	Network plot	194](#_Toc233216514)

[8.8.2	Forest-plot of network meta-analysis	196](#_Toc233216515)

[8.8.3	Forest-plot of pairwise meta-analysis	198](#_Toc233216516)

[8.8.4	League table	200](#_Toc233216517)

[9.	Heterogeneity in the network meta-analysis of primary and secondary outcomes	202](#_Toc233216518)

[10.	Inconsistency in the network meta-analysis of primary and secondary outcomes	204](#_Toc233216519)

[11.	Assessment of small-trial/publication bias	206](#_Toc233216520)

[12.	Assessment of risk of bias	207](#_Toc233216521)

[13.	Assessment of confidence in estimates	209](#_Toc233216522)

[14.	References	212](#_Toc233216523)

# **PRISMA checklist**

PRISMA NMA Checklist of Items to Include When Reporting A Systematic Review Involving a Network Meta-analysis.

| **Section/Topic** | **Item #** | **Checklist Item** | **Reported on Page #** |
| --- | --- | --- | --- |
| **TITLE** |  |  |  |
| Title | 1 | Identify the report as a systematic review *incorporating a network meta-analysis (or related form of meta-analysis).* | 1 |
|  |  |  |  |
| **ABSTRACT** |  |  |  |
| Structured summary | 2 | Provide a structured summary including, as applicable:  **Background:** main objectives  **Methods:** data sources; study eligibility criteria, participants, and interventions; study appraisal; and *synthesis methods, such as network meta-analysis.*  **Results:** number of studies and participants identified; summary estimates with corresponding confidence/credible intervals; *treatment rankings may also be discussed. Authors may choose to summarize pairwise comparisons against a chosen treatment included in their analyses for brevity.*  **Discussion/Conclusions:** limitations; conclusions and implications of findings.  **Other:** primary source of funding; systematic review registration number with registry name. | 2 |
|  |  |  |  |
| **INTRODUCTION** |  |  |  |
| Rationale | 3 | Describe the rationale for the review in the context of what is already known*, including mention of why a network meta-analysis has been conducted.* | 5 |
| Objectives | 4 | Provide an explicit statement of questions being addressed, with reference to participants, interventions, comparisons, outcomes, and study design (PICOS). | 5 |
|  |  |  |  |
| **METHODS** |  |  |  |
| Protocol and registration | 5 | Indicate whether a review protocol exists and if and where it can be accessed (e.g., Web address); and, if available, provide registration information, including registration number. | 8 |
| Eligibility criteria | 6 | Specify study characteristics (e.g., PICOS, length of follow-up) and report characteristics (e.g., years considered, language, publication status) used as criteria for eligibility, giving rationale. *Clearly describe eligible treatments included in the treatment network and note whether any have been clustered or merged into the same node (with justification).* | 7 |
| Information sources | 7 | Describe all information sources (e.g., databases with dates of coverage, contact with study authors to identify additional studies) in the search and date last searched. | 6 |
| Search | 8 | Present full electronic search strategy for at least one database, including any limits used, such that it could be repeated. | 6 |
| Study selection | 9 | State the process for selecting studies (i.e., screening, eligibility, included in systematic review, and, if applicable, included in the meta-analysis). | 6-7 |
| Data collection process | 10 | Describe method of data extraction from reports (e.g., piloted forms, independently, in duplicate) and any processes for obtaining and confirming data from investigators. | 6-7 |
| Data items | 11 | List and define all variables for which data were sought (e.g., PICOS, funding sources) and any assumptions and simplifications made. | 6-7 |
| **Geometry of the network** | **S1** | Describe methods used to explore the geometry of the treatment network under study and potential biases related to it. This should include how the evidence base has been graphically summarized for presentation, and what characteristics were compiled and used to describe the evidence base to readers. | 7-8 |
| Risk of bias within individual studies | 12 | Describe methods used for assessing risk of bias of individual studies (including specification of whether this was done at the study or outcome level), and how this information is to be used in any data synthesis. | 7-8 |
| Summary measures | 13 | State the principal summary measures (e.g., risk ratio, difference in means). *Also describe the use of additional summary measures assessed, such as treatment rankings and surface under the cumulative ranking curve (SUCRA) values, as well as modified approaches used to present summary findings from meta-analyses.* | 7-8 |
| Planned methods of analysis | 14 | Describe the methods of handling data and combining results of studies for each network meta-analysis. This should include, but not be limited to:   - *Handling of multi-arm trials;* - *Selection of variance structure;* - *Selection of prior distributions in Bayesian analyses; and* - *Assessment of model fit.* | 7-8 |
| **Assessment of Inconsistency** | **S2** | Describe the statistical methods used to evaluate the agreement of direct and indirect evidence in the treatment network(s) studied. Describe efforts taken to address its presence when found. | 7-8 |
| Risk of bias across studies | 15 | Specify any assessment of risk of bias that may affect the cumulative evidence (e.g., publication bias, selective reporting within studies). | 7-8 |
| Additional analyses | 16 | Describe methods of additional analyses if done, indicating which were pre-specified. This may include, but not be limited to, the following:   - Sensitivity or subgroup analyses; - Meta-regression analyses; - *Alternative formulations of the treatment network; and* - *Use of alternative prior distributions for Bayesian analyses (if applicable).* | 7-8 |
|  |  |  |  |
| **RESULTS†** |  |  |  |
| Study selection | 17 | Give numbers of studies screened, assessed for eligibility, and included in the review, with reasons for exclusions at each stage, ideally with a flow diagram. | 9 |
| **Presentation of network structure** | **S3** | Provide a network graph of the included studies to enable visualization of the geometry of the treatment network. | 9 |
| **Summary of network geometry** | **S4** | Provide a brief overview of characteristics of the treatment network. This may include commentary on the abundance of trials and randomized patients for the different interventions and pairwise comparisons in the network, gaps of evidence in the treatment network, and potential biases reflected by the network structure. | 9 |
| Study characteristics | 18 | For each study, present characteristics for which data were extracted (e.g., study size, PICOS, follow-up period) and provide the citations. | 9-10 |
| Risk of bias within studies | 19 | Present data on risk of bias of each study and, if available, any outcome level assessment. | 11 |
| Results of individual studies | 20 | For all outcomes considered (benefits or harms), present, for each study: 1) simple summary data for each intervention group, and 2) effect estimates and confidence intervals. *Modified approaches may be needed to deal with information from larger networks.* | 8-11 |
| Synthesis of results | 21 | Present results of each meta-analysis done, including confidence/credible intervals. *In larger networks, authors may focus on comparisons versus a particular comparator (e.g. placebo or standard care), with full findings presented in an appendix. League tables and forest plots may be considered to summarize pairwise comparisons.* If additional summary measures were explored (such as treatment rankings), these should also be presented. | 8-11 |
| **Exploration for inconsistency** | **S5** | Describe results from investigations of inconsistency. This may include such information as measures of model fit to compare consistency and inconsistency models, *P* values from statistical tests, or summary of inconsistency estimates from different parts of the treatment network. | 11-12 |
| Risk of bias across studies | 22 | Present results of any assessment of risk of bias across studies for the evidence base being studied. | 11-12 |
| Results of additional analyses | 23 | Give results of additional analyses, if done (e.g., sensitivity or subgroup analyses, meta-regression analyses*, alternative network geometries studied, alternative choice of prior distributions for Bayesian analyses,* and so forth). | 12 |
|  |  |  |  |
| **DISCUSSION** |  |  |  |
| Summary of evidence | 24 | Summarize the main findings, including the strength of evidence for each main outcome; consider their relevance to key groups (e.g., healthcare providers, users, and policy-makers). | 13 |
| Limitations | 25 | Discuss limitations at study and outcome level (e.g., risk of bias), and at review level (e.g., incomplete retrieval of identified research, reporting bias). *Comment on the validity of the assumptions, such as transitivity and consistency. Comment on any concerns regarding network geometry (e.g., avoidance of certain comparisons).* | 16-18 |
| Conclusions | 26 | Provide a general interpretation of the results in the context of other evidence, and implications for future research. | 19 |
|  |  |  |  |
| **FUNDING** |  |  |  |
| Funding | 27 | Describe sources of funding for the systematic review and other support (e.g., supply of data); role of funders for the systematic review. This should also include information regarding whether funding has been received from manufacturers of treatments in the network and/or whether some of the authors are content experts with professional conflicts of interest that could affect use of treatments in the network. | 8 |

PICOS = population, intervention, comparators, outcomes, study design.

* Text in italics indicates wording specific to reporting of network meta-analyses that has been added to guidance from the PRISMA statement.

† Authors may wish to plan for use of appendices to present all relevant information in full detail for items in this section.

# 1. **PROSPERO protocol**

# 2. **Search Strategy**

The following databases were searched from inception:

1. MEDLINE;

2. Embase.

In addition, we will hand search the reference lists of the included trials and relevant systematic reviews and meta-analyses, editorials and guidelines on treatment of adults with uncontrolled hypertension to identify additional studies missed from the original electronic searches.

## 2.1 Search strings

### 2.1.1 MEDLINE

1. ("hypertension"[Title] OR "uncontrolled hypertension"[Title] OR "resistant hypertension"[Title])

2. ("renal denervation"[Title/Abstract] OR "rdn"[Title/Abstract] OR "sympathetic renal denervation"[Title/Abstract] OR "central anastomosis"[Title/Abstract] OR "central arteriovenous anastomosis"[Title/Abstract] OR "mineralocorticoid receptor antagonist"[Title/Abstract] OR "canrenone"[Title/Abstract] OR "eplerenone"[Title/Abstract] OR "finerenone"[Title/Abstract] OR "spironolactone"[Title/Abstract] OR "aldosterone synthase inhibitor"[Title/Abstract] OR "aldosterone inhibitor"[Title/Abstract] OR "baxdrostat"[Title/Abstract] OR "lorundrostat"[Title/Abstract] OR "endothelin receptor antagonist"[Title/Abstract] OR "endothelin antagonist"[Title/Abstract] OR "endothelin blocker"[Title/Abstract] OR "aprocitentan"[Title/Abstract] OR "darusentan"[Title/Abstract] OR "sodium glucose cotransporter 2 inhibitor"[Title/Abstract] OR "gliflozin"[Title/Abstract] OR "bexagliflozin"[Title/Abstract] OR "canagliflozin"[Title/Abstract] OR "dapagliflozin"[Title/Abstract] OR "empagliflozin"[Title/Abstract] OR "ertugliflozin"[Title/Abstract] OR "alpha adrenergic receptor blocking agent"[Title/Abstract] OR "alpha blocker"[Title/Abstract] OR "alfuzosin"[Title/Abstract] OR "doxazosin"[Title/Abstract] OR "silodosin"[Title/Abstract] OR "tamsulosin"[Title/Abstract] OR "terazosin"[Title/Abstract] OR "alpha agonist"[Title/Abstract] OR "clonidine"[Title/Abstract] OR "guanfacine"[Title/Abstract] OR "methyldopa"[Title/Abstract] OR "vasodilatator"[Title/Abstract] OR "hydralazine"[Title/Abstract] OR "minoxidil"[Title/Abstract] OR "aerobic exercise"[Title/Abstract]) OR "exercise training"[Title/Abstract])

3. ("random*"[Title/Abstract] OR "factorial*"[Title/Abstract] OR "crossover"[Title/Abstract] OR "cross-over"[Title/Abstract] OR "cross-over"[Title/Abstract] OR "placebo*"[Title/Abstract] OR "double blind"[Title/Abstract] OR "single blind"[Title/Abstract] OR "assign*"[Title/Abstract] OR "allocat*"[Title/Abstract] OR "volunteer*"[Title/Abstract] OR "crossover procedure"[Title/Abstract] OR "double-blind procedure"[Title/Abstract] OR "randomized controlled trial"[Title/Abstract] OR "single-blind procedure"[Title/Abstract])

4. #1 AND #2 AND #3 AND “humans”[MeSH Terms]

### 2.1.2 Embase

1. (hypertension*:ti OR 'uncontrolled hypertension*':ti OR 'resistant hypertension*':ti) AND [embase]/lim

2. ('renal denervation*':ti,ab OR 'rdn*':ti,ab OR 'sympathetic renal denervation*':ti,ab OR 'central anastomosis*':ti,ab OR 'central arteriovenous anastomosis':ti,ab OR 'mineralocorticoid receptor antagonist*':ti,ab OR 'canrenone*':ti,ab OR 'eplerenone*':ti,ab OR 'finrenone*':ti,ab OR 'spironolactone*':ti,ab OR 'aldosterone synthase inhibitor*':ti,ab OR 'aldosterone inhibitor*':ti,ab OR 'baxdrostat*' OR 'lorundrostat*':ti,ab OR 'endothelin receptor antagonist*':ti,ab OR 'endothelin antagonist*':ti,ab OR 'endothelin blockers*':ti,ab OR 'aprocitentan*':ti,ab OR 'darusentan*':ti,ab OR 'sodium glucose cotransporter 2 inhibitor':ti,ab OR 'gliflozin*':ti,ab OR 'bexagliflozin':ti,ab OR 'canagliflozin':ti,ab OR 'dapagliflozin':ti,ab OR 'empagliflozin*':ti,ab OR 'ertugliflozin*':ti,ab OR 'alpha adrenergic receptor blocking agent*':ti,ab OR 'alpha blockers*':ti,ab OR 'alfuzosin*':ti,ab OR 'doxazosin*':ti,ab OR 'silodosin*':ti,ab OR 'tamsulosin*':ti,ab OR 'terazosin*':ti,ab OR 'alpha adrenergic receptor stimulating agent*':ti,ab OR 'alpha agonist*':ti,ab OR 'clonidine*':ti,ab OR 'guanfacine*':ti,ab OR 'methyldopa*':ti,ab OR 'vasodilatator*':ti,ab OR 'hydralazine*':ti,ab OR 'minoxidil*':ti,ab OR 'aerobic exercise*':ti,ab OR 'exercise training*':ti,ab) AND [embase]/lim

3. (random* OR factorial* OR crossover* OR 'cross over*' OR 'cross-over*' OR placebo* OR 'doubl* adj blind*' OR 'singl* adj blind*' OR assign* OR allocat* OR volunteer* OR 'crossover-procedure*' OR 'double-blind procedure*' OR 'randomized controlled trial*' OR 'single-blind procedure*') AND [embase]/lim

4. #1 AND #2 AND #3 AND [humans]/lim

# 3. **Eligible interventions**

We included RCTs comparing baseline treatment (including ACE-i or ARBs, BB, CCB, MRAs [less than 30% of patients treated] or diuretic) vs. one of the following treatments for the management of uncontrolled hypertension:

**Aldosterone synthase inhibitors**:

- Baxdrostat
- Lorundrostat

**Alpha adrenergic receptor blocking agents**:

- Alfuzosin
- Doxazosin
- Silodosin
- Tamsulosin
- Terazosin

**Alpha adrenergic receptor stimulating agents**:

- Clonidine
- Guanfacine
- Methyldopa

**Angiotensinogen synthesis inhibitors**:

- Zilbesiran

**Central arteriovenous anastomosis**

**Direct vasodilator agents**:

- Hydralazine
- Minoxidil

**Endothelin receptor antagonists**:

- Aprocitentan
- Darusentan

**Lifestyle interventions**:

- Aerobic exercise

**Mineralocorticoid receptor antagonists**:

- Canrenone
- Eplerenone
- Finrenone
- Spironolactone

**Renal denervation**:

- Alcohol renal denervation
- Radiofrequency renal denervation
- Ultrasound renal denervation

**Sodium-glucose cotransporter 2 inhibitors**:

- Canagliflozin
- Dapagliflozin
- Empagliflozin
- Ertugliflozin

# 4. **Details of the data analysis**

Analyses were originally planned in patients with uncontrolled hypertension, with a subgroup analysis in patients with resistant hypertension (defined as a systolic blood pressure ≥140 mm Hg despite treatment with three or more antihypertensive drugs, including a diuretic, at full doses) prespecified for the primary outcome. During peer review, a reviewer recommended focusing the analyses on the resistant hypertension population, as this is the group for whom intensified pharmacological and device-based interventions are most commonly considered in clinical practice. We agreed with this consideration and, in response to the reviewer's recommendation, performed the full set of analyses in patients with resistant hypertension and present these results in the main manuscript.

## 4.1 **Measure of treatment effect**

The effect size was mean difference as each measure of blood pressure was measured on the same scale.

If information on standard deviations was missing, we derived standard deviations from standard errors, confidence intervals, t-values, or p-values as described in Section 7.7.3 of the Cochrane Handbook for Systematic Reviews.

# 5. **Characteristics of studies included in the analyses**

In the following table are reported the main characteristics of the studies included in the quantitative analyses.

## 5.1 **Design of the included studies**

| **Study** | **Main inclusion criteria** | **Main exclusion criteria** | **Screening phase** |
| --- | --- | --- | --- |
| **Aldosterone synthase inhibitors vs. baseline treatment** | | | |
| **Baxdrostat vs. baseline treatment**: | | | |
| Bax24^1^ | Patients with resistant hypertension (office BP ≥130/80 mm Hg) despite treatment with ≥ 3 antihypertensive medications including a diuretic. | Office systolic BP ≥ 170 mm Hg.  Office diastolic BP ≥ 110 mm Hg. | No phase of therapies standardization before enrolment. |
| BaxHTN^2^ | Patients with uncontrolled hypertension (office systolic BP ≥ 140 mm Hg) despite treatment of ≥ 2 antihypertensive medications. | Office systolic BP ≥ 170 mm Hg.  Office diastolic BP ≥ 110 mm Hg. | No phase of therapies standardization before enrolment. |
| BrigHTN^3^ | Patients with resistant hypertension (office BP ≥130/80 mm Hg) despite treatment with ≥ 3 antihypertensive medications including a diuretic. | eGFR < 45 mL/min/1.73 m^2^.  Office systolic BP ≥ 180 mm Hg.  Office diastolic BP ≥ 110 mm Hg. | No phase of therapies standardization before enrolment. |
| **Lorundrostat vs.baseline treatment**: | | | |
| Advance-HTN^4^ | Patients with uncontrolled hypertension (office BP ≥ 140/90 mm Hg) despite treatment of ≥ 2 antihypertensive medications. | eGFR < 45 mL/min/1.73 m^2^.  Office systolic BP ≥ 180 mm Hg.  Office diastolic BP ≥ 110 mm Hg. | 3-weeks standardized regimen. Participants taking two antihypertensive medications at enrolment were assigned to receive olmesartan once daily at a dose of 40 mg, along with indapamide at a dose of 2.5 mg or hydrochlorothiazide at a dose of 25 mg. For participants taking three to five antihypertensive medications at enrolment, amlodipine at a dose of 10 mg daily was also included  in the standardized regimen. |
| Launch-HTN^5^ | Patients with uncontrolled hypertension (office BP ≥ 135/90 mm Hg) despite treatment of ≥ 2 antihypertensive medications including a diuretic. | eGFR < 45 mL/min/1.73 m^2^.  Office systolic BP ≥ 180 mm Hg.  Office diastolic BP ≥ 110 mm Hg. | No phase of therapies standardization before enrolment. |
| Target-HTN^6^ | Patients with uncontrolled hypertension (office systolic BP ≥ 130 mm Hg) despite treatment of ≥ 2 antihypertensive medications. | eGFR < 60 mL/min/1.73 m^2^.  Office systolic BP ≥ 175 mm Hg.  Office diastolic BP ≥ 100 mm Hg. | No phase of therapies standardization before enrolment. |
| **Angiotensinogen synthesis inhibitors vs. baseline treatment** | | | |
| **Zilbesiran vs. baseline treatment**: | | | |
| KARDIA-3^#7^ | Patients with uncontrolled hypertension (office systolic BP ≥ 140 mm Hg) despite treatment of ≥ 2 antihypertensive medications. | eGFR < 45 mL/min/1.73 m^2^. | No phase of therapies standardization before enrolment. |
| **Endothelin receptor antagonists vs. baseline treatment** | | | |
| **Aprocitentan vs. baseline treatment**: | | | |
| PRECISION^8^ | Patients with resistant hypertension (office systolic BP ≥140 mm Hg) despite standardized treatment with ≥ 3 antihypertensive medications including a diuretic. | eGFR < 15 mL/min/1.73 m^2^.  Office systolic BP ≥ 180 mm Hg.  Office diastolic BP ≥ 110 mm Hg. | 4-weeks standardized regimen.  During screening, all patients switched their individual antihypertensive therapies (except β blockers) to standardized background therapy, a single-pill triple combination including amlodipine, valsartan and hydrochlorothiazide, at fixed doses of either 5 mg, 160 mg, or 25 mg; or 10 mg, 160 mg, or 25 mg, respectively. |
| **Darusentan vs. baseline treatment**: | | | |
| Black et al.^9^ | Patients with resistant hypertension (office systolic BP ≥140 mm Hg) despite treatment with ≥ 3 antihypertensive medications including a diuretic. | eGFR < 30 mL/min/1.73 m^2^.  Office systolic BP ≥ 180 mm Hg.  Office diastolic BP ≥ 110 mm Hg. | No phase of therapies standardization before enrolment. |
| Weber et al.^10^ | Patients with resistant hypertension (office systolic BP ≥140 mm Hg) despite treatment with ≥ 3 antihypertensive medications including a diuretic. | eGFR < 30 mL/min/1.73 m^2^.  Office systolic BP ≥ 180 mm Hg.  Office diastolic BP ≥ 110 mm Hg. | No phase of therapies standardization before enrolment. |
| **Endothelin receptor antagonists vs. α-blockers vs. baseline treatment** | | | |
| **Darusentan vs. guanfacine vs. baseline treatment**: | | | |
| Bakris et al.^11^ | Patients with resistant hypertension (office systolic BP ≥140 mm Hg) despite treatment with ≥ 3 antihypertensive medications including a diuretic. | eGFR < 30 mL/min/1.73 m^2^.  Office systolic BP ≥ 180 mm Hg.  Office diastolic BP ≥ 110 mm Hg. | No phase of therapies standardization before enrolment. |
| **Mineralocorticoid receptor antagonists vs. α-agonists** | | | |
| **Spironolactone vs. clonidine**: | | | |
| ReHOT^12^ | Patients with resistant hypertension (BP above treatment goals) despite treatment with ≥ 3 antihypertensive medications including a diuretic. | eGFR < 30 mL/min/1.73 m^2^.  Office systolic BP ≥ 220 mm Hg. | 12-weeks standardized regimen.  3 antihypertensive drugs (chlorthalidone, enalapril or losartan, and amlodipine). |
| **Mineralocorticoid receptor antagonists vs. baseline treatment** | | | |
| **Spironolactone vs. baseline treatment**: | | | |
| Abolghasmi et al.^#13^ | Patients with resistant hypertension (office BP ≥140/90 mm Hg) despite treatment with ≥ 3 antihypertensive medications including a diuretic. | Not specified. | No phase of therapies standardization before enrolment. |
| ASPIRANT-EXT^14^ | Patients with resistant hypertension (office BP ≥140/90 mm Hg) despite treatment with ≥ 3 antihypertensive medications including a diuretic. | eGFR < 40 mL/min/1.73 m^2^.  Office systolic BP ≥ 180 mm Hg.  Office diastolic BP ≥ 110 mm Hg. | No phase of therapies standardization before enrolment. |
| Djoumessi et al.^#15^ | Patients with resistant hypertension (office BP ≥140/90 mm Hg) despite treatment with ≥ 3 antihypertensive medications including a diuretic. | eGFR < 30 mL/min/1.73 m^2^. | No phase of therapies standardization before enrolment. |
| Ni et al.^#16^ | Patients with resistant hypertension (office BP ≥140/90 mm Hg) despite treatment with ≥ 3 antihypertensive. | Not specified. | No phase of therapies standardization before enrolment. |
| Oxlund et al.^17^ | Patients with resistant hypertension (office BP ≥130/80 mm Hg) despite treatment with ≥ 3 antihypertensive medications including a diuretic. | eGFR < 50 mL/min/1.73 m^2^.  Office systolic BP ≥ 180 mm Hg.  Office diastolic BP ≥ 110 mm Hg. | No phase of therapies standardization before enrolment. |
| Yang et al.^18^ | Patients with resistant hypertension (office BP ≥140/90 mm Hg) despite treatment with ≥ 3 antihypertensive medications including a diuretic. | eGFR < 60 mL/min/1.73 m^2^. | No phase of therapies standardization before enrolment. |
| **Eplerenone vs. baseline treatment** | | | |
| Eguchi et al.^#19^ | Patients with resistant hypertension (office BP ≥135/85 mm Hg) despite treatment with ≥ 3 antihypertensive medications including a diuretic. | eGFR < 50 mL/min/1.73 m^2^. | No phase of therapies standardization before enrolment. |
| **Renal denervation vs. mineralocorticoid receptor antagonists** | | | |
| **Radiofrequency-RDN vs. spironolactone**: | | | |
| DENERVHTA^20^ | Patients with resistant hypertension (office systolic BP ≥150 mm Hg; ABPM systolic ≥ 140 mm Hg) despite treatment with ≥ 3 antihypertensive medications including a diuretic. | eGFR < 45 mL/min/1.73 m^2^. | No phase of therapies standardization before enrolment. |
| PRAGUE-15^21^ | Patients with resistant hypertension (office systolic BP ≥140 mm Hg; ABPM systolic ≥ 130 mm Hg) despite treatment with ≥ 3 antihypertensive medications including a diuretic. | Not specified. | No phase of therapies standardization before enrolment. |
| **Renal denervation vs. baseline treatment** | | | |
| **Alcohol-RDN vs. baseline treatment**: | | | |
| TARGET BP I^22^ | Patients with uncontrolled hypertension (office BP ≥ 150/90 mm Hg; ABPM systolic ≥150 mm Hg) despite treatment of ≥ 2 antihypertensive medications. | eGFR < 45 mL/min/1.73 m^2^.  Office systolic BP ≥ 180 mm Hg. | No phase of therapies standardization before enrolment. |
| **Radiofrequency-RDN vs. baseline treatment**: | | | |
| DENERHTN^23^ | Patients with resistant hypertension (office BP ≥140/90 mm Hg) despite standardized treatment with ≥ 3 antihypertensive medications including a diuretic. | eGFR < 40 mL/min/1.73 m^2^. | 4-weeks standardised regimen.  Triple therapy of sustained-release indapamide 1.5 mg, ramipril 10 mg (or irbesartan 300 mg in case of cough), and amlodipine 10 mg (or 5 mg in case of leg oedema). |
| Desch et al.^24^ | Patients with resistant hypertension (ABPM ≥135/90 mm Hg) despite treatment with ≥ 3 antihypertensive medications including a diuretic. | eGFR < 45 mL/min/1.73 m^2^.  ABPM systolic BP ≥ 150 mm Hg.  ABPM diastolic BP ≥ 95 mm Hg. | No phase of therapies standardization before enrolment. |
| Iberis-HTN^25^ | Patients with resistant hypertension (office BP ≥150/90 mm Hg; ABPM systolic ≥ 135 mm Hg) despite standardized treatment with ≥ 3 antihypertensive medications including a diuretic. | eGFR < 40 mL/min/1.73 m^2^.  Office systolic BP > 180 mm Hg.  ABPM systolic > 170 mm Hg. | 4-weeks standardized regimen.  5 mg of amlodipine and a 2-drug fixed-dose single pill with 80 mg of valsartan and 12.5 mg of hydrochlorothiazide combined. |
| Netrod-RDN^26^ | Patients with uncontrolled hypertension (office BP ≥140/90 mm Hg; ABPM systolic ≥ 135 mm Hg) despite standardized treatment with ≥ 2 antihypertensive medications. | eGFR < 45 mL/min/1.73 m^2^.  Office systolic BP ≥ 180 mm Hg.  Office diastolic BP ≥ 110 mm Hg. | 4-weeks standardized regimen.  Nifedipine gastrointestinal therapeutic system and hydrochlorothiazide. |
| RADIANCE-HTN TRIO^27^ | Patients with resistant hypertension (office BP ≥140/90 mm Hg) despite standardized treatment with ≥ 3 antihypertensive medications including a diuretic. | eGFR of <40 mL/min/1.73 m^2^. | 4-weeks standardized regimen.  Single-pill, fixed-dose, daily combination of amlodipine 10 mg (or 5 mg in the event of severe leg oedema), valsartan 160 mg (or olmesartan 40 mg depending upon medication availability in each country), and hydrochlorothiazide 25 mg. |
| ReSET^28^ | Patients with resistant hypertension (ABPM systolic ≥ 145 mm Hg) despite treatment with ≥ 3 antihypertensive medications including a diuretic. | eGFR of <30 mL/min/1.73 m^2^. | No phase of therapies standardization before enrolment. |
| SMART^29^ | Patients with uncontrolled hypertension (office SBP ≥150 mm Hg) despite standardized treatment with ≥ 2 medications. | eGFR of <45 mL/min/1.73 m^2^. | 4-weeks standardized regimen.  Patients go through a phase of standardized antihypertensive therapy using ACE-I, ARBs, CCBs, diuretic, β-blockers and α-blockers. |
| SPYRAL HTN-ON MED^30^ | Patients with uncontrolled hypertension (office BP ≥ 150/90 mm Hg; ABPM systolic BP ≥140 mm Hg) despite treatment with ≥ 1 medication*. | eGFR of <45 mL/min/1.73 m^2^.  Office systolic BP ≥ 180 mm Hg.  ABPM systolic BP ≥ 170 mm Hg. | No phase of therapies standardization before enrolment. |
| SYMPATHY^31^ | Patients with uncontrolled hypertension (ABPM systolic BP ≥ 135 mm Hg) despite treatment of ≥ 2 antihypertensive medications. | eGFR of <20 mL/min/1.73 m^2^. | No phase of therapies standardization before enrolment. |
| SYMPLICITY HTN-2^32^ | Patients with resistant hypertension (office systolic BP ≥ 160) despite treatment with ≥ 3 medications including a diuretic. | eGFR of <45 mL/min/1.73 m^2^. | No phase of therapies standardization before enrolment. |
| SYMPLICITY HTN-3^33^ | Patients with resistant hypertension (office systolic BP ≥ 160) despite treatment with ≥ 3 medications including a diuretic. | eGFR of <45 mL/min/1.73 m^2^. | No phase of therapies standardization before enrolment. |
| **Ultrasound-RDN vs. baseline treatment**: | | | |
| REQUIRE^34^ | Patients with resistant hypertension (office BP ≥ 150/90 mm Hg) despite treatment with ≥ 3 medications including a diuretic. | eGFR of <40 mL/min/1.73 m^2^. | No phase of therapies standardization before enrolment. |
| WAVE IV^35^ | Patients with resistant hypertension (office systolic BP ≥ 160 mm Hg and ABPM systolic BP ≥ 135 mm Hg) despite treatment with ≥ 3 medications including a diuretic. | Not specified. | No phase of therapies standardization before enrolment. |

ABPM: ambulatory blood pressure monitoring; BP: blood pressure.

*Only the group of patients with ≥ 3 medication was included in the analyses.

#Not included in the network meta-analysis.

## 5.2 **Baseline characteristics of the included studies**

| **Study** | **Treatment**  **comparison** | **Age*** | **Females**  **(%)** | **Asian**  **(%)** | **Black**  **(%)** | **White**  **(%)** | **OSAS**  **(%)** | **SBP*** | **DBP*** | **BMI*** | **eGFR*** |
| --- | --- | --- | --- | --- | --- | --- | --- | --- | --- | --- | --- |
| **Aldosterone synthase inhibitors vs. baseline treatment** | | | | | | | | | | | |
| **Baxdrostat vs. baseline treatment**: | | | | | | | | | | | |
| Bax24 | Treatment | NA | NA | NA | NA | NA | NA | NA | NA | NA | NA |
|  | Control | NA | NA | NA | NA | NA | NA | NA | NA | NA | NA |
| BaxHTN | Treatment | 60.8 | 37 | 26 | 6 | 63 | NA | 149.5 | 86.9 | 31.4 | 85.5 |
|  | Control | 61.9 | 39 | 27 | 8 | 63 | NA | 149.0 | 85.8 | 31.1 | 84.1 |
| BrigHTN | Treatment | 61.8 | 46 | 2 | 29 | 69 | NA | 147.7 | 87.8 | 32.8 | 83.2 |
|  | Control | 63.8 | 39 | 3 | 23 | 74 | NA | 148.9 | 88.2 | 32.1 | 85.5 |
| **Lorundrostat vs.baseline treatment**: | | | | | | | | | | | |
| Advance-HTN | Treatment | 61.1 | 42 | NA | 55 | 39 | NA | 152.9 | 85.0 | 31.8 | 76.5 |
|  | Control | 59.1 | 35 | NA | 46 | 43 | NA | 155.1 | 90.7 | 32.2 | 73.6 |
| Launch-HTN | Treatment | 61.6 | 46 | 2 | 28 | 39 | NA | 147.3 | 86.8 | 32.6 | 91.9 |
|  | Control | 61.8 | 46 | 2 | 32 | 65 | NA | 148.8 | 87.1 | 32.9 | 91.2 |
| Target-HTN | Treatment | 66.2 | 58 | 2 | 36 | 62 | NA | 142.0 | 81.0 | 31.1 | 78.9 |
|  | Control | 62.6 | 59 | 3 | 53 | 43 | NA | 142.9 | 83.8 | 31.9 | 81.6 |
| **Angiotensinogen synthesis inhibitors vs. baseline treatment** | | | | | | | | | | | |
| **Zilbesiran vs. baseline treatment**: | | | | | | | | | | | |
| KARDIA-3^#^ | Treatment | 66.4 | 48 | NA | 42 | NA | NA | 143.4 | 80.2 | NA | NA |
|  | Control | 66.3 | 38 | NA | 20 | NA | NA | 144.1 | 79.6 | NA | NA |
| **Endothelin receptor antagonists vs. baseline treatment** | | | | | | | | | | | |
| **Aprocitentan vs. baseline treatment**: | | | | | | | | | | | |
| PRECISION | Treatment | 61.5 | 41 | 5 | 12 | 83 | 15 | 153.2 | 87.8 | 34.0 | NA |
|  | Control | 62.2 | 41 | 5 | 11 | 83 | 13 | 153.3 | 87.1 | 33.3 | NA |
| **Darusentan vs. baseline treatment**: | | | | | | | | | | | |
| Black et al. | Treatment | 62.0 | 43 | NA | 30 | 70 | NA | 149.6 | 82.4 | 31.2 | 74.2 |
|  | Control | 63.0 | 36 | NA | 23 | 74 | NA | 149.0 | 79.7 | 32.6 | 79.3 |
| Weber et al. | Treatment | 61.6 | 48 | NA | NA | NA | NA | 150.9 | 86.3 | 31.7 | 78.3 |
|  | Control | 62.0 | 55 | NA | NA | NA | NA | 151.0 | 89.0 | 32.0 | 80.0 |
| **Endothelin receptor antagonists vs. α-blockers vs. baseline treatment** | | | | | | | | | | | |
| **Darusentan vs. guanfacine vs. baseline treatment**: | | | | | | | | | | | |
| Bakris et al. | Treatment_darusentan_ | 63.0 | 46 | NA | 10 | NA | NA | 151.0 | 88.0 | 32.0 | 82.0 |
|  | Treatment_guanfacine_ | 62.0 | 44 | NA | 11 | NA | NA | 152.0 | 87.0 | 32.0 | 80.0 |
|  | Control | 63.0 | 41 | NA | 9 | NA | NA | 152.0 | 89.0 | 31.0 | 77.0 |
| **Mineralocorticoid receptor antagonists vs. α-agonists** | | | | | | | | | | | |
| **Spironolactone vs. clonidine**: | | | | | | | | | | | |
| ReHOT | Treatment_clonidine_ | 54.0 | 56 | NA | NA | NA | NA | 180.1 | 105.2 | 31.5 | 86.0 |
|  | Treatment_clonidine_ | 56.3 | 53 | NA | NA | NA | NA | 181.1 | 107.1 | 30.0 | 94.5 |
| **Mineralocorticoid receptor antagonists vs. baseline treatment** | | | | | | | | | | | |
| **Spironolactone vs. baseline treatment**: | | | | | | | | | | | |
| Abolghasmi et al.^#^ | Treatment | 49.0 | 47 | NA | NA | NA | NA | 171.0 | 95.0 | NA | NA |
|  | Control | 50.0 | 46 | NA | NA | NA | NA | 170.0 | 93.0 | NA | NA |
| ASPIRANT-EXT | Treatment | 60.4 | 32 | NA | NA | NA | NA | 154.9 | 93.1 | 32.2 | NA |
|  | Control | 59.7 | 37 | NA | NA | NA | NA | 153.3 | 91.1 | 32.3 | NA |
| Djoumessi et al.^#^ | Treatment | 64.6 | 67 | NA | NA | NA | NA | 158.0 | 86.0 | 30.3 | 55.3 |
|  | Control | 61.0 | 38 | NA | NA | NA | NA | 158.0 | 94.0 | 30.5 | 74.0 |
| Ni et al.^#^ | Treatment | 55.7 | 40 | NA | NA | NA | NA | 153.5 | 92.5 | NA | NA |
|  | Control | 54.9 | 42 | NA | NA | NA | NA | 151.5 | 89.5 | NA | NA |
| Oxlund et al. | Treatment | 62.9 | 25 | NA | NA | NA | NA | 146.0 | 79.0 | 32.0 | NA |
|  | Control | 63.9 | 22 | NA | NA | NA | NA | 146.0 | 79.0 | 31.5 | NA |
| Yang et al. | Treatment | 44.7 | NA | NA | NA | NA | NA | 157.6 | 96.2 | 29.5 | NA |
|  | Control | 43.4 | NA | NA | NA | NA | NA | 150.7 | 93.8 | 28.7 | NA |
| **Eplerenone vs. baseline treatment** | | | | | | | | | | | |
| Eguchi et al.^#^ | Treatment | 60.0 | 29 | NA | NA | NA | NA | 145.0 | 80.0 | 27.5 | NA |
|  | Control | 65.9 | 50 | NA | NA | NA | NA | 146.0 | 77.0 | 28.1 | NA |
| **Renal denervation vs. mineralocorticoid receptor antagonists** | | | | | | | | | | | |
| **Radiofrequency-RDN vs. spironolactone**: | | | | | | | | | | | |
| DENERVHTA | Treatment_RDN_ | 61.9 | 45 | 0 | 0 | 100 | NA | 168.0 | 89.6 | 33.7 | 74.6 |
|  | Treatment_spironolactone_ | 64.9 | 31 | NA | NA | 85 | NA | 171.2 | 90.2 | 30.6 | 85.0 |
| PRAGUE-15 | Treatment_RDN_ | 56.0 | 23 | NA | NA | NA | NA | 159.0 | 92.0 | 31.2 | 90.0 |
|  | Treatment_spironolactone_ | 59.0 | 37 | NA | NA | NA | NA | 155.0 | 89.0 | 33.4 | 96.0 |
| **Renal denervation vs. baseline treatment** | | | | | | | | | | | |
| **Alcohol-RDN vs. baseline treatment**: | | | | | | | | | | | |
| TARGET BP I | Treatment | 56.7 | 24 | 0 | 30 | 16 | NA | 164.0 | 98.0 | 32.6 | 81.8 |
|  | Control | 55.6 | 28 | 1 | 38 | 20 | NA | 164.0 | 100.0 | 32.1 | 82.7 |
| **Radiofrequency-RDN vs. baseline treatment**: | | | | | | | | | | | |
| DENERHTN | Treatment | 55.2 | 46 | NA | NA | 79 | 30 | 159.3 | 93.3 | 30.7 | 88.0 |
|  | Control | 55.2 | 40 | NA | NA | 77 | 25 | 155.9 | 91.4 | 29.7 | 90.0 |
| Desch et al. | Treatment | 64.5 | 23 | NA | NA | NA | NA | 140.2 | 78.2 | 31.9 | 79.0 |
|  | Control | 57.4 | 31 | NA | NA | NA | NA | 140.4 | 80.6 | 31.2 | 84.0 |
| Iberis-HTN | Treatment | 46.4 | 22 | NA | NA | NA | NA | 159.1 | 98.8 | 27.7 | 100.0 |
|  | Control | 46.3 | 20 | NA | NA | NA | NA | 159.4 | 99.4 | 27.8 | 103.7 |
| Netrod-RDN | Treatment | NA | NA | NA | NA | NA | NA | NA | NA | NA | NA |
|  | Control | NA | NA | NA | NA | NA | NA | NA | NA | NA | NA |
| RADIANCE-HTN TRIO | Treatment | 52.0 | 19 | NA | 20 | 64 | 28 | 161.9 | 105.1 | 32.8 | 86.0 |
|  | Control | 52.8 | 21 | NA | 19 | 75 | 16 | 163.6 | 103.3 | 32.6 | 82.2 |
| ReSET | Treatment | 54.3 | 25 | NA | NA | 97 | NA | 160.0 | 95.0 | 28.2 | NA |
|  | Control | 57.1 | 27 | NA | NA | 97 | NA | 166.0 | 90.0 | 28.8 | NA |
| SMART | Treatment | 44.5 | 15 | 100 | NA | NA | 7 | 158.5 | 100.0 | 29.1 | 98.2 |
|  | Control | 46.8 | 12 | 100 | NA | NA | 4 | 160.4 | 101.6 | 28.6 | 98.8 |
| SPYRAL HTN-ON MED | Treatment | 55.2 | 39 | 1 | 17 | 35 | 11 | 163.0 | 101.2 | 31.4 | 81.9 |
|  | Control | 54.6 | 38 | 3 | 19 | 37 | 18 | 163.1 | 101.5 | 31.2 | 81.9 |
| SYMPATHY | Treatment | 62.0 | 58 | NA | NA | 97 | NA | 170.3 | 96.1 | 28.6 | 77.0 |
|  | Control | 60.0 | 70 | NA | NA | 96 | NA | 164.7 | 94.4 | 29.4 | 80.0 |
| SYMPLICITY HTN-2 | Treatment | 58.0 | 35 | NA | NA | 98 | NA | 178.0 | 97.0 | 31.0 | 77.0 |
|  | Control | 58.0 | 50 | NA | NA | 96 | NA | 178.0 | 98.0 | 31.0 | 86.0 |
| SYMPLICITY HTN-3 | Treatment | 57.9 | 41 | 1 | 25 | 73 | 26 | 179.7 | 96.5 | 34.2 | 72.8 |
|  | Control | 56.2 | 36 | 0 | 29 | 70 | 32 | 180.2 | 98.9 | 33.9 | 74.0 |
| **Ultrasound-RDN vs. baseline treatment**: | | | | | | | | | | | |
| REQUIRE | Treatment | 50.7 | 30 | NA | NA | NA | 16 | 157.6 | 97.7 | 29.5 | 74.2 |
|  | Control | 55.6 | 21 | NA | NA | NA | 12 | 160.4 | 95.3 | 28.4 | 69.6 |
| WAVE IV | Treatment | 60.3 | 19 | NA | NA | NA | NA | 181.1 | 99.5 | 29.9 | 81.8 |
|  | Control | 62.0 | 36 | NA | NA | NA | NA | 184.8 | 100.3 | 29.8 | 76.3 |

See table in section 4.1 for references

BMI: body mass index DBP: diastolic blood pressure; eGFR: estimated glomerular filtration rate; NA: not available; OSAS: Obstructive Sleep Apnea Syndrome; SBP: systolic blood pressure.

* Mean or median as reported in the original study.

#Not included in the network meta-analysis

## 5.3 **Background antihypertensive drugs used in the included studies**

We collected data for studies which have not used a standardized regiment of antihypertensive drugs prior to enrolment.

For ACE-I or ARBs, some studies reported the proportion of patients taking either ACE-Is or ARBs, while others reported the proportions separately for ACE-Is and for ARBs. Since more studies reported data in the former way, we chose to collect the data accordingly.

In some trials, the type of diuretic used was not specified. Therefore, these may have included MRAs. Nevertheless, we included these studies in the analyses to ensure a larger sample size.

| **Study** | **Treatment**  **comparison** | **ACE-i or ARBs**  **(%)** | **β-blockers**  **(%)** | **CCBs**  **(%)** | **Diuretic**  **(%)** | **MRAs**  **(%)** | **Drugs**  **(mean or median or %)** |
| --- | --- | --- | --- | --- | --- | --- | --- |
| **Aldosterone synthase inhibitors vs. baseline treatment** | | | | | | | |
| **Baxdrostat vs. baseline treatment**: | | | | | | | |
| Bax24 | Treatment | 93 | 45 | 89 | 100 | Not  applicable^‡^ | 3.7 drugs  3 drugs: 52%  4 drugs: 34%  5+ drugs: 14% |
|  | Control | 95 | 51 | 87 | 100 |  | 3.6 drugs  3 drugs: 42%  4 drugs: 38%  5+ drugs: 20% |
| BaxHTN | Treatment | 91 | 33 | 71 | 99 | Not  applicable^‡^ | 3.0 drugs  2 drugs: 27%  3 drugs: 40%  4 drugs: 24%  5+ drugs: 9% |
|  | Control | 89 | 34 | 67 | 100 |  | 3.0 drugs  2 drugs: 26%  3 drugs: 42%  4 drugs: 27%  5+ drugs: 5% |
| BrigHTN | Treatment | 92 | 57 | 67 | Not  applicable† | Not  applicable^‡^ | 3 drugs: 40%  4 drugs: 24%  5+ drugs: 9% |
|  | Control | 91 | 68 | 68 |  |  | 3 drugs: 62%  4 drugs: 22%  5+ drugs: 12% |
| **Lorundrostat vs.baseline treatment**: | | | | | | | |
| Advance-HTN | Standardized antihypertensive regimen (see table in section 5.1) | | | | | | |
| Launch-HTN | Treatment | 87 | NA | 52 | Not  applicable† | Not  applicable^‡^ | NA |
|  | Control | 83 | NA | 50 |  |  | NA |
| Target-HTN | Treatment | 79 | NA | NA | 57 | Not  applicable^‡^ | NA |
|  | Control | 73 | NA | NA | 53 |  | NA |
| **Angiotensinogen synthesis inhibitors vs. baseline treatment** | | | | | | | |
| **Zilbesiran vs. baseline treatment**: | | | | | | | |
| KARDIA-3^#^ | Treatment | 91 | NA | 54 | 68 | NA | NA |
|  | Control | 89 | NA | 66 | 63 | NA | NA |
| **Endothelin receptor antagonists vs. baseline treatment** | | | | | | | |
| **Aprocitentan vs. baseline treatment**: | | | | | | | |
| PRECISION | Standardized antihypertensive regimen (see table in section 5.1) | | | | | | |
| **Darusentan vs. baseline treatment**: | | | | | | | |
| Black et al. | Treatment | 93 | 49 | 65 | Not  applicable† | NA | NA |
|  | Control | 92 | 44 | 72 |  | NA | NA |
| Weber et al. | Treatment | 97 | 67 | 75 | Not  applicable† | NA | 4+ drugs: 59% |
|  | Control | 96 | 64 | 73 |  | NA | 4+ drugs: 57% |
| **Endothelin receptor antagonists vs. α-blockers vs. baseline treatment** | | | | | | | |
| **Darusentan vs. guanfacine vs. baseline treatment**: | | | | | | | |
| Bakris et al. | Treatment_darusentan_ | 96 | 62 | 66 | Not  applicable† | NA | 4+ drugs: 36% |
|  | Treatment_guanfacine_ | 96 | 61 | 69 |  | NA | 4+ drugs: 38% |
|  | Control | 96 | 58 | 63 |  | NA | 4+ drugs: 36% |
| **Mineralocorticoid receptor antagonists vs. α-agonists** | | | | | | | |
| **Spironolactone vs. clonidine**: | | | | | | | |
| ReHOT | Standardized antihypertensive regimen (see table in section 5.1) | | | | | | |
| **Mineralocorticoid receptor antagonists vs. baseline treatment** | | | | | | | |
| **Spironolactone vs. baseline treatment**: | | | | | | | |
| Abolghasmi et al.^#^ | Treatment | NA | NA | NA | Not  applicable^†^ | Not  applicable^‡^ | NA |
|  | Control | NA | NA | NA |  |  | NA |
| ASPIRANT-EXT | Treatment | NA | 72 | 88 | Not  applicable^†^ | Not  applicable^‡^ | 4.0 drugs |
|  | Control | NA | 86 | 75 |  |  | 4.0 drugs |
| Djoumessi et al.^#^ | Treatment | NA | NA | NA | Not  applicable^†^ | Not  applicable^‡^ | NA |
|  | Control | NA | NA | NA |  |  | NA |
| Ni et al.^#^ | Treatment | 55 | 100 | 65 | NA | Not  applicable^‡^ | NA |
|  | Control | 53 | 100 | 67 | NA |  | NA |
| Oxlund et al. | Treatment | NA | 50 | 84 | Not  applicable^†^ | Not  applicable^‡^ | NA |
|  | Control | NA | 41 | 84 |  |  | NA |
| Yang et al. | Treatment | NA | NA | NA | Not  applicable^†^ | Not  applicable^‡^ | NA |
|  | Control | NA | NA | NA |  |  | NA |
| **Eplerenone vs. baseline treatment** | | | | | | | |
| Eguchi et al.^#^ | Treatment | NA | NA | NA | Not  applicable^†^ | Not  applicable^‡^ | NA |
|  | Control | NA | NA | NA |  |  | NA |
| **Renal denervation vs. mineralocorticoid receptor antagonists** | | | | | | | |
| **Radiofrequency-RDN vs. spironolactone**: | | | | | | | |
| DENERVHTA | Treatment_RDN_ | 100 | 55 | 91 | Not  applicable^†^ | Not  applicable^‡^ | 4.3 drugs |
|  | Treatment_spironolactone_ | 92 | 77 | 69 |  |  | 3.9 drugs |
| PRAGUE-15 | Treatment_RDN_ | 100 | 66 | 89 | Not  applicable^†^ | Not  applicable^‡^ | NA |
|  | Treatment_spironolactone_ | 100 | 69 | 89 |  |  | NA |
| **Renal denervation vs. baseline treatment** | | | | | | | |
| **Alcohol-RDN vs. baseline treatment**: | | | | | | | |
| TARGET BP I | Treatment | NA | 47 | 61 | 54 | 16 | 2 drugs: 22%  3 drugs: 32%  4 drugs: 28%  5+ drugs: 18% |
|  | Control | NA | 48 | 61 | 52 | 23 | 2 drugs: 24%  3 drugs: 26%  4 drugs: 28%  5+ drugs: 22% |
| **Radiofrequency-RDN vs. baseline treatment**: | | | | | | | |
| DENERHTN | Standardized antihypertensive regimen (see table in section 5.1) | | | | | | |
| Desch et al. | Treatment | NA | 91 | 69 | Not  applicable^†^ | 3 | 4.4 drugs  5+ drugs: 40% |
|  | Control | NA | 94 | 64 |  | 6 | 4.3 drugs  5+ drugs: 39% |
| Iberis-HTN | Standardized antihypertensive regimen (see table in section 5.1) | | | | | | |
| Netrod-RDN | Standardized antihypertensive regimen (see table in section 5.1) | | | | | | |
| RADIANCE-HTN TRIO | Standardized antihypertensive regimen (see table in section 5.1) | | | | | | |
| ReSET | Treatment | NA | 81 | 53 | Not  applicable^†^ | 22 | 4.1 drugs |
|  | Control | NA | 76 | 85 |  | 21 | 4.2 drugs |
| SMART | Standardized antihypertensive regimen (see table in section 5.1) | | | | | | |
| SPYRAL HTN-ON MED | Treatment | 77 | 18 | 53 | 41^§^ | NA | NA |
|  | Control | 76 | 18 | 56 | 44 | NA | NA |
| SYMPATHY | Treatment | NA | 63 | 63 | 73 | 24 | 3.7 drugs |
|  | Control | NA | 59 | 61 | 59 | 23 | 3.4 drugs |
| SYMPLICITY HTN-2 | Treatment | 96 | 83 | 79 | 71 | 17 | 5.2 drugs  5+ drugs: 67% |
|  | Control | 94 | 69 | 93 | 74 | 17 | 5.3 drugs  5+ drugs: 57% |
| SYMPLICITY HTN-3 | Treatment | NA | 85 | 70 | 98 | 23 | 5.1 drugs |
|  | Control | NA | 86 | 73 | 100 | 29 | 5.2 drugs |
| **Ultrasound-RDN vs. baseline treatment**: | | | | | | | |
| REQUIRE | Treatment | 99 | 35 | 91 | 93 | 25 | 3 drugs: 46%  4 drugs: 29%  5+ drugs: 25% |
|  | Control | 99 | 37 | 88 | 94 | 15 | 3 drugs: 44%  4 drugs: 34%  5+ drugs: 22% |
| WAVE IV | Treatment | NA | 64 | 80 | 93 | 33 | 4.3 drugs  5+ drugs: 43% |
|  | Control | NA | 72 | 74 | 96 | 26 | 5.0 drugs  5+ drugs: 51% |

See table in section 4.1 for references.

ACE-i: angiotensin converting enzyme inhibitors; ARBs: angiotensin receptor blockers; CCBs: calcium channel blockers; MRAs: mineralocorticoid receptor antagonists; NA: not available.

†Treatment with diuretic required for inclusion.

‡Patients were randomized to MRAs, MRAs use was an exclusion criterion, or discontinuation of MRAs was required for study inclusion.

§Not specified what type of diuretics; therefore, it may include patients treated with MRAs.

#Not included in the network meta-analysis.

## 5.4 **Reason for exclusion from the quantitative synthesis**

| **Study** | **Reason for exclusion** |
| --- | --- |
| KARDIA-3 | No results reported. |
| Abolghasmi et al. | Given the equivocal findings and substantial risk of bias reported in previous studies,^36^ the study was excluded from the quantitative synthesis after discussion, considering its limited sample size. |
| Djoumessi et al. | The standard deviation for the reduction in SBP from baseline was not directly reported; therefore, given the limited sample size of the study, we decided to exclude it from the quantitative synthesis. |
| Ni et al. | The standard deviation for the reduction in SBP from baseline was not directly reported; therefore, given the limited sample size of the study, we decided to exclude it from the quantitative synthesis. |
| Eguchi et al. | The standard deviation for the reduction in SBP from baseline was not directly reported; therefore, given the limited sample size of the study, we decided to exclude it from the quantitative synthesis. |

See table in section 4.1 for references.

# 6. **Transitivity assessment**

We evaluated the assumption of transitivity by plotting the distribution of the following potential effect modifiers across the available comparisons as recommended by the Cochrane handbook:

- Mean age
- Proportion of females
- Baseline systolic blood pressure
- Proportion of patients taking mineralocorticoid receptor antagonists
- Year of study publication
- Study duration

## 6.1 **Mean age**

- Individual pharmacological and device-based interventions

- Pharmacological and device-based intervention classes

## 6.2 **Proportion of females**

- Individual pharmacological and device-based interventions

- Pharmacological and device-based intervention classes

## 6.3 **Baseline systolic blood pressure**

- Individual pharmacological and device-based interventions

- Pharmacological and device-based intervention classes

## 6.4 **Year of study publication**

- Individual pharmacological and device-based interventions

- Pharmacological and device-based intervention classes

## 6.5 **Study duration**

- Individual pharmacological and device-based interventions

- Pharmacological and device-based intervention classes

# 7. **Resistant hypertension**

In these analyses were included studies of patients with resistant hypertension as defined by the presence of a SBP of 140 mm Hg or greater despite treatment with three or more antihypertensive drugs, including a diuretic, at full doses.

For each outcome of interest, the following analyses and graphical displays were presented for both individual pharmacological and device-based interventionss and their corresponding classes, within the uncontrolled hypertension subgroup:

- Network plot

- Forest-plot of network meta-analysis (baseline treatment as reference)

- Forest-plot of pairwise meta-analyses

- League table

## 7.2 **Office systolic blood pressure**

### 7.2.1 Network plot

- Individual pharmacological and device-based interventions

The lines link treatments that were directly compared in trials. The thickness of the lines corresponds to the number of trials evaluating the comparison. The size of the circles (nodes) corresponds to the number of participants assigned to the treatment. RDN=renal denervation.

- Pharmacological and device-based intervention classes

The lines link treatments that were directly compared in trials. The thickness of the lines corresponds to the number of trials evaluating the comparison. The size of the circles (nodes) corresponds to the number of participants assigned to the treatment.

### 7.2.3 Forest-plot of pairwise meta-analysis

- Individual pharmacological and device-based interventions

Treatment comparisons are presented in alphabetical order. Summary effect size is calculated using pairwise meta-analysis of all studies in a specific comparison. CI=confidence interval. DBP=diastolic blood pressure. MD=mean difference. RDN=renal denervation.

- Pharmacological and device-based intervention classes

Treatment comparisons are presented in alphabetical order. Summary effect size is calculated using pairwise meta-analysis of all studies in a specific comparison. CI=confidence interval. DBP=diastolic blood pressure. MD=mean difference.

### 7.2.5 League table

- Pharmacological and device-based intervention classes

| **Aldosterone synthase**  **inhibitors** | -6.46 (-16.06 to 3.14) | -0.72 (-11.81 to 10.26) | 4.17 (-6.58 to 14.99) | 0.01 (-10.21 to 10.27) | -2.14 (-14.16 to 10.05) |
| --- | --- | --- | --- | --- | --- |
| **-6.50** **(-10.92 to -2.08)** | **Baseline treatment** | **5.74** **(0.34 to 11.15)** | **10.63** **(5.82 to 15.43)** | **6.48** **(2.95 to 10.00)** | 4.32 (-2.88 to 11.51) |
| NA | **5.05** **(2.45 to 7.66)** | **Endothelin receptor antagonists** | 4.88 (-1.92 to 11.86) | 0.73 (-5.60 to 7.20) | -1.43 (-8.69 to 5.92) |
| NA | **12.56** **(9.17 to 15.95)** | NA | **Mineralocorticoid**  **receptor antagonists** | -4.15 (-9.49 to 1.23) | -6.31 (-13.67 to 1.13) |
| NA | **6.20** **(1.79 to 10.62)** | NA | 0.64 (-6.27 to 4.99) | **Renal denervation** | -2.16 (-9.94 to 5.69) |
| NA | NA | NA | -1.40 (-7.94 to 5.14) | NA | **α-agonists** |

Treatments are in alphabetical order. In the upper right triangle are presented results from the network meta-analysis. In the lower left triangle are presented results from pairwise meta-analyses (direct comparisons). Each cell provides mean difference and the corresponding 95% CrI (or CI for pairwise meta-analyses). Bold print indicates 95% CrIs excluding no effect. NA=not available.

## 7.3 **Office diastolic blood pressure**

### 7.3.1 Network plot

- Individual pharmacological and device-based interventions

The lines link treatments that were directly compared in trials. The thickness of the lines corresponds to the number of trials evaluating the comparison. The size of the circles (nodes) corresponds to the number of participants assigned to the treatment. RDN=renal denervation.

- Pharmacological and device-based intervention classes

The lines link treatments that were directly compared in trials. The thickness of the lines corresponds to the number of trials evaluating the comparison. The size of the circles (nodes) corresponds to the number of participants assigned to the treatment.

### 7.3.2 Forest-plot of network meta-analysis

- Individual pharmacological and device-based interventions

Effect sizes are from the network meta-analysis. Treatments are ordered accordingly to the mean difference. The reference is the baseline treatment employed in each included trial. CrI=credible intervals. DBP=diastolic blood pressure. MD=mean difference. RDN=renal denervation.

- Pharmacological and device-based intervention classes

Effect sizes are from the network meta-analysis. Treatments are ordered accordingly to the mean difference. The reference is the baseline treatment employed in each included trial. CrI=credible intervals. DBP=diastolic blood pressure. MD=mean difference.

### 7.3.4 Forest-plot of pairwise meta-analysis

- Individual pharmacological and device-based interventions

Treatment comparisons are presented in alphabetical order. Summary effect size is calculated using pairwise meta-analysis of all studies in a specific comparison. CI=confidence interval. DBP=diastolic blood pressure. MD=mean difference. RDN=renal denervation.

- Pharmacological and device-based intervention classes

Treatment comparisons are presented in alphabetical order. Summary effect size is calculated using pairwise meta-analysis of all studies in a specific comparison. CI=confidence interval. DBP=diastolic blood pressure. MD=mean difference.

### 7.3.6 League table

- Individual pharmacological and device-based interventions

| **Baseline treatment** | 4.92 (-3.82 to 13.65) | 4.70 (-2.30 to 11.69) | **4.76** **(1.68 to 7.84)** | **6.25** **(2.25 to 10.25)** | 0.80 (-4.12 to 5.71) |
| --- | --- | --- | --- | --- | --- |
| NA | **Clonidine** | -0.22 (-11.87 to 11.00) | -0.16 (-9.39 to 8.84) | 1.33 (-6.67 to 9.19) | -4.12 (-14.54 to 5.96) |
| **4.68** **(2.85 to 6.51)** | NA | **Darusentan** | 0.06 (-7.64 to 7.99) | 1.55 (-6.38 to 10.15) | -3.90 (-12.59 to 4.75) |
| **4.43** **(1.82 to 7.04)** | NA | NA | **Radiofrequency**  **RDN** | 1.49 (-2.77 to 6.04) | -3.96 (-10.02 to 1.84) |
| **6.32** **(2.76 to 9.89)** | 1.30 (-2.48 to 5.08) | NA | -0.41 (-3.74 to 2.92) | **Spironolactone** | -5.45 (-12.13 to 0.65) |
| 1.01 (-2.53 to 4.55) | NA | NA | NA | NA | **Ultrasound**  **RDN** |

Treatments are in alphabetical order. In the upper right triangle are presented results from the network meta-analysis. In the lower left triangle are presented results from pairwise meta-analyses (direct comparisons). Each cell provides mean difference and the corresponding 95% CrI (or CI for pairwise meta-analyses). Bold print indicates 95% CrIs excluding no effect. NA=not available. RDN=renal denervation.

- Pharmacological and device-based intervention classes

| **Baseline treatment** | 4.70 (-2.42 to 11.83) | **5.85** **(1.79 to 9.91)** | **3.64** **(1.00 to 6.29)** | 4.56 (-4.26 to 13.39) |
| --- | --- | --- | --- | --- |
| **4.68** **(2.85 to 6.51)** | **Endothelin receptor antagonists** | 1.14 (-7.13 to 9.74) | -1.06 (-8.95 to 6.79) | -0.14 (-11.45 to 11.67) |
| **6.32** **(2.76 to 9.89)** | NA | **Mineralocorticoid**  **receptor antagonists** | -2.21 (-6.64 to 1.97) | -1.28 (-9.30 to 6.59) |
| **3.40** **(1.19 to 5.62)** | NA | 0.41 (-2.92 to 3.74) | **Renal denervation** | 0.92 (-8.00 to 10.10) |
| NA | NA | -1.30 (-5.08 to 2.48) | NA | **α-agonists** |

Treatments are in alphabetical order. In the upper right triangle are presented results from the network meta-analysis. In the lower left triangle are presented results from pairwise meta-analyses (direct comparisons). Each cell provides mean difference and the corresponding 95% CrI (or CI for pairwise meta-analyses). Bold print indicates 95% CrIs excluding no effect. NA=not available.

## 7.5 **24-hour systolic blood pressure**

### 7.5.1 Network plot

- Individual pharmacological and device-based interventions

The lines link treatments that were directly compared in trials. The thickness of the lines corresponds to the number of trials evaluating the comparison. The size of the circles (nodes) corresponds to the number of participants assigned to the treatment. NA=not available. RDN=renal denervation.

- Pharmacological and device-based intervention classes

The lines link treatments that were directly compared in trials. The thickness of the lines corresponds to the number of trials evaluating the comparison. The size of the circles (nodes) corresponds to the number of participants assigned to the treatment.

### 7.5.2 Forest-plot of network meta-analysis

- Individual pharmacological and device-based interventions

Effect sizes are from the network meta-analysis. Treatments are ordered accordingly to the mean difference. The reference is the baseline treatment employed in each included trial. CrI=credible intervals. MD=mean difference. RDN=renal denervation. SBP=systolic blood pressure.

- Pharmacological and device-based intervention classes

Effect sizes are from the network meta-analysis. Treatments are ordered accordingly to the mean difference. The reference is the baseline treatment employed in each included trial. CrI=credible intervals. MD=mean difference. SBP=systolic blood pressure.

### 7.5.4 Forest-plot of pairwise meta-analysis

- Individual pharmacological and device-based interventions

Treatment comparisons are presented in alphabetical order. Summary effect size is calculated using pairwise meta-analysis of all studies in a specific comparison. CI=confidence interval. DBP=diastolic blood pressure. MD=mean difference. RDN=renal denervation.

- Pharmacological and device-based intervention classes

Treatment comparisons are presented in alphabetical order. Summary effect size is calculated using pairwise meta-analysis of all studies in a specific comparison. CI=confidence interval. DBP=diastolic blood pressure. MD=mean difference.

### 7.5.5 League table

- Individual pharmacological and device-based interventions

| **Baseline treatment** | **14.01**  **(7.43 to 20.58)** | 5.18  (-3.19 to 13.55) | **6.99**  **(0.67 to 13.32)** | 2.01  (-4.28 to 8.30) | **4.41**  **(1.74 to 7.08)** | **9.66**  **(5.86 to 13.45)** | 1.64  (-3.16 to 6.43) |
| --- | --- | --- | --- | --- | --- | --- | --- |
| **14.00** **(10.89 to 17.11)** | **Baxdrostat** | -8.82  (-19.50 to 2.16) | -7.01  (-16.44 to 2.28) | **-11.99**  **(-21.59 to -2.59)** | **-9.59**  **(-16.84 to -2.37)** | -4.35  (-12.02 to 3.57) | -**12.37**  **(-20.70 to -4.01)** |
| NA | NA | **Clonidine** | 1.81  (-8.99 to 12.47) | -3.18  (-13.93 to 7.25) | -0.77  (-9.56 to 7.85) | 4.47  (-3.10 to 11.96) | -3.55  (-13.48 to 6.10) |
| **7.00** **(4.41 to 9.59)** | NA | NA | **Darusentan** | -4.99  (-11.34 to 1.50) | -2.59  (-9.53 to 4.44) | 2.66  (-4.76 to 10.45) | -5.36  (-13.42 to 2.77) |
| NA | NA | NA | NA | **Guanfacine** | 2.40  (-4.57 to 9.50) | **7.65**  **(0.32 to 15.43)** | -0.37  (-8.37 to 7.81) |
| **4.27** **(1.67 to 6.87)** | NA | NA | NA | NA | **Radiofrequency**  **RDN** | **5.25**  **(1.16 to 9.59)** | -2.77  (-8.32 to 2.81) |
| **9.98** **(7.29 to 12.68)** | NA | 4.50  (-0.06 to 9.06) | NA | NA | 6.27 (-3.99 to 16.54) | **Spironolactone** | **-8.018**  **(-14.28 to -1.93)** |
| 1.67 (-1.67 to 5.01) | NA | NA | NA | NA | NA | NA | **Ultrasound**  **RDN** |

Treatments are in alphabetical order. In the upper right triangle are presented results from the network meta-analysis. In the lower left triangle are presented results from pairwise meta-analyses (direct comparisons). Each cell provides mean difference and the corresponding 95% CrI (or CI for pairwise meta-analyses). Bold print indicates 95% CrIs excluding no effect. NA=not available. RDN=renal denervation.

- Pharmacological and device-based intervention classes

| **Aldosterone synthase inhibitors** | **-13.98**  **(-20.35 to -7.60)** | -6.45  (-15.27 to 2.36) | -4.89  (-12.16 to 2.45) | -10.22  (-17.19 to -3.40) | -10.95  (-19.09 to -2.80) |
| --- | --- | --- | --- | --- | --- |
| **14.00** **(10.89 to 17.11)** | **Baseline treatment** | **7.53**  **(1.71 to 13.34)** | **9.08**  **(5.69 to 12.46)** | **3.76**  **(1.48 to 6.03)** | 3.02  (-1.84 to 7.88) |
| NA | **7.00** **(4.41 to 9.59)** | **Endothelin receptor antagonists** | 1.55  (-4.94 to 8.07) | -3.76  (-10.19 to 2.56) | -4.50  (-10.22 to 1.47) |
| NA | **9.98** **(7.29 to 12.68)** | NA | **Mineralocorticoid receptor antagonists** | -5.32  (-9.22 to -1.65) | **-6.05**  **(-11.22 to -0.93)** |
| NA | **3.63** **(1.84 to 5.40)** | NA | -6.27 (-16.54 to 3.99) | **Renal denervation** | -0.74  (-5.97 to 4.76) |
| NA | NA | NA | -4.50 (-9.06 to 0.06) | NA | **α-agonists** |

Treatments are in alphabetical order. In the upper right triangle are presented results from the network meta-analysis. In the lower left triangle are presented results from pairwise meta-analyses (direct comparisons). Each cell provides mean difference and the corresponding 95% CrI (or CI for pairwise meta-analyses). Bold print indicates 95% CrIs excluding no effect. NA=not available.

## 7.7 **24-hour diastolic blood pressure**

### 7.7.1 Network plot

- Individual pharmacological and device-based interventions

The lines link treatments that were directly compared in trials. The thickness of the lines corresponds to the number of trials evaluating the comparison. The size of the circles (nodes) corresponds to the number of participants assigned to the treatment. RDN=renal denervation.

- Pharmacological and device-based intervention classes

The lines link treatments that were directly compared in trials. The thickness of the lines corresponds to the number of trials evaluating the comparison. The size of the circles (nodes) corresponds to the number of participants assigned to the treatment.

### 7.7.2 Forest-plot of network meta-analysis

- Individual pharmacological and device-based interventions

Effect sizes are from the network meta-analysis. Treatments are ordered accordingly to the mean difference. The reference is the baseline treatment employed in each included trial. CrI=credible intervals. DBP=diastolic blood pressure. MD=mean difference. RDN=renal denervation.

- Pharmacological and device-based intervention classes

Effect sizes are from the network meta-analysis. Treatments are ordered accordingly to the mean difference. The reference is the baseline treatment employed in each included trial. CrI=credible intervals. DBP=diastolic blood pressure. MD=mean difference.

### 7.7.4 Forest-plot of pairwise meta-analysis

- Individual pharmacological and device-based interventions

Treatment comparisons are presented in alphabetical order. Summary effect size is calculated using pairwise meta-analysis of all studies in a specific comparison. CI=confidence interval. DBP=diastolic blood pressure. MD=mean difference. RDN=renal denervation.

- Pharmacological and device-based intervention classes

Treatment comparisons are presented in alphabetical order. Summary effect size is calculated using pairwise meta-analysis of all studies in a specific comparison. CI=confidence interval. DBP=diastolic blood pressure. MD=mean difference. RDN=renal denervation.

### 7.7.5 League table

- Individual pharmacological and device-based interventions

| **Baseline treatment** | 2.34 (-3.18 to 7.87) | **2.60** **(0.81 to 4.40)** | **4.75** **(2.15 to 7.35)** | 0.66 (-2.50 to 3.81) |
| --- | --- | --- | --- | --- |
| NA | **Clonidine** | 0.26 (-5.56 to 5.81) | 2.40 (-2.57 to 7.29) | -1.69 (-8.44 to 4.49) |
| **2.24** **(0.98 to 3.51)** | NA | **Radiofrequency**  **RDN** | 2.15 (-0.44 to 5.05) | -1.95 (-5.75 to 1.57) |
| **5.27** **(2.45 to 8.09)** | 2.40 (-0.25 to 5.05) | 1.48 (-3.08 to 6.05) | **Spironolactone** | **-4.09** **(-8.51 to -0.20)** |
| 0.63 (-1.35 to 2.62) | NA | NA | NA | **Ultrasound**  **RDN** |

Treatments are in alphabetical order. In the upper right triangle are presented results from the network meta-analysis. In the lower left triangle are presented results from pairwise meta-analyses (direct comparisons). Each cell provides mean difference and the corresponding 95% CrI (or CI for pairwise meta-analyses). Bold print indicates 95% CrIs excluding no effect. NA=not available. RDN=renal denervation.

- Pharmacological and device-based intervention classes

| **Baseline treatment** | **4.57** **(2.09 to 7.06)** | **2.15** **(0.65 to 3.66)** | 2.17 (-3.15 to 7.50) |
| --- | --- | --- | --- |
| **5.27** **(2.45 to 8.09)** | **Mineralocorticoid receptor antagonists** | -2.42 (-5.21 to 0.03) | -2.40 (-7.25 to 2.38) |
| **1.83** **(0.82 to 2.85)** | -1.48 (-6.05 to 3.08) | **Renal denervation** | 0.02 (-5.22 to 5.68) |
| NA | -2.40 (-5.05 to 0.25) | NA | **α-agonists** |

Treatments are in alphabetical order. In the upper right triangle are presented results from the network meta-analysis. In the lower left triangle are presented results from pairwise meta-analyses (direct comparisons). Each cell provides mean difference and the corresponding 95% CrI (or CI for pairwise meta-analyses). Bold print indicates 95% CrIs excluding no effect. NA=not available.

## 7.9 **Daytime systolic blood pressure**

### 7.9.1 Network plot

- Individual pharmacological and device-based interventions

The lines link treatments that were directly compared in trials. The thickness of the lines corresponds to the number of trials evaluating the comparison. The size of the circles (nodes) corresponds to the number of participants assigned to the treatment. RDN=renal denervation.

- Pharmacological and device-based intervention classes

The lines link treatments that were directly compared in trials. The thickness of the lines corresponds to the number of trials evaluating the comparison. The size of the circles (nodes) corresponds to the number of participants assigned to the treatment.

### 7.9.2 Forest-plot of network meta-analysis

- Individual pharmacological and device-based interventions

Effect sizes are from the network meta-analysis. Treatments are ordered accordingly to the mean difference. The reference is the baseline treatment employed in each included trial. CrI=credible intervals. MD=mean difference. RDN=renal denervation. SBP=systolic blood pressure.

- Pharmacological and device-based intervention classes

Effect sizes are from the network meta-analysis. Treatments are ordered accordingly to the mean difference. The reference is the baseline treatment employed in each included trial. CrI=credible intervals. MD=mean difference. SBP=systolic blood pressure.

### 7.9.4 Forest-plot of pairwise meta-analysis

- Individual pharmacological and device-based interventions

Treatment comparisons are presented in alphabetical order. Summary effect size is calculated using pairwise meta-analysis of all studies in a specific comparison. CI=confidence interval. DBP=diastolic blood pressure. MD=mean difference. RDN=renal denervation.

- Pharmacological and device-based intervention classes

Treatment comparisons are presented in alphabetical order. Summary effect size is calculated using pairwise meta-analysis of all studies in a specific comparison. CI=confidence interval. DBP=diastolic blood pressure. MD=mean difference.

### 7.9.5 League table

- Individual pharmacological and device-based interventions

| **Baseline treatment** | 6.02 (-2.84 to 14.87) | **8.38** **(0.07 to 16.69)** | **4.27** **(1.24 to 7.29)** | **9.75** **(5.74 to 13.77)** | 2.56 (-3.35 to 8.47) |
| --- | --- | --- | --- | --- | --- |
| NA | **Clonidine** | 2.36 (-10.03 to 14.55) | -1.75 (-11.21 to 7.41) | 3.74 (-4.37 to 11.78) | -3.46 (-14.30 to 7.46) |
| **8.40** **(3.12 to 13.68)** | NA | **Darusentan** | -4.11 (-13.13 to 4.89) | 1.37 (-7.95 to 10.86) | -5.82 (-16.09 to 4.43) |
| **4.11** **(1.08 to 7.14)** | NA | NA | **Radiofrequency**  **RDN** | **5.49** **(1.14 to 10.15)** | -1.71 (-8.38 to 5.11) |
| **9.94** **(7.25 to 12.62)** | 3.70 (-0.86 to 8.26) | NA | 6.32 (-4.39 to 17.02) | **Spironolactone** | -7.19 (-14.47 to 0.07) |
| 2.58 (-1.21 to 6.36) | NA | NA | NA | NA | **Ultrasound**  **RDN** |

Treatments are in alphabetical order. In the upper right triangle are presented results from the network meta-analysis. In the lower left triangle are presented results from pairwise meta-analyses (direct comparisons). Each cell provides mean difference and the corresponding 95% CrI (or CI for pairwise meta-analyses). Bold print indicates 95% CrIs excluding no effect. NA=not available. RDN=renal denervation.

- Pharmacological and device-based intervention classes

| **Baseline treatment** | **8.41** **(0.17 to 16.66)** | **9.64** **(5.72 to 13.55)** | **3.92** **(1.28 to 6.56)** | 5.95 (-2.69 to 14.59) |
| --- | --- | --- | --- | --- |
| **8.40** **(3.12 to 13.68)** | **Endothelin receptor antagonists** | 1.23 (-7.78 to 10.60) | -4.50 (-13.20 to 4.20) | -2.46 (-14.35 to 9.80) |
| **9.94** **(7.25 to 12.62)** | NA | **Mineralocorticoid receptor antagonists** | **-5.72** **(-10.19 to -1.41)** | -3.69 (-11.51 to 4.24) |
| **3.77** **(1.31 to 6.23)** | NA | -6.32 (-17.02 to 4.39) | **Renal denervation** | 2.03 (-6.78 to 11.09) |
| NA | NA | -3.70 (-8.26 to 0.86) | NA | **α-agonists** |

Treatments are in alphabetical order. In the upper right triangle are presented results from the network meta-analysis. In the lower left triangle are presented results from pairwise meta-analyses (direct comparisons). Each cell provides mean difference and the corresponding 95% CrI (or CI for pairwise meta-analyses). Bold print indicates 95% CrIs excluding no effect. NA=not available.

## 7.11 **Daytime diastolic blood pressure**

### 7.11.1 Network plot

- Individual pharmacological and device-based interventions

The lines link treatments that were directly compared in trials. The thickness of the lines corresponds to the number of trials evaluating the comparison. The size of the circles (nodes) corresponds to the number of participants assigned to the treatment. RDN=renal denervation.

- Pharmacological and device-based intervention classes

The lines link treatments that were directly compared in trials. The thickness of the lines corresponds to the number of trials evaluating the comparison. The size of the circles (nodes) corresponds to the number of participants assigned to the treatment.

### 7.11.2 Forest-plot of network meta-analysis

- Individual pharmacological and device-based interventions

Effect sizes are from the network meta-analysis. Treatments are ordered accordingly to the mean difference. The reference is the baseline treatment employed in each included trial. CrI=credible intervals. DBP=diastolic blood pressure. MD=mean difference. RDN=renal denervation.

- Pharmacological and device-based intervention classes

Effect sizes are from the network meta-analysis. Treatments are ordered accordingly to the mean difference. The reference is the baseline treatment employed in each included trial. CrI=credible intervals. DBP=diastolic blood pressure. MD=mean difference.

### 7.11.4 Forest-plot of pairwise meta-analysis

- Individual pharmacological and device-based interventions

Treatment comparisons are presented in alphabetical order. Summary effect size is calculated using pairwise meta-analysis of all studies in a specific comparison. CI=confidence interval. DBP=diastolic blood pressure. MD=mean difference. RDN=renal denervation.

- Pharmacological and device-based intervention classes

Treatment comparisons are presented in alphabetical order. Summary effect size is calculated using pairwise meta-analysis of all studies in a specific comparison. CI=confidence interval. DBP=diastolic blood pressure. MD=mean difference.

### 7.11.5 League table

- Individual pharmacological and device-based interventions

| **Baseline treatment** | 1.69 (-3.84 to 7.23) | **1.90** **(0.05 to 3.76)** | **4.28** **(1.69 to 6.86)** | 0.97 (-2.59 to 4.53) |
| --- | --- | --- | --- | --- |
| NA | **Clonidine** | 0.21 (-5.79 to 5.77) | 2.58 (-2.41 to 7.53) | -0.72 (-7.89 to 5.74) |
| **1.74** **(0.31 to 3.17)** | NA | **Radiofrequency**  **RDN** | 2.38 (-0.30 to 5.38) | -0.93 (-5.17 to 3.17) |
| **4.46** **(2.08 to 6.83)** | 2.60 (-0.27 to 5.47) | 1.97 (-2.89 to 6.83) | **Spironolactone** | -3.31 (-8.03 to 0.96) |
| 0.97 (-1.26 to 3.19) | NA | NA | NA | **Ultrasound**  **RDN** |

Treatments are in alphabetical order. In the upper right triangle are presented results from the network meta-analysis. In the lower left triangle are presented results from pairwise meta-analyses (direct comparisons). Each cell provides mean difference and the corresponding 95% CrI (or CI for pairwise meta-analyses). Bold print indicates 95% CrIs excluding no effect. NA=not available. RDN=renal denervation.

- Pharmacological and device-based intervention classes

| **Baseline treatment** | **4.16** **(1.75 to 6.57)** | **1.71** **(0.18 to 3.24)** | 1.56 (-3.60 to 6.71) |
| --- | --- | --- | --- |
| **4.46** **(2.08 to 6.83)** | **Mineralocorticoid receptor antagonists** | **-2.45** **(-5.21 to -0.03)** | -2.60 (-7.35 to 2.06) |
| **1.56 (0.42 to 2.70)** | 1.97 (-2.89 to 6.83) | **Renal denervation** | -0.15 (-5.34 to 5.41) |
| NA | -2.60 (-5.47 to 0.27) | NA | **α-agonists** |

Treatments are in alphabetical order. In the upper right triangle are presented results from the network meta-analysis. In the lower left triangle are presented results from pairwise meta-analyses (direct comparisons). Each cell provides mean difference and the corresponding 95% CrI (or CI for pairwise meta-analyses). Bold print indicates 95% CrIs excluding no effect. NA=not available.

## 7.13 **Nighttime systolic blood pressure**

### 7.13.1 Network plot

- Individual pharmacological and device-based interventions

The lines link treatments that were directly compared in trials. The thickness of the lines corresponds to the number of trials evaluating the comparison. The size of the circles (nodes) corresponds to the number of participants assigned to the treatment. RDN=renal denervation.

- Pharmacological and device-based intervention classes

The lines link treatments that were directly compared in trials. The thickness of the lines corresponds to the number of trials evaluating the comparison. The size of the circles (nodes) corresponds to the number of participants assigned to the treatment.

### 7.13.2 Forest-plot of network meta-analysis

- Individual pharmacological and device-based interventions

Effect sizes are from the network meta-analysis. Treatments are ordered accordingly to the mean difference. The reference is the baseline treatment employed in each included trial. CrI=credible intervals. MD=mean difference. RDN=renal denervation. SBP=systolic blood pressure.

- Pharmacological and device-based intervention classes

Effect sizes are from the network meta-analysis. Treatments are ordered accordingly to the mean difference. The reference is the baseline treatment employed in each included trial. CrI=credible intervals. MD=mean difference. SBP=systolic blood pressure.

### 7.13.4 Forest-plot of pairwise meta-analysis

- Individual pharmacological and device-based interventions

Treatment comparisons are presented in alphabetical order. Summary effect size is calculated using pairwise meta-analysis of all studies in a specific comparison. CI=confidence interval. DBP=diastolic blood pressure. MD=mean difference. RDN=renal denervation.

- Pharmacological and device-based intervention classes

Treatment comparisons are presented in alphabetical order. Summary effect size is calculated using pairwise meta-analysis of all studies in a specific comparison. CI=confidence interval. DBP=diastolic blood pressure. MD=mean difference.

### 7.13.5 League table

- Individual pharmacological and device-based interventions

| **Baseline treatment** | 8.35 (-1.49 to 18.19) | 8.57 (-0.01 to 17.14) | **4.31** **(1.21 to 7.41)** | **10.99** **(6.53 to 15.45)** | 1.45 (-4.93 to 7.83) |
| --- | --- | --- | --- | --- | --- |
| NA | **Clonidine** | 0.22 (-13.22 to 13.32) | -4.04 (-14.41 to 5.97) | 2.64 (-6.36 to 11.56) | -6.90 (-19.03 to 4.93) |
| **8.60** **(2.62 to 14.58)** | NA | **Darusentan** | -4.26 (-13.49 to 4.89) | 2.42 (-7.23 to 12.26) | -7.12 (-17.96 to 3.89) |
| **3.79** **(0.96 to 6.62)** | NA | NA | **Radiofrequency**  **RDN** | **6.68** **(1.92 to 11.76)** | -2.86 (-9.87 to 4.54) |
| **12.41** **(8.76 to 16.07)** | 2.60  (-3.69 to 8.89) | NA | 3.14 (-4.93 to 11.20) | **Spironolactone** | **-9.54** **(-17.40 to -1.68)** |
| 1.54 (-3.00 to 6.07) | NA | NA | NA | NA | **Ultrasound**  **RDN** |

Treatments are in alphabetical order. In the upper right triangle are presented results from the network meta-analysis. In the lower left triangle are presented results from pairwise meta-analyses (direct comparisons). Each cell provides mean difference and the corresponding 95% CrI (or CI for pairwise meta-analyses). Bold print indicates 95% CrIs excluding no effect. NA=not available. RDN=renal denervation.

- Pharmacological and device-based intervention classes

| **Baseline treatment** | 8.62 (-0.02 to 17.25) | **10.86** **(6.46 to 15.25)** | **3.79** **(1.04 to 6.54)** | 8.20 (-1.65 to 18.05) |
| --- | --- | --- | --- | --- |
| **8.60** **(2.62 to 14.58)** | **Endothelin receptor antagonists** | 2.24 (-7.61 to 12.16) | -4.83 (-14.20 to 4.21) | -0.42 (-13.75 to 13.01) |
| **12.41** **(8.76 to 16.07)** | NA | **Mineralocorticoid receptor antagonists** | **-7.06** **(-11.92 to -2.42)** | -2.66 (-11.57 to 6.29) |
| **3.39** **(0.95 to 5.82)** | NA | -3.14 (-11.20 to 4.93) | **Renal denervation** | 4.41 (-5.63 to 14.62) |
| NA | NA | -2.60 (-8.89 to 3.69) | NA | **α-agonists** |

Treatments are in alphabetical order. In the upper right triangle are presented results from the network meta-analysis. In the lower left triangle are presented results from pairwise meta-analyses (direct comparisons). Each cell provides mean difference and the corresponding 95% CrI (or CI for pairwise meta-analyses). Bold print indicates 95% CrIs excluding no effect. NA=not available.

## 7.15 **Nighttime diastolic blood pressure**

### 7.15.1 Network plot

- Individual pharmacological and device-based interventions

The lines link treatments that were directly compared in trials. The thickness of the lines corresponds to the number of trials evaluating the comparison. The size of the circles (nodes) corresponds to the number of participants assigned to the treatment. RDN=renal denervation.

- Pharmacological and device-based intervention classes

The lines link treatments that were directly compared in trials. The thickness of the lines corresponds to the number of trials evaluating the comparison. The size of the circles (nodes) corresponds to the number of participants assigned to the treatment.

### 7.15.2 Forest-plot of network meta-analysis

- Individual pharmacological and device-based interventions

Effect sizes are from the network meta-analysis. Treatments are ordered accordingly to the mean difference. The reference is the baseline treatment employed in each included trial. CrI=credible intervals. DBP=diastolic blood pressure. MD=mean difference. RDN=renal denervation.

- Pharmacological and device-based intervention classes

Effect sizes are from the network meta-analysis. Treatments are ordered accordingly to the mean difference. The reference is the baseline treatment employed in each included trial. CrI=credible intervals. DBP=diastolic blood pressure. MD=mean difference.

### 7.15.4 Forest-plot of pairwise meta-analysis

- Individual pharmacological and device-based interventions

Treatment comparisons are presented in alphabetical order. Summary effect size is calculated using pairwise meta-analysis of all studies in a specific comparison. CI=confidence interval. DBP=diastolic blood pressure. MD=mean difference. RDN=renal denervation.

- Pharmacological and device-based intervention classes

Treatment comparisons are presented in alphabetical order. Summary effect size is calculated using pairwise meta-analysis of all studies in a specific comparison. CI=confidence interval. DBP=diastolic blood pressure. MD=mean difference.

### 7.15.5 League table

- Individual pharmacological and device-based interventions

| **Baseline treatment** | 3.47 (-4.88 to 11.81) | 1.93 (-0.87 to 4.73) | **5.88** **(2.05 to 9.71)** | 0.70 (-4.65 to 6.05) |
| --- | --- | --- | --- | --- |
| NA | **Clonidine** | -1.53 (-10.29 to 6.97) | 2.41 (-4.99 to 10.05) | -2.77 (-13.07 to 7.25) |
| 1.46 (-0.19 to 3.11) | NA | **Radiofrequency**  **RDN** | 3.95 (-0.08 to 8.26) | -1.24 (-7.48 to 4.96) |
| **7.83** **(2.92 to 12.75)** | 2.40 (-0.92 to 5.72) | 0.09 (-3.89 to 4.06) | **Spironolactone** | -5.18 (-12.00 to 1.34) |
| 0.59 (-2.10 to 3.29) | NA | NA | NA | **Ultrasound**  **RDN** |

Treatments are in alphabetical order. In the upper right triangle are presented results from the network meta-analysis. In the lower left triangle are presented results from pairwise meta-analyses (direct comparisons). Each cell provides mean difference and the corresponding 95% CrI (or CI for pairwise meta-analyses). Bold print indicates 95% CrIs excluding no effect. NA=not available. RDN=renal denervation.

- Pharmacological and device-based intervention classes

| **Baseline treatment** | **5.74** **(2.04 to 9.44)** | 1.68 (-0.67 to 4.04) | 3.36 (-4.68 to 11.40) |
| --- | --- | --- | --- |
| **7.83 (2.92 to 12.75)** | **Mineralocorticoid receptor antagonists** | **-4.06** **(-8.14 to -0.28)** | -2.38 (-9.84 to 4.94) |
| 1.33 (-0.06 to 2.72) | -0.09 (-4.06 to 3.89) | **Renal denervation** | 1.68 (-6.45 to 10.16) |
| NA | -2.40 (-5.72 to 0.92) | NA | **α-agonists** |

Treatments are in alphabetical order. In the upper right triangle are presented results from the network meta-analysis. In the lower left triangle are presented results from pairwise meta-analyses (direct comparisons). Each cell provides mean difference and the corresponding 95% CrI (or CI for pairwise meta-analyses). Bold print indicates 95% CrIs excluding no effect. NA=not available.

# 9. **Uncontrolled hypertension**

For each outcome of interest, the following analyses and graphical displays were presented for both individual pharmacological and device-based interventionss and their corresponding classes, within the uncontrolled hypertension subgroup:

- Network plot

- Forest-plot of network meta-analysis (baseline treatment as reference)

- Forest-plot of pairwise meta-analyses

- League table

## 9.2 **Office systolic blood pressure**

### 9.2.1 Network plot

- Individual pharmacological and device-based interventions

The lines link treatments that were directly compared in trials. The thickness of the lines corresponds to the number of trials evaluating the comparison. The size of the circles (nodes) corresponds to the number of participants assigned to the treatment. RDN=renal denervation

- Pharmacological and device-based intervention classes

The lines link treatments that were directly compared in trials. The thickness of the lines corresponds to the number of trials evaluating the comparison. The size of the circles (nodes) corresponds to the number of participants assigned to the treatment.

### 9.2.3 Forest-plot of pairwise meta-analysis

- Individual pharmacological and device-based interventions

Treatment comparisons are presented in alphabetical order. Summary effect size is calculated using pairwise meta-analysis of all studies in a specific comparison. CI=confidence interval. DBP=diastolic blood pressure. MD=mean difference. RDN=renal denervation.

- Pharmacological and device-based intervention classes

Treatment comparisons are presented in alphabetical order. Summary effect size is calculated using pairwise meta-analysis of all studies in a specific comparison. CI=confidence interval. DBP=diastolic blood pressure. MD=mean difference.

### 9.2.4 League table

- Pharmacological and device-based intervention classes

| **Aldosterone synthase inhibitors** | **-7.77 (-12.22 to -3.31)** | -2.12 (-9.35 to 5.03) | 2.88 (-3.75 to 9.49) | -1.21 (-6.65 to 4.14) | -3.52 (-12.32 to 5.21) |
| --- | --- | --- | --- | --- | --- |
| **-8.45 (-10.16 to -6.73)** | **Baseline treatment** | 5.64 (-0.03 to 11.31) | **10.64 (5.68 to 15.60)** | **6.56 (3.57 to 9.54)** | 4.24 (-3.35 to 11.84) |
| NA | **5.05 (2.45 to 7.66)** | **Endothelin receptor antagonists** | 5.00 (-2.09 to 12.08) | 0.91 (-5.41 to 7.31) | -1.40 (-9.03 to 6.26) |
| NA | **12.56 (9.17 to 15.95)** | NA | **Mineralocorticoid receptor antagonists** | -4.09 (-9.43 to 1.30) | -6.40 (-14.17 to 1.46) |
| NA | **6.44 (2.10 to 10.79)** | NA | 0.64 (-6.27 to 4.99) | **Renal**  **denervation** | -2.31 (-10.35 to 5.77) |
| NA | NA | NA | -1.40 (-7.94 to 5.14) | NA | **α-agonists** |

Treatments are in alphabetical order. In the upper right triangle are presented results from the network meta-analysis. In the lower left triangle are presented results from pairwise meta-analyses (direct comparisons). Each cell provides mean difference and the corresponding 95% CrI (or CI for pairwise meta-analyses). Bold print indicates 95% CrIs excluding no effect. NA=not available.

### 9.2.6 Network meta-regression analyses

For the primary outcome, we presented the forest-plot of the following adjusted network meta-analyses (baseline treatment as reference).

We did not present the network plot, as each study reported the covariates used in the analyses; therefore, the network of comparisons remains identical to that of the main analysis.

The network meta-regression analysis by body mass index was conducted on a post-hoc basis, following a Reviewer’s comment.

**Mean Age**

- Individual pharmacological and device-based interventions

Effect sizes are from the network meta-analysis. Treatments are ordered accordingly to the mean difference. The reference is the baseline treatment employed in each included trial. CrI=credible intervals. MD=mean difference. RDN=renal denervation. SBP=systolic blood pressure.

- Pharmacological and device-based intervention classes

Effect sizes are from the network meta-analysis. Treatments are ordered accordingly to the mean difference. The reference is the baseline treatment employed in each included trial. CrI=credible intervals. MD=mean difference. SBP=systolic blood pressure.

**Proportion of females**

- Individual pharmacological and device-based interventions

Effect sizes are from the network meta-analysis. Treatments are ordered accordingly to the mean difference. The reference is the baseline treatment employed in each included trial. CrI=credible intervals. MD=mean difference. RDN=renal denervation. SBP=systolic blood pressure.

- Pharmacological and device-based intervention classes

Effect sizes are from the network meta-analysis. Treatments are ordered accordingly to the mean difference. The reference is the baseline treatment employed in each included trial. CrI=credible intervals. MD=mean difference. SBP=systolic blood pressure.

**Baseline systolic blood pressure**

- Individual pharmacological and device-based interventions

Effect sizes are from the network meta-analysis. Treatments are ordered accordingly to the mean difference. The reference is the baseline treatment employed in each included trial. CrI=credible intervals. MD=mean difference. RDN=renal denervation. SBP=systolic blood pressure.

- Pharmacological and device-based intervention classes

Effect sizes are from the network meta-analysis. Treatments are ordered accordingly to the mean difference. The reference is the baseline treatment employed in each included trial. CrI=credible intervals. MD=mean difference. SBP=systolic blood pressure.

**Year of study publication**

- Individual pharmacological and device-based interventions

Effect sizes are from the network meta-analysis. Treatments are ordered accordingly to the mean difference. The reference is the baseline treatment employed in each included trial. CrI=credible intervals. MD=mean difference. RDN=renal denervation. SBP=systolic blood pressure.

- Pharmacological and device-based intervention classes

Effect sizes are from the network meta-analysis. Treatments are ordered accordingly to the mean difference. The reference is the baseline treatment employed in each included trial. CrI=credible intervals. MD=mean difference. SBP=systolic blood pressure.

**Body mass index**

- Individual pharmacological and device-based interventions

Effect sizes are from the network meta-analysis. Treatments are ordered accordingly to the mean difference. The reference is the baseline treatment employed in each included trial. CrI=credible intervals. MD=mean difference. RDN=renal denervation. SBP=systolic blood pressure.

- Pharmacological and device-based intervention classes

Effect sizes are from the network meta-analysis. Treatments are ordered accordingly to the mean difference. The reference is the baseline treatment employed in each included trial. CrI=credible intervals. MD=mean difference. SBP=systolic blood pressure.

### 9.4.1 Sensitivity analyses

For the primary outcome, we planned the following subgroup analyses:

- Including studies comparing a treatment with placebo or sham procedure

- Excluding studies with high risk of bias

**Placebo/Sham studies**

- Individual pharmacological and device-based interventions

Effect sizes are from the network meta-analysis. Treatments are ordered accordingly to the mean difference. The reference is the baseline treatment employed in each included trial. CrI=credible intervals. MD=mean difference. RDN=renal denervation. SBP=systolic blood pressure.

- Pharmacological and device-based intervention classes

Effect sizes are from the network meta-analysis. Treatments are ordered accordingly to the mean difference. The reference is the baseline treatment employed in each included trial. CrI=credible intervals. MD=mean difference. SBP=systolic blood pressure.

**Excluding studies with high risk of bias**

- Individual pharmacological and device-based interventions

Effect sizes are from the network meta-analysis. Treatments are ordered accordingly to the mean difference. The reference is the baseline treatment employed in each included trial. CrI=credible intervals. MD=mean difference. RDN=renal denervation. SBP=systolic blood pressure.

- Pharmacological and device-based intervention classes

Effect sizes are from the network meta-analysis. Treatments are ordered accordingly to the mean difference. The reference is the baseline treatment employed in each included trial. CrI=credible intervals. MD=mean difference. SBP=systolic blood pressure.

**Excluding studies using MRAs as baseline treatment**

- Individual pharmacological and device-based interventions

Effect sizes are from the network meta-analysis. Treatments are ordered accordingly to the mean difference. The reference is the baseline treatment employed in each included trial. CrI=credible intervals. MD=mean difference. RDN=renal denervation. SBP=systolic blood pressure.

- Pharmacological and device-based intervention classes

Effect sizes are from the network meta-analysis. Treatments are ordered accordingly to the mean difference. The reference is the baseline treatment employed in each included trial. CrI=credible intervals. MD=mean difference. SBP=systolic blood pressure.

## 9.5 **Office diastolic blood pressure**

### 9.5.1 Network plot

- Individual pharmacological and device-based interventions

The lines link treatments that were directly compared in trials. The thickness of the lines corresponds to the number of trials evaluating the comparison. The size of the circles (nodes) corresponds to the number of participants assigned to the treatment. RDN=renal denervation.

- Pharmacological and device-based intervention classes

The lines link treatments that were directly compared in trials. The thickness of the lines corresponds to the number of trials evaluating the comparison. The size of the circles (nodes) corresponds to the number of participants assigned to the treatment.

### 9.5.2 Forest-plot of network meta-analysis

- Individual pharmacological and device-based interventions

Effect sizes are from the network meta-analysis. Treatments are ordered accordingly to the mean difference. The reference is the baseline treatment employed in each included trial. CrI=credible intervals. DBP=diastolic blood pressure. MD=mean difference. RDN=renal denervation.

- Pharmacological and device-based intervention classes

Effect sizes are from the network meta-analysis. Treatments are ordered accordingly to the mean difference. The reference is the baseline treatment employed in each included trial. CrI=credible intervals. DBP=diastolic blood pressure. MD=mean difference.

### 9.5.4 Forest-plot of pairwise meta-analysis

- Individual pharmacological and device-based interventions

Treatment comparisons are presented in alphabetical order. Summary effect size is calculated using pairwise meta-analysis of all studies in a specific comparison. CI=confidence interval. DBP=diastolic blood pressure. MD=mean difference. RDN=renal denervation.

- Pharmacological and device-based intervention classes

Treatment comparisons are presented in alphabetical order. Summary effect size is calculated using pairwise meta-analysis of all studies in a specific comparison. CI=confidence interval. DBP=diastolic blood pressure. MD=mean difference.

### 9.5.6 League table

- Individual pharmacological and device-based interventions

| **Alcohol RDN** | -1.30 (-8.91 to 6.30) | 5.58 (-3.94 to 15.04) | 6.32 (-5.75 to 18.52) | 5.99 (-4.96 to 16.77) | 4.08 (-5.35 to 13.63) | 5.88 (-2.32 to 14.04) | 7.61 (-0.98 to 16.47) | 2.00 (-7.43 to 11.31) |
| --- | --- | --- | --- | --- | --- | --- | --- | --- |
| 1.30 (-0.76 to 3.36) | **Baseline treatment** | 4.27 (-1.17 to 9.72) | 5.01 (-4.28 to 14.30) | 4.69 (-2.99 to 12.36) | 2.78 (-2.88 to 8.45) | **4.58 (1.85 to 7.30)** | **6.31 (2.08 to 10.54)** | 0.70 (-4.49 to 5.89) |
| NA | **3.98 (2.58 to 5.38)** | **Baxdrostat** | 0.74 (-10.07 to 11.74) | 0.41 (-9.16 to 9.93) | -1.49 (-9.58 to 6.46) | 0.30 (-5.86 to 6.48) | 2.04 (-4.80 to 9.06) | -3.58 (-11.26 to 4.01) |
| NA | NA | NA | **Clonidine** | -0.32 (-12.46 to 11.73) | -2.23 (-13.46 to 8.60) | -0.43 (-9.92 to 9.03) | 1.30 (-7.00 to 9.70) | -4.31 (-15.17 to 6.41) |
| NA | **4.68 (2.85 to 6.51)** | NA | NA | **Darusentan** | -1.91 (-11.68 to 7.74) | -0.11 (-8.35 to 8.05) | 1.62 (-7.10 to 10.45) | -3.99 (-13.59 to 5.41) |
| NA | **3.02 (0.87 to 5.16)** | NA | NA | NA | **Lorundrostat** | 1.79 (-4.52 to 8.15) | 3.53 (-3.62 to 10.85) | -2.08 (-9.88 to 5.81) |
| NA | **4.45 (1.48 to 7.42)** | NA | NA | NA | NA | **Radiofrequency RDN** | 1.73 (-2.78 to 6.22) | -3.88 (-9.83 to 2.01) |
| NA | **6.32 (2.76 to 9.89)** | NA | 1.30 (-2.48 to 5.08) | NA | NA | -0.41 (-3.74 to 2.92) | **Spironolactone** | -5.61 (-12.49 to 1.05) |
| NA | 1.01 (-2.53 to 4.55) | NA | NA | NA | NA | NA | NA | **Ultrasound RDN** |

Treatments are in alphabetical order. In the upper right triangle are presented results from the network meta-analysis. In the lower left triangle are presented results from pairwise meta-analyses (direct comparisons). Each cell provides mean difference and the corresponding 95% CrI (or CI for pairwise meta-analyses). Bold print indicates 95% CrIs excluding no effect. NA=not available. RDN=renal denervation.

- Pharmacological and device-based intervention classes

| **Aldosterone synthase inhibitors** | -3.56 (-7.46 to 0.33) | 1.20 (-7.41 to 9.82) | 2.30 (-3.32 to 8.13) | -0.26 (-4.81 to 4.27) | 1.01 (-8.90 to 11.23) |
| --- | --- | --- | --- | --- | --- |
| **-3.69 (-4.87 to -2.52)** | **Baseline treatment** | 4.76 (-2.81 to 12.32) | **5.87 (1.68 to 10.05)** | **3.30 (1.02 to 5.58)** | 4.57 (-4.65 to 13.80) |
| NA | **4.68 (2.85 to 6.51)** | **Endothelin receptor antagonists** | 1.11 (-7.53 to 9.98) | -1.46 (-9.45 to 6.62) | -0.18 (-12.29 to 11.78) |
| NA | **6.32 (2.76 to 9.89)** | NA | **Mineralocorticoid receptor antagonists** | -2.57 (-6.96 to 1.79) | -1.29 (-9.53 to 7.15) |
| NA | **3.10 (0.68 to 5.52)** | NA | 0.41 (-2.92 to 3.74) | **Renal**  **denervation** | 1.27 (-8.04 to 10.79) |
| NA | NA | NA | -1.30 (-5.08 to 2.48) | NA | **α-agonists** |

Treatments are in alphabetical order. In the upper right triangle are presented results from the network meta-analysis. In the lower left triangle are presented results from pairwise meta-analyses (direct comparisons). Each cell provides mean difference and the corresponding 95% CrI (or CI for pairwise meta-analyses). Bold print indicates 95% CrIs excluding no effect. NA=not available.

## 9.7 **24-hour systolic blood pressure**

### 9.7.1 Network plot

- Individual pharmacological and device-based interventions

The lines link treatments that were directly compared in trials. The thickness of the lines corresponds to the number of trials evaluating the comparison. The size of the circles (nodes) corresponds to the number of participants assigned to the treatment. NA=not available. RDN=renal denervation.

- Pharmacological and device-based intervention classes

The lines link treatments that were directly compared in trials. The thickness of the lines corresponds to the number of trials evaluating the comparison. The size of the circles (nodes) corresponds to the number of participants assigned to the treatment.

### 9.7.2 Forest-plot of network meta-analysis

- Individual pharmacological and device-based interventions

Effect sizes are from the network meta-analysis. Treatments are ordered accordingly to the mean difference. The reference is the baseline treatment employed in each included trial. CrI=credible intervals. MD=mean difference. RDN=renal denervation. SBP=systolic blood pressure.

- Pharmacological and device-based intervention classes

Effect sizes are from the network meta-analysis. Treatments are ordered accordingly to the mean difference. The reference is the baseline treatment employed in each included trial. CrI=credible intervals. MD=mean difference. SBP=systolic blood pressure.

### 9.7.4 Forest-plot of pairwise meta-analysis

- Individual pharmacological and device-based interventions

Treatment comparisons are presented in alphabetical order. Summary effect size is calculated using pairwise meta-analysis of all studies in a specific comparison. CI=confidence interval. DBP=diastolic blood pressure. MD=mean difference. RDN=renal denervation.

- Pharmacological and device-based intervention classes

Treatment comparisons are presented in alphabetical order. Summary effect size is calculated using pairwise meta-analysis of all studies in a specific comparison. CI=confidence interval. DBP=diastolic blood pressure. MD=mean difference.

### 9.7.5 League table

- Individual pharmacological and device-based interventions

| **Alcohol RDN** | 3.19 (-3.46 to 9.83) | **11.41 (2.76 to 20.07)** | 1.94 (-8.89 to 12.85) | 3.82 (-5.83 to 13.37) | -1.16 (-10.64 to 8.22) | 4.04 (-5.91 to 13.90) | 1.03 (-6.22 to 8.20) | 6.43 (-1.32 to 14.18) | -1.52 (-9.81 to 6.86) |
| --- | --- | --- | --- | --- | --- | --- | --- | --- | --- |
| **-3.20 (-6.18 to -0.22)** | **Baseline treatment** | **14.60 (9.28 to 19.92)** | 5.13 (-3.28 to 13.53) | **7.01 (0.46 to 13.56)** | 2.02 (-4.40 to 8.45) | **7.23 (0.03 to 14.42)** | **4.22 (1.89 to 6.54)** | **9.62 (5.81 to 13.44)** | 1.67 (-3.14 to 6.48) |
| NA | **14.31 (11.48 to 17.14)** | **Baxdrostat** | -9.47 (-19.50 to 0.65) | -7.59 (-16.24 to 0.98) | **-12.57 (-21.09 to -4.16)** | -7.37 (-16.56 to 1.77) | **-10.38 (-16.41 to -4.51)** | -4.98 (-11.52 to 1.69) | **-12.93 (-20.12 to -5.70)** |
| NA | NA | NA | **Clonidine** | 1.88 (-8.96 to 12.61) | -3.10 (-13.82 to 7.69) | 2.10 (-9.01 to 13.36) | -0.91 (-9.59 to 7.70) | 4.50 (-3.04 to 12.16) | -3.46 (-13.16 to 6.28) |
| NA | **7.00 (4.41 to 9.59)** | NA | NA | **Darusentan** | -4.98 (-11.33 to 1.37) | 0.22 (-9.72 to 10.08) | -2.79 (-10.03 to 4.24) | 2.62 (-5.03 to 10.43) | -5.34 (-13.64 to 2.82) |
| NA | NA | NA | NA | NA | **Guanfacine** | 5.20 (-4.71 to 14.99) | 2.19 (-4.84 to 9.15) | **7.60 (0.07 to 15.24)** | -0.36 (-8.59 to 7.74) |
| NA | **7.20 (3.04 to 11.36)** | NA | NA | NA | NA | **Lorundrostat** | -3.01 (-10.62 to 4.63) | 2.40 (-5.80 to 10.73) | -5.56 (-14.29 to 3.14) |
| NA | **4.10 (1.79 to 6.42)** | NA | NA | NA | NA | NA | **Radiofrequency RDN** | **5.41 (1.36 to 9.65)** | -2.55 (-7.97 to 3.00) |
| NA | **9.98 (7.29 to 12.68)** | NA | 4.50 (-0.06 to 9.06) | NA | NA | NA | 6.27 (-3.99 to 16.54) | **Spironolactone** | **-7.95 (-14.11 to -1.81)** |
| NA | 1.67 (-1.67 to 5.01) | NA | NA | NA | NA | NA | NA | NA | **Ultrasound RDN** |

Treatments are in alphabetical order. In the upper right triangle are presented results from the network meta-analysis. In the lower left triangle are presented results from pairwise meta-analyses (direct comparisons). Each cell provides mean difference and the corresponding 95% CrI (or CI for pairwise meta-analyses). Bold print indicates 95% CrIs excluding no effect. NA=not available. RDN=renal denervation.

- Pharmacological and device-based intervention classes

| **Aldosterone synthase inhibitors** | **-11.99 (-16.21 to -7.77)** | -4.40 (-11.81 to 3.08) | -2.91 (-8.47 to 2.68) | **-8.32 (-13.09 to -3.69)** | **-8.91 (-15.56 to -2.07)** |
| --- | --- | --- | --- | --- | --- |
| **-11.97 (-15.93 to -8.01)** | **Baseline treatment** | **7.59 (1.61 to 13.57)** | **9.09 (5.58 to 12.59)** | **3.67 (1.68 to 5.65)** | 3.08 (-2.01 to 8.17) |
| NA | **7.00 (4.41 to 9.59)** | **Endothelin receptor antagonists** | 1.49 (-5.01 to 8.10) | -3.93 (-10.37 to 2.39) | -4.51 (-10.49 to 1.47) |
| NA | **9.98 (7.29 to 12.68)** | NA | **Mineralocorticoid receptor antagonists** | **-5.42 (-9.29 to -1.62)** | **-6.00 (-11.28 to -0.58)** |
| NA | **3.63 (1.84 to 5.40)** | NA | -6.27 (-16.54 to 3.99) | **Renal**  **denervation** | -0.58 (-5.94 to 5.12) |
| NA | NA | NA | -4.50 (-9.06 to 0.06) | NA | **α-agonists** |

Treatments are in alphabetical order. In the upper right triangle are presented results from the network meta-analysis. In the lower left triangle are presented results from pairwise meta-analyses (direct comparisons). Each cell provides mean difference and the corresponding 95% CrI (or CI for pairwise meta-analyses). Bold print indicates 95% CrIs excluding no effect. NA=not available.

## 9.9 **24-hour diastolic blood pressure**

### 9.9.1 Network plot

- Individual pharmacological and device-based interventions

The lines link treatments that were directly compared in trials. The thickness of the lines corresponds to the number of trials evaluating the comparison. The size of the circles (nodes) corresponds to the number of participants assigned to the treatment. RDN=renal denervation.

- Pharmacological and device-based intervention classes

The lines link treatments that were directly compared in trials. The thickness of the lines corresponds to the number of trials evaluating the comparison. The size of the circles (nodes) corresponds to the number of participants assigned to the treatment.

### 9.9.2 Forest-plot of network meta-analysis

- Individual pharmacological and device-based interventions

Effect sizes are from the network meta-analysis. Treatments are ordered accordingly to the mean difference. The reference is the baseline treatment employed in each included trial. CrI=credible intervals. DBP=diastolic blood pressure. MD=mean difference. RDN=renal denervation.

- Pharmacological and device-based intervention classes

Effect sizes are from the network meta-analysis. Treatments are ordered accordingly to the mean difference. The reference is the baseline treatment employed in each included trial. CrI=credible intervals. DBP=diastolic blood pressure. MD=mean difference.

### 9.9.4 Forest-plot of pairwise meta-analysis

- Individual pharmacological and device-based interventions

Treatment comparisons are presented in alphabetical order. Summary effect size is calculated using pairwise meta-analysis of all studies in a specific comparison. CI=confidence interval. DBP=diastolic blood pressure. MD=mean difference. RDN=renal denervation.

- Pharmacological and device-based intervention classes

Treatment comparisons are presented in alphabetical order. Summary effect size is calculated using pairwise meta-analysis of all studies in a specific comparison. CI=confidence interval. DBP=diastolic blood pressure. MD=mean difference. RDN=renal denervation.

### 9.9.5 League table

- Individual pharmacological and device-based interventions

| **Alcohol RDN** | 3.01 (-2.14 to 8.16) | 3.81 (-3.37 to 11.01) | -0.62 (-8.27 to 7.26) | -0.58 (-6.04 to 4.86) | 1.77 (-3.97 to 7.81) | -2.34 (-8.52 to 3.86) |
| --- | --- | --- | --- | --- | --- | --- |
| **-3.00 (-5.88 to -0.12)** | **Baseline treatment** | **6.82 (2.13 to 11.51)** | 2.39 (-3.29 to 8.07) | **2.43 (0.85 to 4.01)** | **4.79 (2.15 to 7.42)** | 0.67 (-2.56 to 3.90) |
| NA | **6.80 (4.82 to 8.78)** | **Baxdrostat** | -4.43 (-11.77 to 3.38) | -4.39 (-9.43 to 0.73) | -2.04 (-7.32 to 3.77) | **-6.15 (-12.01 to -0.32)** |
| NA | NA | NA | **Clonidine** | 0.04 (-5.92 to 5.68) | 2.40 (-2.71 to 7.54) | -1.72 (-8.50 to 4.70) |
| NA | **2.26 (0.96 to 3.56)** | NA | NA | **Radiofrequency RDN** | 2.35 (-0.27 to 5.26) | -1.76 (-5.42 to 1.85) |
| NA | **5.27 (2.45 to 8.09)** | NA | 2.40 (-0.25 to 5.05) | 1.48 (-3.08 to 6.05) | **Spironolactone** | **-4.11 (-8.46 to -0.05)** |
| NA | 0.63 (-1.35 to 2.62) | NA | NA | NA | NA | **Ultrasound RDN** |

Treatments are in alphabetical order. In the upper right triangle are presented results from the network meta-analysis. In the lower left triangle are presented results from pairwise meta-analyses (direct comparisons). Each cell provides mean difference and the corresponding 95% CrI (or CI for pairwise meta-analyses). Bold print indicates 95% CrIs excluding no effect. NA=not available. RDN=renal denervation.

- Pharmacological and device-based intervention classes

| **Aldosterone synthase inhibitors** | **-6.78 (-11.26 to -2.30)** | -2.13 (-7.21 to 3.24) | -4.61 (-9.37 to 0.15) | -4.56 (-11.51 to 2.80) |
| --- | --- | --- | --- | --- |
| **-6.80 (-8.78 to -4.82)** | **Baseline treatment** | **4.65 (2.17 to 7.13)** | **2.18 (0.88 to 3.47)** | 2.22 (-3.18 to 7.62) |
| NA | **5.27 (2.45 to 8.09)** | **Mineralocorticoid receptor antagonists** | -2.48 (-5.21 to 0.05) | -2.43 (-7.31 to 2.49) |
| NA | **2.02 (0.99 to 3.06)** | -1.48 (-6.05 to 3.08) | **Renal**  **denervation** | 0.05 (-5.32 to 5.77) |
| NA | NA | -2.40 (-5.05 to 0.25) | NA | **α-agonists** |

Treatments are in alphabetical order. In the upper right triangle are presented results from the network meta-analysis. In the lower left triangle are presented results from pairwise meta-analyses (direct comparisons). Each cell provides mean difference and the corresponding 95% CrI (or CI for pairwise meta-analyses). Bold print indicates 95% CrIs excluding no effect. NA=not available.

## 9.11 **Daytime systolic blood pressure**

### 9.11.1 Network plot

- Individual pharmacological and device-based interventions

The lines link treatments that were directly compared in trials. The thickness of the lines corresponds to the number of trials evaluating the comparison. The size of the circles (nodes) corresponds to the number of participants assigned to the treatment. RDN=renal denervation.

- Pharmacological and device-based intervention classes

The lines link treatments that were directly compared in trials. The thickness of the lines corresponds to the number of trials evaluating the comparison. The size of the circles (nodes) corresponds to the number of participants assigned to the treatment.

### 9.11.2 Forest-plot of network meta-analysis

- Individual pharmacological and device-based interventions

Effect sizes are from the network meta-analysis. Treatments are ordered accordingly to the mean difference. The reference is the baseline treatment employed in each included trial. CrI=credible intervals. MD=mean difference. RDN=renal denervation. SBP=systolic blood pressure.

- Pharmacological and device-based intervention classes

Effect sizes are from the network meta-analysis. Treatments are ordered accordingly to the mean difference. The reference is the baseline treatment employed in each included trial. CrI=credible intervals. MD=mean difference. SBP=systolic blood pressure.

### 9.11.4 Forest-plot of pairwise meta-analysis

- Individual pharmacological and device-based interventions

Treatment comparisons are presented in alphabetical order. Summary effect size is calculated using pairwise meta-analysis of all studies in a specific comparison. CI=confidence interval. DBP=diastolic blood pressure. MD=mean difference. RDN=renal denervation.

- Pharmacological and device-based intervention classes

Treatment comparisons are presented in alphabetical order. Summary effect size is calculated using pairwise meta-analysis of all studies in a specific comparison. CI=confidence interval. DBP=diastolic blood pressure. MD=mean difference.

### 9.11.5 League table

- Individual pharmacological and device-based interventions

| **Alcohol RDN** | 3.03 (-4.40 to 10.46) | 10.87 (-0.13 to 21.77) | 3.13 (-8.86 to 15.14) | 5.32 (-6.27 to 16.83) | 1.54 (-6.72 to 9.60) | 6.84 (-1.81 to 15.59) | -0.46 (-10.37 to 9.40) |
| --- | --- | --- | --- | --- | --- | --- | --- |
| **-3.00 (-5.87 to -0.13)** | **Baseline treatment** | **13.90 (6.13 to 21.67)** | 6.16 (-3.01 to 15.32) | 8.34 (-0.28 to 16.97) | **4.56 (1.72 to 7.40)** | **9.87 (5.69 to 14.04)** | 2.57 (-3.60 to 8.75) |
| NA | **13.90 (10.29 to 17.51)** | **Baxdrostat** | -7.74 (-19.95 to 4.46) | -5.55 (-17.30 to 6.16) | **-9.34 (-17.83 to -1.11)** | -4.03 (-12.95 to 4.90) | **-11.33 (-21.37 to -1.15)** |
| NA | NA | NA | **Clonidine** | 2.19 (-10.76 to 14.86) | -1.59 (-11.32 to 7.66) | 3.71 (-4.68 to 11.95) | -3.59 (-14.80 to 7.56) |
| NA | **8.40 (3.12 to 13.68)** | NA | NA | **Darusentan** | -3.78 (-13.08 to 5.33) | 1.52 (-8.23 to 11.25) | -5.77 (-16.62 to 4.87) |
| NA | **4.45 (1.50 to 7.40)** | NA | NA | NA | **Radiofrequency RDN** | **5.30 (0.79 to 10.04)** | -1.99 (-8.81 to 4.92) |
| NA | **9.94 (7.25 to 12.62)** | NA | 3.70 (-0.86 to 8.26) | NA | 6.32 (-4.39 to 17.02) | **Spironolactone** | -7.30 (-14.86 to 0.21) |
| NA | 2.58 (-1.21 to 6.36) | NA | NA | NA | NA | NA | **Ultrasound RDN** |

Treatments are in alphabetical order. In the upper right triangle are presented results from the network meta-analysis. In the lower left triangle are presented results from pairwise meta-analyses (direct comparisons). Each cell provides mean difference and the corresponding 95% CrI (or CI for pairwise meta-analyses). Bold print indicates 95% CrIs excluding no effect. NA=not available. RDN=renal denervation.

- Pharmacological and device-based intervention classes

| **Aldosterone synthase inhibitors** | **-13.93 (-21.35 to -6.51)** | -5.54 (-16.76 to 5.81) | -4.22 (-12.64 to 4.45) | **-9.82 (-17.78 to -1.92)** | -7.94 (-19.59 to 3.90) |
| --- | --- | --- | --- | --- | --- |
| **-13.90 (-17.51 to -10.29)** | **Baseline treatment** | **8.39 (0.04 to 16.73)** | **9.71 (5.69 to 13.73)** | **4.11 (1.80 to 6.42)** | 5.99 (-2.87 to 14.86) |
| NA | **8.40 (3.12 to 13.68)** | **Endothelin receptor antagonists** | 1.32 (-7.93 to 10.81) | -4.28 (-12.99 to 4.49) | -2.39 (-14.62 to 10.01) |
| NA | **9.94 (7.25 to 12.62)** | NA | **Mineralocorticoid receptor antagonists** | **-5.60 (-10.05 to -1.38)** | -3.71 (-11.69 to 4.36) |
| NA | **4.02 (1.83 to 6.22)** | NA | -6.32 (-17.02 to 4.39) | **Renal**  **denervation** | 1.88 (-7.05 to 11.15) |
| NA | NA | NA | -3.70 (-8.26 to 0.86) | NA | **α-agonists** |

Treatments are in alphabetical order. In the upper right triangle are presented results from the network meta-analysis. In the lower left triangle are presented results from pairwise meta-analyses (direct comparisons). Each cell provides mean difference and the corresponding 95% CrI (or CI for pairwise meta-analyses). Bold print indicates 95% CrIs excluding no effect. NA=not available.

## 9.13 **Daytime diastolic blood pressure**

### 9.13.1 Network plot

- Individual pharmacological and device-based interventions

The lines link treatments that were directly compared in trials. The thickness of the lines corresponds to the number of trials evaluating the comparison. The size of the circles (nodes) corresponds to the number of participants assigned to the treatment. RDN=renal denervation.

- Pharmacological and device-based intervention classes

The lines link treatments that were directly compared in trials. The thickness of the lines corresponds to the number of trials evaluating the comparison. The size of the circles (nodes) corresponds to the number of participants assigned to the treatment.

### 9.13.2 Forest-plot of network meta-analysis

- Individual pharmacological and device-based interventions

Effect sizes are from the network meta-analysis. Treatments are ordered accordingly to the mean difference. The reference is the baseline treatment employed in each included trial. CrI=credible intervals. DBP=diastolic blood pressure. MD=mean difference. RDN=renal denervation.

- Pharmacological and device-based intervention classes

Effect sizes are from the network meta-analysis. Treatments are ordered accordingly to the mean difference. The reference is the baseline treatment employed in each included trial. CrI=credible intervals. DBP=diastolic blood pressure. MD=mean difference.

### 9.13.4 Forest-plot of pairwise meta-analysis

- Individual pharmacological and device-based interventions

Treatment comparisons are presented in alphabetical order. Summary effect size is calculated using pairwise meta-analysis of all studies in a specific comparison. CI=confidence interval. DBP=diastolic blood pressure. MD=mean difference. RDN=renal denervation.

- Pharmacological and device-based intervention classes

Treatment comparisons are presented in alphabetical order. Summary effect size is calculated using pairwise meta-analysis of all studies in a specific comparison. CI=confidence interval. DBP=diastolic blood pressure. MD=mean difference.

### 9.13.5 League table

- Individual pharmacological and device-based interventions

| **Baseline treatment** | **6.67 (1.46 to 11.87)** | 1.91 (-4.37 to 8.18) | **2.19 (0.31 to 4.06)** | **4.54 (1.65 to 7.43)** | 0.98 (-3.15 to 5.11) |
| --- | --- | --- | --- | --- | --- |
| **6.70 (4.61 to 8.79)** | **Baxdrostat** | -4.76 (-12.93 to 3.62) | -4.48 (-10.18 to 1.15) | -2.13 (-8.10 to 4.12) | -5.68 (-12.51 to 1.12) |
| NA | NA | **Clonidine** | 0.28 (-6.37 to 6.69) | 2.63 (-3.01 to 8.40) | -0.92 (-8.80 to 6.69) |
| **2.13 (0.48 to 3.78)** | NA | NA | **Radiofrequency RDN** | 2.35 (-0.57 to 5.67) | -1.21 (-5.75 to 3.40) |
| **4.46 (2.08 to 6.83)** | NA | 2.60 (-0.27 to 5.47) | 1.97 (-2.89 to 6.83) | **Spironolactone** | -3.56 (-8.81 to 1.48) |
| 0.97 (-1.26 to 3.19) | NA | NA | NA | NA | **Ultrasound RDN** |

Treatments are in alphabetical order. In the upper right triangle are presented results from the network meta-analysis. In the lower left triangle are presented results from pairwise meta-analyses (direct comparisons). Each cell provides mean difference and the corresponding 95% CrI (or CI for pairwise meta-analyses). Bold print indicates 95% CrIs excluding no effect. NA=not available. RDN=renal denervation.

- Pharmacological and device-based intervention classes

| **Aldosterone synthase inhibitors** | **-6.68 (-11.73 to -1.63)** | -2.24 (-8.03 to 3.92) | -4.70 (-10.13 to 0.74) | -4.85 (-12.86 to 3.27) |
| --- | --- | --- | --- | --- |
| **-6.70 (-8.79 to -4.61)** | **Baseline treatment** | **4.44 (1.65 to 7.22)** | **1.98 (0.32 to 3.64)** | 1.83 (-4.23 to 7.88) |
| NA | **4.46 (2.08 to 6.83)** | **Mineralocorticoid receptor antagonists** | -2.45 (-5.61 to 0.37) | -2.61 (-8.13 to 2.86) |
| NA | 1.93 (-0.54 to 3.32) | 1.97 (-2.89 to 6.83) | **Renal**  **denervation** | -0.16 (-6.20 to 6.18) |
| NA | NA | -2.60 (-5.47 to 0.27) | NA | **α-agonists** |

Treatments are in alphabetical order. In the upper right triangle are presented results from the network meta-analysis. In the lower left triangle are presented results from pairwise meta-analyses (direct comparisons). Each cell provides mean difference and the corresponding 95% CrI (or CI for pairwise meta-analyses). Bold print indicates 95% CrIs excluding no effect. NA=not available.

## 9.15 **Nighttime systolic blood pressure**

### 9.15.1 Network plot

- Individual pharmacological and device-based interventions

The lines link treatments that were directly compared in trials. The thickness of the lines corresponds to the number of trials evaluating the comparison. The size of the circles (nodes) corresponds to the number of participants assigned to the treatment. RDN=renal denervation.

- Pharmacological and device-based intervention classes

The lines link treatments that were directly compared in trials. The thickness of the lines corresponds to the number of trials evaluating the comparison. The size of the circles (nodes) corresponds to the number of participants assigned to the treatment.

### 9.15.2 Forest-plot of network meta-analysis

- Individual pharmacological and device-based interventions

Effect sizes are from the network meta-analysis. Treatments are ordered accordingly to the mean difference. The reference is the baseline treatment employed in each included trial. CrI=credible intervals. MD=mean difference. RDN=renal denervation. SBP=systolic blood pressure.

- Pharmacological and device-based intervention classes

Effect sizes are from the network meta-analysis. Treatments are ordered accordingly to the mean difference. The reference is the baseline treatment employed in each included trial. CrI=credible intervals. MD=mean difference. SBP=systolic blood pressure.

### 9.15.4 Forest-plot of pairwise meta-analysis

- Individual pharmacological and device-based interventions

Treatment comparisons are presented in alphabetical order. Summary effect size is calculated using pairwise meta-analysis of all studies in a specific comparison. CI=confidence interval. DBP=diastolic blood pressure. MD=mean difference. RDN=renal denervation.

- Pharmacological and device-based intervention classes

Treatment comparisons are presented in alphabetical order. Summary effect size is calculated using pairwise meta-analysis of all studies in a specific comparison. CI=confidence interval. DBP=diastolic blood pressure. MD=mean difference.

### 9.15.5 League table

- Individual pharmacological and device-based interventions

| **Alcohol RDN** | 3.34 (-3.60 to 10.27) | **9.90 (0.64 to 18.94)** | 4.92 (-7.01 to 17.11) | 5.26 (-6.20 to 16.47) | 0.76 (-7.11 to 8.22) | 7.59 (-0.72 to 15.91) | -1.81 (-11.43 to 7.84) |
| --- | --- | --- | --- | --- | --- | --- | --- |
| -3.30 (-6.60 to 0.00) | **Baseline treatment** | **13.23 (7.64 to 18.83)** | 8.25 (-1.47 to 17.97) | 8.59 (0.00 to 17.19) | **4.10 (1.33 to 6.86)** | **10.93 (6.52 to 15.33)** | 1.52 (-4.80 to 7.85) |
| NA | **13.60 (10.70 to 16.49)** | **Baxdrostat** | -4.98 (-16.33 to 6.47) | -4.64 (-15.01 to 5.93) | **-9.14 (-15.53 to -2.89)** | -2.31 (-9.42 to 5.11) | **-11.71 (-20.17 to -3.08)** |
| NA | NA | NA | **Clonidine** | 0.34 (-12.92 to 13.51) | -4.16 (-14.32 to 5.80) | 2.67 (-5.99 to 11.49) | -6.73 (-18.57 to 4.94) |
| NA | **8.60 (2.62 to 14.58)** | NA | NA | **Darusentan** | -4.50 (-13.72 to 4.51) | 2.33 (-7.43 to 12.27) | -7.07 (-17.86 to 3.81) |
| NA | **3.64 (1.01 to 6.27)** | NA | NA | NA | **Radiofrequency RDN** | **6.83 (2.20 to 11.80)** | -2.57 (-9.44 to 4.60) |
| NA | **12.41 (8.76 to 16.07)** | NA | 2.60 (-3.69 to 8.89) | NA | 3.14 (-4.93 to 11.20) | **Spironolactone** | **-9.40 (-17.26 to -1.65)** |
| NA | 1.54 (-3.00 to 6.07) | NA | NA | NA | NA | NA | **Ultrasound RDN** |

Treatments are in alphabetical order. In the upper right triangle are presented results from the network meta-analysis. In the lower left triangle are presented results from pairwise meta-analyses (direct comparisons). Each cell provides mean difference and the corresponding 95% CrI (or CI for pairwise meta-analyses). Bold print indicates 95% CrIs excluding no effect. NA=not available. RDN=renal denervation.

- Pharmacological and device-based intervention classes

| **Aldosterone synthase inhibitors** | **-13.28 (-18.49 to -8.06)** | -4.68 (-14.38 to 5.19) | -2.48 (-9.09 to 4.46) | **-9.56 (-15.32 to -3.73)** | -5.07 (-15.66 to 5.95) |
| --- | --- | --- | --- | --- | --- |
| **-13.60 (-16.49 to -10.70)** | **Baseline treatment** | **8.60 (0.46 to 16.73)** | **10.80 (6.65 to 14.95)** | **3.71 (1.47 to 5.96)** | 8.20 (-1.16 to 17.56) |
| NA | **8.60 (2.62 to 14.58)** | **Endothelin receptor antagonists** | 2.20 (-7.10 to 11.42) | -4.88 (-13.51 to 3.48) | -0.39 (-12.75 to 12.34) |
| NA | **12.41 (8.76 to 16.07)** | NA | **Mineralocorticoid receptor antagonists** | **-7.08 (-11.66 to -2.79)** | -2.59 (-11.03 to 5.85) |
| NA | NA | NA | -3.14 (-11.20 to 4.93) | **Renal**  **denervation** | 4.49 (-4.96 to 14.18) |
| NA | NA | NA | -2.60 (-8.89 to 3.69) | NA | **α-agonists** |

Treatments are in alphabetical order. In the upper right triangle are presented results from the network meta-analysis. In the lower left triangle are presented results from pairwise meta-analyses (direct comparisons). Each cell provides mean difference and the corresponding 95% CrI (or CI for pairwise meta-analyses). Bold print indicates 95% CrIs excluding no effect. NA=not available.

## 9.17 **Nighttime diastolic blood pressure**

### 9.17.1 Network plot

- Individual pharmacological and device-based interventions

The lines link treatments that were directly compared in trials. The thickness of the lines corresponds to the number of trials evaluating the comparison. The size of the circles (nodes) corresponds to the number of participants assigned to the treatment. RDN=renal denervation.

- Pharmacological and device-based intervention classes

The lines link treatments that were directly compared in trials. The thickness of the lines corresponds to the number of trials evaluating the comparison. The size of the circles (nodes) corresponds to the number of participants assigned to the treatment.

### 9.17.2 Forest-plot of network meta-analysis

- Individual pharmacological and device-based interventions

Effect sizes are from the network meta-analysis. Treatments are ordered accordingly to the mean difference. The reference is the baseline treatment employed in each included trial. CrI=credible intervals. DBP=diastolic blood pressure. MD=mean difference. RDN=renal denervation.

- Pharmacological and device-based intervention classes

Effect sizes are from the network meta-analysis. Treatments are ordered accordingly to the mean difference. The reference is the baseline treatment employed in each included trial. CrI=credible intervals. DBP=diastolic blood pressure. MD=mean difference.

### 9.17.4 Forest-plot of pairwise meta-analysis

- Individual pharmacological and device-based interventions

Treatment comparisons are presented in alphabetical order. Summary effect size is calculated using pairwise meta-analysis of all studies in a specific comparison. CI=confidence interval. DBP=diastolic blood pressure. MD=mean difference. RDN=renal denervation.

- Pharmacological and device-based intervention classes

Treatment comparisons are presented in alphabetical order. Summary effect size is calculated using pairwise meta-analysis of all studies in a specific comparison. CI=confidence interval. DBP=diastolic blood pressure. MD=mean difference.

### 9.17.5 League table

- Individual pharmacological and device-based interventions

| **Baseline treatment** | **6.79 (0.23 to 13.36)** | 3.41 (-4.41 to 11.23) | 1.98 (-0.35 to 4.31) | **5.83 (2.20 to 9.45)** | 0.69 (-4.45 to 5.82) |
| --- | --- | --- | --- | --- | --- |
| **6.80 (4.50 to 9.10)** | **Baxdrostat** | -3.38 (-13.75 to 7.38) | -4.82 (-12.03 to 2.37) | -0.97 (-8.54 to 7.05) | -6.11 (-14.73 to 2.43) |
| NA | NA | **Clonidine** | -1.44 (-9.82 to 6.34) | 2.41 (-4.69 to 9.44) | -2.73 (-12.45 to 6.69) |
| **1.69 (0.22 to 3.15)** | NA | NA | **Radiofrequency RDN** | **3.85 (0.16 to 7.98)** | -1.29 (-6.89 to 4.55) |
| **7.83 (2.92 to 12.75)** | NA | 2.40 (-0.92 to 5.72) | 0.09 (-3.89 to 4.06) | **Spironolactone** | -5.14 (-11.61 to 1.17) |
| 0.59 (-2.10 to 3.29) | NA | NA | NA | NA | **Ultrasound RDN** |

Treatments are in alphabetical order. In the upper right triangle are presented results from the network meta-analysis. In the lower left triangle are presented results from pairwise meta-analyses (direct comparisons). Each cell provides mean difference and the corresponding 95% CrI (or CI for pairwise meta-analyses). Bold print indicates 95% CrIs excluding no effect. NA=not available. RDN=renal denervation.

- Pharmacological and device-based intervention classes

| **Aldosterone synthase inhibitors** | **-6.78 (-13.06 to -0.50)** | -1.09 (-8.18 to 6.40) | -5.04 (-11.78 to 1.69) | -3.48 (-13.32 to 6.75) |
| --- | --- | --- | --- | --- |
| **-6.80 (-9.10 to -4.50)** | **Baseline treatment** | **5.69 (2.23 to 9.15)** | 1.75 (-0.29 to 3.79) | 3.31 (-4.31 to 10.92) |
| NA | 7.83 (2.92 to -12.75) | **Mineralocorticoid receptor antagonists** | **-3.94 (-7.87 to -0.38)** | -2.38 (-9.40 to 4.52) |
| NA | **1.54 (0.25 to 2.83)** | -0.09 (-4.06 to 3.89) | **Renal**  **denervation** | 1.56 (-6.09 to 9.63) |
| NA | NA | -2.40 (-5.72 to 0.92) | NA | **α-agonists** |

Treatments are in alphabetical order. In the upper right triangle are presented results from the network meta-analysis. In the lower left triangle are presented results from pairwise meta-analyses (direct comparisons). Each cell provides mean difference and the corresponding 95% CrI (or CI for pairwise meta-analyses). Bold print indicates 95% CrIs excluding no effect. NA=not available.

# 10. **Heterogeneity in the network meta-analysis of primary and secondary outcomes**

Details:

The table presents the common τ, the estimator of between-study heterogeneity. A comparison with the empirical distribution of τ was not possible because such data are not available for mean differences.

Summary of results:

However, the lack of an empirical distribution against which to benchmark these estimates limits the interpretability of our findings, and all conclusions should therefore be considered in light of this unchecked assumption.

| **Outcome** | **Group** | **Common τ**  **(NMA)** | **Empirical predictive distribution of τ** |
| --- | --- | --- | --- |
| Office SBP | Individual pharmacological and device-based interventions | 4.59  (0.34-4.98) | Not available for mean differences |
|  | Pharmacological and device-based intervention classes | 4.56  (0.32-4.99) | Not available for mean differences |
| Office DBP | Individual pharmacological and device-based interventions | 3.70  (0.68-4.90) | Not available for mean differences |
|  | Pharmacological and device-based intervention classes | 3.70  (0.65-4.88) | Not available for mean differences |
| 24-hour SBP | Individual pharmacological and device-based interventions | 2.90  (0.85-4.64) | Not available for mean differences |
|  | Pharmacological and device-based intervention classes | 2.88  (0.78-4.55) | Not available for mean differences |
| 24-hour DBP | Individual pharmacological and device-based interventions | 2.06  (0.75-3.77) | Not available for mean differences |
|  | Pharmacological and device-based intervention classes | 1.91  (0.68-3.45) | Not available for mean differences |
| Daytime SBP | Individual pharmacological and device-based interventions | 3.40  (0.86-4.88) | Not available for mean differences |
|  | Pharmacological and device-based intervention classes | 3.18  (0.87-4.81) | Not available for mean differences |
| Daytime DBP | Individual pharmacological and device-based interventions | 2.32  (0.83-4.21) | Not available for mean differences |
|  | Pharmacological and device-based intervention classes | 2.20  (0.79-4.02) | Not available for mean differences |
| Nighttime SBP | Individual pharmacological and device-based interventions | 2.87  (1.18-4.82) | Not available for mean differences |
|  | Pharmacological and device-based intervention classes | 2.56  (1.19-4.72) | Not available for mean differences |
| Nighttime DBP | Individual pharmacological and device-based interventions | 2.98  (0.97-4.77) | Not available for mean differences |
|  | Pharmacological and device-based intervention classes | 2.83  (0.97-4.72) | Not available for mean differences |

DBP=diastolic blood pressure. SBP=systolic blood pressure.

# 11. **Inconsistency in the network meta-analysis of primary and secondary outcomes**

Details:

For details see the main text. Local inconsistency was evaluated by Separating Indirect from Direct Evidence test, for each comparison (a p<0.1 for a difference between direct and indirect evidence was chosen as threshold indicative of an inconsistent comparison.^37^ Global inconsistency was evaluated using a design-by treatment test was used to evaluate the presence of global inconsistency.^38^

Summary of results:

No evidence of significant inconsistency in direct and indirect estimates was detected for each outcome analysed.

| **Outcome** | **Group** | **SIDE-test**  **(p<0.1)** | **Design-by-treatment test**  **(p-value)** | **Judgment** |
| --- | --- | --- | --- | --- |
| Office SBP | Individual pharmacological and device-based interventions | 0% | 0.80 | No evidence of inconsistency |
|  | Pharmacological and device-based intervention classes | 0% | 0.62 | No evidence of inconsistency |
| Office DBP | Individual pharmacological and device-based interventions | 0% | 0.70 | No evidence of inconsistency |
|  | Pharmacological and device-based intervention classes | 0% | 0.47 | No evidence of inconsistency |
| 24-hour SBP | Individual pharmacological and device-based interventions | 0% | 0.70 | No evidence of inconsistency |
|  | Pharmacological and device-based intervention classes | 0% | 0.74 | No evidence of inconsistency |
| 24-hour DBP | Individual pharmacological and device-based interventions | 0% | 0.50 | No evidence of inconsistency |
|  | Pharmacological and device-based intervention classes | 0% | 0.42 | No evidence of inconsistency |
| Daytime SBP | Individual pharmacological and device-based interventions | 0% | 0.85 | No evidence of inconsistency |
|  | Pharmacological and device-based intervention classes | 0% | 0.71 | No evidence of inconsistency |
| Daytime DBP | Individual pharmacological and device-based interventions | 0% | 0.75 | No evidence of inconsistency |
|  | Pharmacological and device-based intervention classes | 0% | 0.69 | No evidence of inconsistency |
| Nighttime SBP | Individual pharmacological and device-based interventions | 0% | 0.19 | No evidence of inconsistency |
|  | Pharmacological and device-based intervention classes | 0% | 0.13 | No evidence of inconsistency |
| Nighttime DBP | Individual pharmacological and device-based interventions | 0% | 0.10 | No evidence of inconsistency |
|  | Pharmacological and device-based intervention classes | 1% | 0.08 | No evidence of inconsistency |

ABPM=ambulatory blood pressure monitoring. DBP=diastolic blood pressure. SBP=systolic blood pressure.

# 12. **Assessment of small-trial/publication bias**

We assessed the small-study/publication bias using a contour-enhanced funnel plot for the primary outcome, systolic blood pressure. This approach was appropriate because systolic blood pressure was also the main outcome reported in most of the included studies. As such, it is unlikely that studies were unpublished due to unfavourable results in secondary outcomes.

The small-trial/publication bias was based on the comparison between renal denervation and baseline treatment because it was the only comparison including more than 10 studies. In view of this limitation, we did not perform a comparison-adjusted funnel-plot.

Contour-enhanced funnel plot:

Most trials showed an equal distribution in the funnel plot. Accordingly, there is no indication of significative publication bias.

# 13. **Assessment of risk of bias**

We assessed risk of bias according to the Cochrane Risk of Bias Tool version 2. Although risk of bias is outcome specific, our assessment was based on our primary outcome (i.e systolic blood pressure) for feasibility reasons. When data for the primary outcome were not available, the assessment was based on 24-hour SBP. In the presence of multiple comparisons for a single study, we rated the overall risk of bias accordingly to that of the comparison with the worst risk of bias.

Risk of bias overall:

Risk of bias by study:

# 14. **Assessment of confidence in estimates**

We evaluated the confidence in estimates for each outcome by using the CINeMA framework^39^ implemented in the official webtool at <https://cinema.ispm.unibe.ch/>.

The CINeMA framework evaluates the following six different domains to rate the confidence in network meta-analysis results:

**Domain 1: Within-study bias**

We assessed the risk of bias for included studies using the Cochrane risk of bias tool 2 (see section 11) basing on our primary outcome, office SBP. When data for the primary outcome were not available, the assessment was based on 24-hour SBP. When multiple comparisons were available for a single study, we rated the overall risk of bias accordingly to that of the comparison with the worst risk of bias.

The CINeMA framework combines the contribution of a study with the risk of bias judgement to evaluate within-study bias for each estimate in the network meta-analysis. Notably, we used the most frequent risk of bias judgment within each comparison to define the corresponding within-study bias (“Majority RoB” option in CINeMA).

**Domain 2: Reporting bias**

We assessed potential reporting bias due to (i) suppression of negative findings (publication bias), (ii) omission of unfavorable results from study reports (outcome reporting bias) or (iii) delayed publication (time-lag bias).

Publication bias: see section 10.

Outcome reporting bias: we used the recommendations provided in the Cochrane handbook and the ORBIT-framework^40^ to evaluate outcome reporting bias.

Time-lag bias: baxdrostat and lorundrostat are recently investigated treatments in randomized clinical trials. Therefore, they are potentially subject to time-lag bias. Accordingly, comparisons involving these agents were rated as having some concerns with respect to reporting bias.

**Domain 3: Indirectness**

For this domain it should be judged whether study populations, interventions, outcomes, and study settings are representative of the ones relevant for the research questions.

We used stringent inclusion criteria for our research question; accordingly, no indirectness is identified.

Population: we included studies of patients with uncontrolled hypertension taking at least two antihypertensive medications. Therefore, we considered the study populations as directly relevant for the research question.

Intervention: each investigated treatment is directly relevant for the research question.

Outcome: each included outcome is objective and directly relevant for the research question.

Setting: we included studies conducted with out-patients, in-patients or both. Each setting may be relevant to the research question.

**Domain 4: Imprecision**

We considered MD outside the range of -10 to 10 mmHg as clinically important. This threshold was chosen based on the findings of the meta-analysis by Ettehad et al.,^41^ which identified a reduction of 10 mmHg in systolic blood pressure as clinically significant for lowering the risk of cardiovascular events.

**Domain 5: Heterogeneity**

We considered MD outside the range of -10 to 10 mmHg as clinically important. This threshold was chosen based on the findings of the meta-analysis by Ettehad et al.,^41^ which identified a reduction of 10 mmHg in systolic blood pressure as clinically significant for lowering the risk of cardiovascular events. Heterogeneity was evaluated based on the relation of both the 95% confidence intervals and the 95% prediction intervals with the clinically meaningful threshold defined above in the domain of imprecision.

**Domain 6: Incoherence**

We considered MD outside the range of -10 to 10 mmHg as clinically important. This threshold was chosen based on the findings of the meta-analysis by Ettehad et al.,^41^ which identified a reduction of 10 mmHg in systolic blood pressure as clinically significant for lowering the risk of cardiovascular events. Incoherence was evaluated using a design-by-treatment test and the SIDE according to CINeMA documentation.

**Overall CINeMA level**

In the CINeMA framework, the overall level of confidence can be judged as following:

1. High

2. Moderate

3. Low

4. Very low

The CINeMA guidance document suggests downgrading the overall certainty of evidence by one level for each domain with “some concerns” and two levels for each domain with “major concerns”. However, in case, several domains are rated at some concerns or major concerns, it is recommended to consider judgements on different domains jointly rather than in isolation.

To account for these recommendations, we used the approach proposed by Schneider-Thoma et al.^42^ to rate overall the confidence in estimates:

- 1 judgement of “some concerns” leads to downgrading by 1 level.

- 1 judgement of “major concerns” leads to downgrading by 2 levels.

- 2 judgements of “some concerns” could be interconnected and do not justify downgrading more than by 1 level.

- 1 judgement of “major concerns” and up to 2 judgements of “some concerns” or 1 additional judgement of “major concerns” could be interconnected and do not justify downgrading by more than 2 levels.

- 2 judgements of “major concerns” and any additional judgements of “some concerns” or “major concerns” (or more than 4 judgements of some concerns) lead to downgrading by three levels (the maximum).

The results of the CINeMA assessment for each comparison and outcome are presented using a colour code in the league tables of each outcome.

# 15. **References**

1.	Azizi, M. *et al.* Effect of baxdrostat on ambulatory blood pressure in patients with resistant hypertension (Bax24): a phase 3, randomised, double-blind, placebo-controlled trial. *Lancet* **407**, 988–999 (2026).

2.	Flack, J. M. *et al.* Efficacy and Safety of Baxdrostat in Uncontrolled and Resistant Hypertension. *New England Journal of Medicine* Epub ahead of print. (2025) doi:10.1056/NEJMoa2507109.

3.	Freeman, M. W. *et al.* Phase 2 Trial of Baxdrostat for Treatment-Resistant Hypertension. *New England Journal of Medicine* **388**, 395–405 (2023).

4.	Laffin, L. J. *et al.* Lorundrostat Efficacy and Safety in Patients with Uncontrolled Hypertension. *New England Journal of Medicine* **392**, 1813–1823 (2025).

5.	Saxena, M. *et al.* Lorundrostat in Participants With Uncontrolled Hypertension and Treatment-Resistant Hypertension. *JAMA* **334**, 409–418 (2025).

6.	Laffin, L. J. *et al.* Aldosterone Synthase Inhibition With Lorundrostat for Uncontrolled Hypertension. *JAMA* **330**, 1140–1150 (2023).

7.	KARDIA-3: Zilebesiran as add-on therapy in adults with hypertension and established cardiovascular disease or at high cardiovascular risk’ presented during HOT LINE 4 on 30 August 2025 at 16:30 to 16:40 in Madrid .

8.	Schlaich, M. P. *et al.* Dual endothelin antagonist aprocitentan for resistant hypertension (PRECISION): a multicentre, blinded, randomised, parallel-group, phase 3 trial. *Lancet* **400**, 1927–1937 (2022).

9.	Black, H. R. *et al.* Efficacy and Safety of Darusentan in Patients With Resistant Hypertension: Results From a Randomized, Double‐Blind, Placebo‐Controlled Dose‐Ranging Study. *J. Clin. Hypertens.* **9**, 760–769 (2007).

10.	Weber, M. A. *et al.* A selective endothelin-receptor antagonist to reduce blood pressure in patients with treatment-resistant hypertension: a randomised, double-blind, placebo-controlled trial. *Lancet* **374**, 1423–1431 (2009).

11.	Bakris, G. L. *et al.* Divergent Results Using Clinic and Ambulatory Blood Pressures. *Hypertension* **56**, 824–830 (2010).

12.	Krieger, E. M. *et al.* Spironolactone Versus Clonidine as a Fourth-Drug Therapy for Resistant Hypertension. *Hypertension* **71**, 681–690 (2018).

13.	Abolghasmi, R. & Taziki, O. *Efficacy of Low Dose Spironolactone in Chronic Kidney Disease with Resistant Hypertension*. *Saudi J Kidney Dis Transplant* vol. 22 (2011).

14.	Václavík, J., Sedlák, R., Jarkovský, J., Kociánová, E. & Táborský, M. Effect of Spironolactone in Resistant Arterial Hypertension. *Medicine* **93**, e162 (2014).

15.	Djoumessi, R. N. *et al.* Effect of low-dose spironolactone on resistant hypertension in type 2 diabetes mellitus: a randomized controlled trial in a sub-Saharan African population. *BMC Res. Notes* **9**, 187 (2016).

16.	Ni, X. *et al.* Effects of Spironolactone on Dialysis Patients With Refractory Hypertension: A Randomized Controlled Study. *J. Clin. Hypertens.* **16**, 658–663 (2014).

17.	Oxlund, C. S. *et al.* Low dose spironolactone reduces blood pressure in patients with resistant hypertension and type 2 diabetes mellitus. *J. Hypertens.* **31**, 2094–2102 (2013).

18.	Yang, L. *et al.* Effect of spironolactone on patients with resistant hypertension and obstructive sleep apnea. *Clin. Exp. Hypertens.* **38**, 464–468 (2016).

19.	Eguchi, K., Kabutoya, T., Hoshide, S., Ishikawa, S. & Kario, K. Add‐On Use of Eplerenone Is Effective for Lowering Home and Ambulatory Blood Pressure in Drug‐Resistant Hypertension. *The Journal of Clinical Hypertension* **18**, 1250–1257 (2016).

20.	Oliveras, A. *et al.* Spironolactone versus sympathetic renal denervation to treat true resistant hypertension. *J. Hypertens.* **34**, 1863–1871 (2016).

21.	Rosa, J. *et al.* Randomized Comparison of Renal Denervation Versus Intensified Pharmacotherapy Including Spironolactone in True-Resistant Hypertension. *Hypertension* **65**, 407–413 (2015).

22.	Kandzari, D. E. *et al.* Effect of Alcohol-Mediated Renal Denervation on Blood Pressure in the Presence of Antihypertensive Medications: Primary Results From the TARGET BP I Randomized Clinical Trial. *Circulation* **149**, 1875–1884 (2024).

23.	Azizi, M. *et al.* Optimum and stepped care standardised antihypertensive treatment with or without renal denervation for resistant hypertension (DENERHTN): a multicentre, open-label, randomised controlled trial. *Lancet* **385**, 1957–1965 (2015).

24.	Desch, S. *et al.* Randomized Sham-Controlled Trial of Renal Sympathetic Denervation in Mild Resistant Hypertension. *Hypertension* **65**, 1202–1208 (2015).

25.	Jiang, X. *et al.* Efficacy and Safety of Catheter-Based Radiofrequency Renal Denervation in Chinese Patients With Uncontrolled Hypertension: The Randomized, Sham-Controlled, Multi-Center Iberis-HTN Trial. *Circulation* **150**, 1588–1598 (2024).

26.	Li, Y. *et al.* The Netrod^TM^ six-electrode radiofrequency renal denervation system for uncontrolled hypertension: a sham-controlled trial. *Eur. Heart J.* **45**, 4761–4764 (2024).

27.	Azizi, M. *et al.* Ultrasound renal denervation for hypertension resistant to a triple medication pill (RADIANCE-HTN TRIO): a randomised, multicentre, single-blind, sham-controlled trial. *Lancet* **397**, 2476–2486 (2021).

28.	Peters, C. D. *et al.* The effect of renal denervation on arterial stiffness, central blood pressure and heart rate variability in treatment resistant essential hypertension: a substudy of a randomized sham-controlled double-blinded trial (the ReSET trial). *Blood Press.* **26**, 366–380 (2017).

29.	Wang, J. *et al.* Efficacy and safety of sympathetic mapping and ablation of renal nerves for the treatment of hypertension (SMART): 6-month follow-up of a randomised, controlled trial. *EClinicalMedicine* **72**, 102626 (2024).

30.	Kandzari, D. E. *et al.* Effect of renal denervation on blood pressure in the presence of antihypertensive drugs: 6-month efficacy and safety results from the SPYRAL HTN-ON MED proof-of-concept randomised trial. *Lancet* **391**, 2346–2355 (2018).

31.	De Jager, R. L. *et al.* Impact of Medication Adherence on the Effect of Renal Denervation: The SYMPATHY Trial. *Hypertension* **69**, 678–684 (2017).

32.	Esler, M. D., Krum, H., Sobotka, P. A., Schlaich, M. P. & Schmieder, R. E. Renal sympathetic denervation in patients with treatment-resistant hypertension (The Symplicity HTN-2 Trial): a randomised controlled trial. *Lancet* **376**, 1903–1909 (2010).

33.	Bhatt, D. L. *et al.* A Controlled Trial of Renal Denervation for Resistant Hypertension. *New England Journal of Medicine* **370**, 1393–1401 (2014).

34.	Kario, K. *et al.* Catheter-based ultrasound renal denervation in patients with resistant hypertension: the randomized, controlled REQUIRE trial. *Hypertension Research* **45**, 221–231 (2022).

35.	Schmieder, R. E. *et al.* Phase II randomized sham-controlled study of renal denervation for individuals with uncontrolled hypertension – WAVE IV. *J. Hypertens.* **36**, 680–689 (2018).

36.	Tian, Z. *et al.* Efficacy of pharmacological and interventional treatment for resistant hypertension: a network meta-analysis. *Cardiovasc. Res.* **120**, 108–119 (2024).

37.	Dias, S., Welton, N. J., Caldwell, D. M. & Ades, A. E. Checking consistency in mixed treatment comparison meta‐analysis. *Stat. Med.* **29**, 932–944 (2010).

38.	Higgins, J. P. T. *et al.* Consistency and inconsistency in network meta‐analysis: concepts and models for multi‐arm studies. *Res. Synth. Methods* **3**, 98–110 (2012).

39.	Nikolakopoulou, A. *et al.* CINeMA: An approach for assessing confidence in the results of a network meta-analysis. *PLoS Med.* **17**, e1003082 (2020).

40.	Kirkham, J. J. *et al.* Outcome reporting bias in trials: a methodological approach for assessment and adjustment in systematic reviews. *BMJ* k3802 (2018) doi:10.1136/bmj.k3802.

41.	Ettehad, D. *et al.* Blood pressure lowering for prevention of cardiovascular disease and death: a systematic review and meta-analysis. *Lancet* **387**, 957–967 (2016).

42.	Schneider-Thoma, J. *et al.* Comparative efficacy and tolerability of 32 oral and long-acting injectable antipsychotics for the maintenance treatment of adults with schizophrenia: a systematic review and network meta-analysis. *The Lancet* **399**, 824–836 (2022).
